# Large-scale changes in marine and terrestrial environments drive the population dynamics of long-tailed ducks breeding in Siberia

**DOI:** 10.1038/s41598-022-16166-7

**Published:** 2022-07-19

**Authors:** J. Rintala, M. Hario, K. Laursen, A. P. Møller

**Affiliations:** 1 Devecto Oy, Vapaaherrantie 2, 40100 Jyväskylä, Finland; 2 Natural Resources Institute Finland, Latokartanonkaari 9, 00790 Helsinki, Finland; 3 Department of Ecoscience, Aarhus University, C.F. Møllers Alle, 8000 Aarhus C, Denmark; 4 Ecologie Systématique Evolution, AgroParisTech, Université Paris-Sud, CNRS, Université Paris-Saclay, 91405 Orsay Cedex, France

**Keywords:** Ecological modelling, Population dynamics, Environmental impact, Bayesian inference, Time series

## Abstract

Migratory animals experience very different environmental conditions at different times of the year, *i.e*., at the breeding grounds, during migration, and in winter. The long-tailed duck *Clangula hyemalis* breeds in the Arctic regions of the northern hemisphere and migrates to temperate climate zones, where it winters in marine environments. The breeding success of the long-tailed duck is affected by the abundances of predators and their main prey species, lemmings *Lemmus sibiricus* and *Dicrostonyx torquatus*, whose population fluctuation is subject to climate change. In the winter quarters, long-tailed ducks mainly eat the blue mussel *Mytilus edulis*. We examined how North-west Siberian lemming dynamics, assumed as a proxy for predation pressure, affect long-tailed duck breeding success and how nutrient availability in the Baltic Sea influences long-tailed duck population size via mussel biomass and quality. Evidence suggests that the long-tailed duck population dynamics was predator-driven on the breeding grounds and resource-driven on the wintering grounds. Nutrients from fertilizer runoff from farmland stimulate mussel stocks and quality, supporting high long-tailed duck population sizes. The applied hierarchical analysis combining several trophic levels can be used for evaluating large-scale environmental factors that affect the population dynamics and abundance of migrants from one environment to another.

## Introduction

Migratory organisms spend part of the year at the breeding grounds before moving to winter quarters that are often located thousands of kilometres away^1^. Changes in environmental conditions at the breeding grounds or winter quarters can influence performance of migrants in the same or the other habitat^2,3^. The long-tailed duck *Clangula hyemalis* is globally threatened and classified as Vulnerable on the IUCN Red List. It winters in North Europe and migrates along the Gulf of Finland to and from the breeding areas in Western Siberia^4^. Spring migration counts at Söderskär, Finland in the 1940s and 1950s showed a 90% reduction of the long-tailed duck population in the Baltic Sea wintering area^5,6^. During the Second World War, oil spills were suggested to be the main reason for the decline^5,7,8^. The wintering population gradually recovered as environmental conditions improved, with over half a million migrants. Spring counts peaked during 1991–1996 in Finland^8^ and Estonia^9^, followed by severe declines during the 2000s with recent numbers of migrants being only one-third of peak-year numbers (190,000 vs. 570,000 individuals). The North-west Siberian/North European winter population of the long-tailed duck was estimated at 4,700,000 birds based on co-ordinated surveys in 1992–1993^6,10^. By 2009, the Baltic Sea winter population had declined from 4,272,000 to 1,482,000 birds^6^.

Population characteristics of many species of aquatic birds that breed in the Arctic covary^11–15^ with population cycles of Arctic lemmings (*Lemmus sibiricus* and *Dicrostonyx torquatus*). These rodents are important prey for Arctic foxes (*Vulpes lagopus*), skuas and raptors, and thus form an essential component of the Arctic ecosystem^16^. Vole and lemming populations exhibit cyclic dynamics, particularly in northern regions^17^, with population peaks occurring every 3–5 years^11,18,19^ often followed by almost total absence of individuals^11,18,20–22^. Years with high abundance of lemmings lead to an increase in predator populations that show cycles that track those of their rodent prey^23–26^ such that predators are abundant in the next year following a rodent peak year while their populations are low after low lemming-abundance years^20,27^. In the year following a rodent peak, when predator populations are high, these predators exploit alternative prey^8,12,13,15,28,29^ such as bird eggs and young of species such as the long-tailed duck^12,30,31^, leading to large-scale breeding failure in these birds^12,32,33^ and fewer juveniles recruiting into the winter population^8,11,34^.

North-west Siberian/North European long-tailed ducks have declined since the 1990s^6^, coinciding with reductions in lemming abundances throughout the Arctic and northern alpine areas^35–37^ that has been linked to global climate warming, which particularly affects the Arctic. Indeed, changes in snow conditions due to climate change may explain why lemming populations show fewer peaks^38^. Snow hardness and humidity in the High Arctic, influenced by changing weather conditions, are critical for winter survival and reproduction in lemmings^38–40^.

The blue mussel, *Mytilus edulis*, constitutes the main food item for long-tailed ducks during winter^41–43^. Wintering success of long-tailed ducks declines with declining stocks of *M. edulis* in the southern Baltic Sea^44^. Mussel abundance is affected by climate, with cold winter temperatures stimulating reproduction in blue mussels, which may increase mussel stock sizes and thus sea duck populations a few years later^45^. Mussel abundance is also affected by fertiliser runoff in two ways. Fertilizer runoff from farmland increases dissolved nutrient levels, which increases primary production in the Baltic Sea^46,47^ and thus stimulates a positive bottom-up effect in coastal areas^45,48,49^. However, this also causes hypoxia and bottom death (*i.e.* the death of invertebrates and vertebrates in marine environments caused by anoxic or hypoxic conditions), which reduces abundance and availability of mussels in large areas of the Baltic Sea where there is poor mixing or influx of fresh, oxygenated water^46^ and where long-tailed ducks spend the winter.

Here we identify and quantify the ecological processes and their contributing environmental factors that influence the population dynamics of long-tailed ducks during both breeding and winter. For processes occurring at the breeding grounds, we investigated how the proxy for predation risk, *i.e.* lemming abundances in the previous year, and variability in precipitation and temperature in North-western Siberia influenced the proportion of juveniles at the wintering grounds in the southern Baltic Sea. For the processes occurring at the wintering grounds, we analysed how nutrient amounts, in the form of dissolved nitrogen and phosphorus, and winter climate influenced long-tailed duck juvenile proportions and spring migration population size. Using a hierarchical Bayesian^50,51^ model, implemented with the above-listed environmental variables, we suggest how lemming dynamics likely affected the proportion of juveniles in autumn and winter and how these together with nutrient availability explained migration numbers of the long-tailed duck in the following spring. This study exemplifies how to analyse complex dynamical systems based on heterogeneous and partly incomplete time-series data.

## Materials and methods

### Ethics declaration

This article does not contain any experiments on animal subjects performed by any of the authors.

### Observations of long-tailed ducks

**Figure 1:**
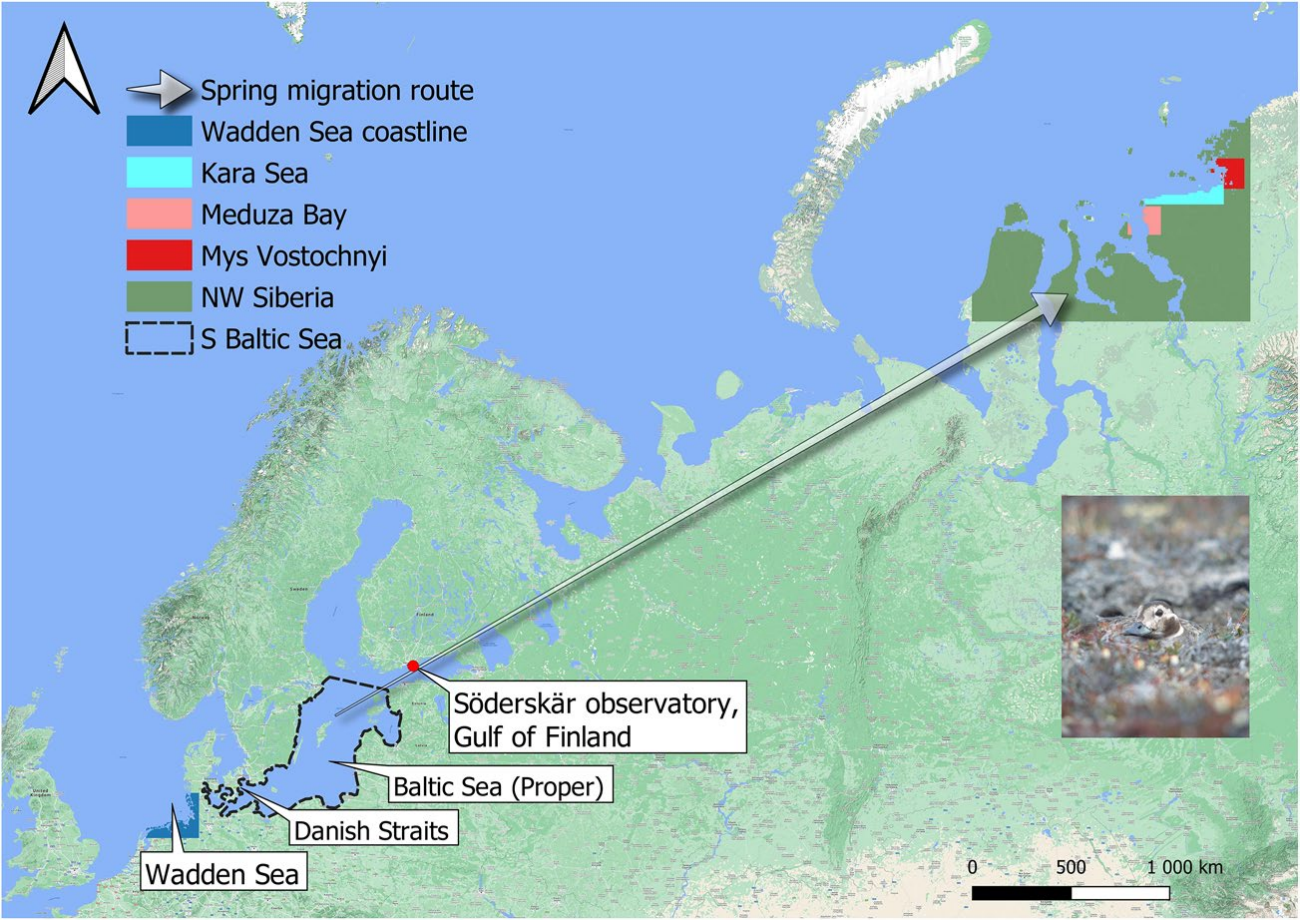
Study areas from the Wadden Sea, Denmark, and the Gulf of Finland extending to the Western Taimyr Peninsula, North-western Siberia, Russia. Colours indicate regions for which map-based climatological variables were calculated. The coastal areas of the southern Baltic Sea form the main wintering area of long-tailed ducks breeding in North-western Siberia. The spring migration surveys were conducted at Söderskär. Mussels were surveyed on the Wadden Sea, and lemmings in the Western Taimyr Peninsula: Kara Sea, Meduza Bay, and Mys Vostochnyi. This map was created using the free and open source QGIS (version 3.12 ‘București’, https://www.qgis.org/). Female long-tailed duck image by Antti Below.

Spring migration counts of long-tailed ducks have taken place at Söderskär (60°07′ N, 25°24′ E, Fig. 1), Gulf of Finland each spring 1968–2014. These counts are generally assumed to reflect population size^8^, and there is no indication that the main migration corridor along the northern coast of the Gulf of Finland (Fig. 1) for Baltic long-tailed ducks has changed during the study. However, unfavourable wind direction and velocity can force migrating birds off their preferred migration route in the vicinity of the Söderskär observatory^52,53^, thereby causing variation in migrant bird counts. To control for this error source, we used daily wind direction and velocity measures at Söderskär during spring migration surveys. We developed a weighted wind direction and velocity factor to be implemented into the observation model of the long-tailed duck to correct long-tailed duck population size estimates by reducing noise due to the effect of wind on spring migration counts^54,55^ (details in Supplementary Methods).

We estimated duck recruitment from the number of juvenile (1st to 2nd calendar year individuals) in relation to the number of adult female birds shot by hunters in autumn and winter (juvenile proportion in year *t* refers to bags from October in year *t* until February in year *t* + 1) from the Danish Wing Survey data^56,57^. We then related this to lemming abundance (of the year *t*–1) in North-western Siberia during the breeding period and climate conditions in breeding and wintering grounds (Supplementary Methods). Hunted long-tailed ducks in Danish waters belong to the North-west Siberian breeding population with the majority of long-tailed ducks wintering in the southern Baltic Sea^6,10^, and these birds migrate along the Gulf of Finland also including the Söderskär observatory^8,52^. Thus, we assumed that the Danish wing samples represent environmental impacts at the breeding grounds. These processes should be reflected to spring migration numbers as well.

### Environmental variables

1. Nutrient levels. Dissolved inorganic nitrogen (DIN), considering the sum of the oxidized nitrogen and ammonium pool, and dissolved inorganic phosphorus (DIP) were estimated for the Baltic Sea major basins each year from 1970 to 2016^47^. As the variables for nutrient availability at the main wintering area of the long-tailed duck, we used the annual totals of DIN and DIP recorded from two major basins in the southern Baltic Sea, the Baltic Proper and the Danish Straits^47^ (Fig. 1). Nutrients can have positive or negative effects on the long-tailed duck population size. Additional DIN stimulates primary production and thereby growth of mussels, the primary food of this species during winter^41–43^. Negative effects can arise because of hypoxia and bottom death that can occur at low DIN:DIP ratios^47^ in areas of poor mixing^46^. The total annual amount of fertilizer applied by Danish farmers is a reliable proxy for total nitrogen and phosphorus runoff into the marine environment^48,58^ (see Supplementary Methods). Here, we estimate how fertilizer use (in 1965–2016) is related to the trajectories of DIN and DIP and quantify its likely impact on the long-tailed duck dynamics in the southern Baltic Sea.
2. Lemming abundances. The majority of long-tailed ducks wintering in the Baltic Sea originate from the part of North-western Siberia that includes the Yamal and Taimyr Peninsulas^59^. Because of the presumed indirect effect on fox predation, lemming abundance across the entire breeding area should influence the dynamics of the long-tailed duck population; unfortunately, such data are lacking. However, information on lemming abundance exists from three separate, long-term surveys in the Western Taimyr Peninsula which allow us to quantify regional population changes in lemmings (Fig. 2). One survey was performed within a zone of ca. 100 km along the coast of the Kara Sea, Western Taimyr, between 80 and 90° E^11,19^. Two other surveys took place on the Western Taimyr Peninsula^18^ (survey no. 44, Meduza Bay, 73° 04′ N, 80° 30′ E; and survey no. 45, Mys Vostochnyi, 74° 06′ N, 86° 48′ E) (see Supplementary Methods).

**Figure 2:**
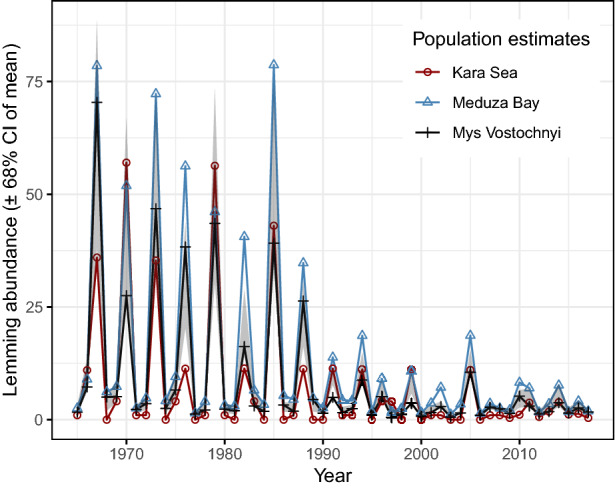
Dynamics of three lemming populations in the Western Taimyr Peninsula (population size estimates). The confidence interval (CI) is related to annual mean values based on the three lemming monitoring programs: Kara Sea, Meduza Bay, and Mys Vostochnyi.
3. North-west Siberian climate. We extracted annual (1965–2017) monthly climatological variables (precipitation and temperature) from a database of high spatial resolution global weather and climate data^60,61^ (data downloaded from www.worldclim.org) for the three regions in Western Taimyr for which lemming abundance data were available. Climate variation was averaged for the three areas corresponding to the three rodent surveys (Fig. 1). We estimated climate variability impacts on rodent population sizes^38^, which in turn may influence the breeding success and population dynamics of the long-tailed duck^8^. Similarly, for estimating climate-related consequences on long-tailed duck juvenile proportions, we averaged annual precipitation and temperature scores for North-western Siberia (Fig. 1). For processing the climatological map data, we used the *R* package ‘raster’^62^.
4. North Atlantic Oscillation Index (NAOI^63^). Winter NAOI (December in year *t*–1 until February in year *t*) is an index of the severity of winter conditions in the Northern Atlantic^63,64^. High index values indicate above average winter temperatures and high levels of precipitation in the Baltic Sea^64^. We investigated the association between winter NAOI and the proportion of juveniles killed by Danish hunters and ultimately the population dynamics of the long-tailed duck.
5. Abundance and quality of blue mussels. At the wintering grounds, long-tailed ducks feed on blue mussels, but no long-term data exist for mussel stocks in the Baltic Sea. Comparable data are available from the Wadden Sea, where annual blue mussel stocks were estimated for the intertidal zone of the Danish (1986–2007, and 2017) and Schleswig–Holstein (Germany, 1998–2015) parts of the Wadden Sea (Fig. 1) during autumn by ground sampling on mussel beds and estimation of mussel beds from aerial photography^65,66^. Mussel stock data are available for both areas from 1998 to 2007 and annual biomass showed a positive correlation between the areas (*r* = 0.920, *N* = 10). Blue mussel quality data, measured as flesh to shell ratio^67,68^, were available for each autumn from 1998 to 2013 from 19 sites in the Baltic and Wadden Sea (see Supplementary Methods).

The region of the mussel surveys are outside the core winter grounds of the long-tailed duck^6^. Thus, we did not accept the mussel data in the ultimate model for long-tailed duck population dynamics. Instead, we aimed at indicating the relation between nutrient load (in the form of fertilizer use in the Danish farmland^58^) and blue mussel stocks on the Wadden Sea^69^. A relation found between fertilizers and mussels on the Wadden Sea would indicate that the availability of dissolved nutrients (DIN and DIP as linked to fertilizer use^58^) can be used as a proxy for mussel abundance and quality in the southern Baltic Sea. The results shown for other seabird populations in that region^70,71^ support this reasoning.

We also aimed at showing how winter and spring temperatures affect blue mussel stocks on the Wadden Sea. For these, we calculated regional temperatures using the same global climate database as described above (see “North-west Siberian climate”). In the Baltic Sea, in the western part of the Finnish Archipelago, blue mussels grow from larvae to 10 mm within 3–4 years^72^ so we expect mussels in the southern Baltic Sea ecosystem to attain the size of 9 mm preferred by long-tailed ducks^44^ in about the same period after spawning. Hence, assuming that increasing winter temperatures reduce mussel quality^68^ and energy for spawning^70^, a reduction in 9-mm mussel cohorts should emerge a few years after mild winters.

### Hierarchical modelling

We used an integrated hierarchical model^73^, which is a complex stochastic system partitioned into a dependent sequential set of simpler sub-models dynamically affecting the performance of the main system of interest^73^ (Supplementary Methods, Supplementary Table S1).

Lemming population dynamics ${z}_{\left(t,j\right)}$, combining three separate population time-series $\left(j\right)$ of lemming abundances in the Western Taimyr Peninsula (1965–2017, $t=0$ refers to 1965), were estimated based on the following state-space model:

1 $$ \begin{array}{*{20}c} {\hat{z}_{{\left( {t,1} \right)}} = a_{z} + c_{z} z_{{\left( {t - 1,1} \right)}} + \sum\limits_{k = 1}^{2} {b_{\left( C \right)k} C_{{\left( {t,k,1} \right)}} + {\varvec{X}}_{1} a + {\varvec{X}}_{2} b + b_{t } \left( {t - 1} \right) + \in_{{z\left( {t,1} \right)}} } } \\ {\hat{z}_{{\left( {t,j = 2|j = 3} \right)}} = \hat{z}_{{\left( {t,1} \right)}} + \delta_{j} + \mathop \sum \limits_{k = 1}^{2} \theta_{k,j} C_{{\left( {t,k,j} \right)}} + \in_{{z\left( {t,j} \right)}} } \\ \end{array} $$

in which random variation in lemming population size estimates, ${z}_{\left(t,j\right)}$, are negative binomially distributed around the expectation, ${\widehat{z}}_{\left(t,1\right)}$ (Eq. (1), detailed in Supplementary Methods). ${a}_{z}$ is the intercept for $j=1$, and ${c}_{z}$ is a density dependent parameter^51^. The parameter ${b}_{\left(C\right)k}$ controls for the response of population $\left(j=1\right)$ to climate variation (${C}_{\left(t,k,j=1\right)})$. Subscript $k$ indicates precipitation in summer $\left(t,k=1\right)$ and the previous autumn $\left(t-1,k=2\right)$ for each population $j$. Parameter vectors ${\varvec{a}}$ and ${\varvec{b}}$ measure the consistency in three-year cyclic dynamics expressed in dummy variable (0, 1) matrices ${{\varvec{X}}}_{1}$ (1965–1994) and ${{\varvec{X}}}_{2}$, (1995–2017) in which the number one enables regular effects by three-year intervals (details in Supplementary Methods). ${b}_{t}$ is a parameter for a temporal trend. ${\epsilon }_{z\left(t,j\right)}$ controls for normally distributed environmental disturbances. Additive (to ${\widehat{z}}_{\left(t,1\right)}$) scaling parameters for populations 2 and 3 are denoted by ${\delta }_{2}$ and ${\delta }_{3}$; similarly, climate effects for populations 2 and 3 are quantified with ${\theta }_{k,2}$ and ${\theta }_{k,3}$.

We constructed a logit model, Eq. (2), to generalize the variation of juvenile proportions during the period 1967–2017, based on the number of juveniles per adult female shot by Danish hunters:

2 $$ \begin{array}{*{20}c} {{\text{logit }}p_{\left( t \right)} = a_{p} + b_{L} z_{{\left( {t - 1,1} \right)}} + b_{C} C_{{\left( {t - 3} \right)}} + \mathop \sum \limits_{k = 1}^{2} b_{{\left( {S,p} \right)k}} S_{{\left( {t,k} \right)}} + r_{p\left( t \right)} } \\ \end{array} $$

Here, ${p}_{(t)}$ is the expected proportion of juveniles (in autumn and winter). ${a}_{p}$ is the intercept. The parameter ${b}_{L}$ controls for the responses of ${p}_{(t)}$ to estimated (log) lemming population size in the previous year based on the longest series of lemmings (Kara Sea, ${z}_{\left(t-1,j=1\right)}$) from Eq. (1). ${b}_{C}$ parameterizes the variation related to winter climate ${C}_{\left(t-3\right)}$ (NAOI _Dec–Feb_) three years before. The terms ${{b}_{\left(S,p\right)k}S}_{\left(t,k\right)}$ quantify the effects of precipitation $\left(k=1\right)$ and temperature $\left(k=2\right)$ on the expected juvenile proportions in North-western Siberia in May and June, and these were weighted, *e.g.* for precipitation $\left(P\right),$ as ${S}_{t,1}=\left(1-{w}_{\mathrm{June},1}\right){P}_{\mathrm{May}}+{w}_{\mathrm{June},1}{P}_{\mathrm{June}}$. The weight parameter, ${w}_{\mathrm{June},1}$, is beta-distributed varying between zero and one (uninformative prior beta-distribution, $\mathrm{Beta}(1, 1)$ ^74^; Supplementary Methods) ${r}_{p\left(t\right)}$ controls for random effects (full description in Supplementary Methods).

A log scale state-space model, Eq. (3a), quantifies fertilizer effect on nutrient pools measured in the Danish Straits $\left(j=1\right)$ and the Baltic Proper $\left(j=2\right)$, returning state estimates ${D}_{\left(t,k\right)}$ for DIN $\left(k=1\right)$ and DIP $\left(k=2\right)$ for the southern Baltic Sea in 1970–2016:

3a $$ \begin{array}{*{20}c} {D_{{\left( {t,k} \right)}} = a_{k} + c_{k} D_{{\left( {t - 1,k} \right)}} + b_{k} F_{{\left( {t - lag} \right)}} + {\text{s}}\left( F \right)_{t - lag,k} + e_{{\left( {t,k} \right)}} } \\ \end{array} $$

3b $$ D_{{{\text{obs}}\left( {t,j,k} \right)}} \sim {\text{normal}}\left( {D_{{\left( {t,k} \right)}} + a_{j,k} ,s_{{D\left( {j,k} \right)}}^{2} } \right) $$

where ${a}_{k}$ parameters are intercepts for DIN and DIP, and ${c}_{k}$ controls for serial correlation. ${b}_{k}$ is a parameter for fertilizer-use $({F}_{t})$ effect on the nutrient pools. Non-linear effects are specified with a spline function for fertilizers generating two additive smoothing variables^75^: ${\mathrm{s}\left(F\right)}_{t,k}$. The $lag$ parameter is binomially distributed and can have values of 0, 1, 2 or 3 (years) (Supplementary Methods). ${e}_{\left(t,k\right)}$ is a normally distributed random effect term (Eq. (3a)). Observed nutrient amounts link to the states via an observation model, Eq. (3b), with intercepts ${a}_{j,k}$ and standard deviations ${s}_{D\left(j,k\right)}$.

In the following system, the state-space model, Eq. (4), expressing log-scale long-tailed duck dynamics ${n}_{\left(t\right)}$ (1968–2014) is written briefly as:

4 $$ \begin{aligned} \hat{n}_{\left( t \right)} = & a + cn_{{\left( {t - 1} \right)}} + \beta_{R} \log R_{{\left( {t - 1} \right)}} + \\ & \beta_{D} \left[ {\left( {1 - w_{D} } \right)DIN_{{\left( {t - 1} \right)}} + w_{D} \left( { - 1*DIP_{{\left( {t - 1} \right)}} } \right)} \right] + \varepsilon_{\left( t \right)} \\ \end{aligned} $$

in which ${\widehat{n}}_{\left(t\right)}$ is the expected population size in year $t$. ${n}_{\left(t-1\right)}$ is the population size estimate of long-tailed ducks in year $t-1$ that has a negative binomial error structure^50,76^ around the expected population size ${\widehat{n}}_{\left(t-1\right)}.$ $a$ is the intercept, and $c$ is a density-dependent parameter whose value can vary from zero to one. ${R}_{(t-1)}={1+p}_{\left(t-1\right)}$, adjusted by the parameter ${\beta }_{R}$, is a variable for recruitment based on estimated juvenile proportion (${p}_{\left(t-1\right)}$) in autumn and winter preceding spring migration from Eq. (2). Dissolved nitrogen ${DIN}_{\left(t-1\right)}$ and phosphorus ${DIP}_{\left(t-1\right)}$ describe annual nutrient pools in the southern Baltic Sea (outputs from Eq. (3a)). The nutrient series implemented in Eq. (4) were scaled to have a mean of zero and variance of one and modelled as fixed environmental variables. ${w}_{D}$ weights nutrient effects. Parameters ${\beta }_{R}$ and $w_{D}$ are beta-distributed varying between zero and one (uninformative prior beta-distribution, $\mathrm{Beta}(1, 1)$^74^). The parameter ${\beta }_{D}$ is normally distributed. ${\varepsilon }_{(t)}$ controls for random environmental disturbances (Supplementary Methods). The observation model for Eq. (4) is specified with Gaussian errors and a priori assumed environmental disturbances (wind) as: ${y}_{\left(t\right)}|{n}_{\left(t\right)} \sim \mathrm{normal}\left({n}_{\left(t\right)}+\gamma {x}_{\left(t\right)},{\tau }^{2}\right)$, where $\gamma$ is a parameter for east–west aspect winds ${x}_{\left(t\right)}$, and τ is the standard deviation of a random observation-error process (Supplementary Methods). The joint-likelihood^73^ of the integrated hierarchical model combining Eqs. (1), (2) and (4) is summarized with Supplementary Table S1.

To estimate how biomass of mussels increases with increasing nutrient availability, we built a model based on two mussel biomass surveys of the Wadden Sea, assuming that temperature and nutrients have comparable effects on mussel population growth in the Wadden Sea and the southern Baltic Sea (Supplementary Methods). These effects exclude hypoxia due to nutrient overflow because this does not occur in the strongly tidal well-mixed Wadden Sea, though it does in the Baltic Sea. Thus, a state-space model for mussel (log) biomasses in The Danish Wadden Sea ($j=1$) for the period 1986–1997 is expressed as:

5 $$ \mu_{{\left( {t,j = 1} \right)}} = a_{\mu } + c_{\mu } \mu_{{\left( {t - 1,1} \right)}} + b_{F} F_{{\left( {t - lag_{\mu } } \right)}} + {\text{s}}\left( F \right)_{{t - lag_{\mu } }} + f\left( T \right) + e_{\mu \left( t \right)} + \delta_{{\mu \left( {t,1} \right)}} $$

where ${a}_{\mu }$ is the intercept and ${c}_{\mu }$ is a density-dependent parameter with the term ${{b}_{F}F}_{\left(t-{lag}_{\mu }\right)}$ controlling for fertilizer effect on biomass estimates ${\mu }_{\left(t,j=1\right)}$. ${\mathrm{s}\left(F\right)}_{t-{lag}_{\mu }}$ is an additive smoothing variable^75^ as explained for Eq. (3a). The parameter ${lag}_{\mu }$ can have values 1, 2 or 3 (*cf*. Eq. (3a)). The function $f\left(T\right)$ parameterizes the weighted effects of mean winter temperature (December–February) on biomass estimates two and three years later. ${e}_{\mu \left(t\right)}$ controls for state-process errors due to environmental variance and ${\delta }_{\mu \left(t,1\right)}$ controls for demographic variance ^77,78^. Biomass estimates ${\mu }_{\left(t,j=2\right)}$ for Schleswig–Holstein follows the parameterization given in Eq. (5), excepting an independent demographic error term ${\delta }_{\mu \left(t,2\right)}$ and additive scaling parameter for an observation model (Supplementary Methods).

A logit model for flesh proportion in mussels ${f}_{\left(t\right)}$ for the period 1998–2013 was expressed as:

6 $$ \begin{array}{*{20}c} {{\text{logit}}} \\ \end{array} f_{\left( t \right)} = a_{f} + \mathop \sum \limits_{k = 1}^{2} b_{T,k} T_{k\left( t \right)} + b_{\left( f \right)F} F_{{\left( {t - lag_{f} } \right)}} + e_{f\left( t \right)} $$

in which the (logit) expectations ${f}_{\left(t\right)}$ respond to the intercept ${a}_{f}$ and temporally due to parameters ${b}_{T,k}$ for winter ${T}_{k=1\left(t\right)}$ and spring ${T}_{k=2\left(t\right)}$ temperatures and ${b}_{\left(f\right)F}$ for fertilizer use ${F}_{\left(t-{lag}_{f}\right)}$. The parameter ${lag}_{f}$ can have values of 0, 1 or 2 (years, *cf*. Eq. (3a)). Estimates ${e}_{f\left(t\right)}$ control for effects of random disturbances on flesh proportions (Supplementary Methods).

## Results

The magnitude of lemming population peaks in the western Taimyr Peninsula have declined over time (Fig. 2) and the 3-year cyclic regularity was broken after 1994 (Supplementary Table S1a, Supplementary Methods). Equation (1) shows that lemming populations declined with increasing autumn precipitation in the previous year and increased weakly when early summer precipitation was high (Supplementary Table S1a). Autumn precipitation particularly affected the Kara Sea population. The climate variable effects appeared relatively strong (high *p*-values, *i.e.* Bayesian probabilities; Supplementary Table S1a), with climate variation explaining 29% of the total lemming population variance. Of these climate variables, autumn precipitation had the stronger effect, explaining 84% of the lemming population variation due to climate.

**Figure 3:**
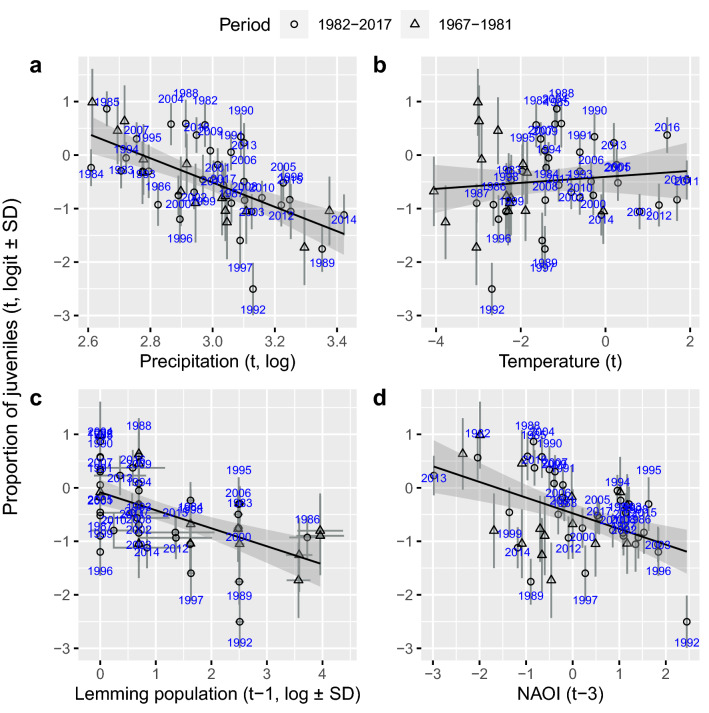
Juvenile proportion of long-tailed ducks from Danish hunting returns in relation to North-western Siberian weather in May and June: (**a**) precipitation and (**b**) temperature, (**c**) estimated lemming abundance in the Western Taimyr Peninsula in the previous year, and (**d**) North Atlantic Oscillation Index (NAOI) three years before. Numbers indicate the year (1982–2017) of the observed data point and circles show the corresponding estimates based on Eq. (2). Triangles show estimates for the period 1967–1981 from which no observations were available (the proportion of juveniles as conditioned on all other data and parameters in the Bayesian model).

Colder and wetter springs and summers in North-western Siberia were followed by fewer long-tailed duck recruits on the wintering grounds in the Baltic. The precipitation variable (Fig. 3a), with weighting particularly for May over June (Supplementary Table S1b), had a stronger effect than the temperature variable (Fig. 3b). Together the North-west Siberian climate variables explained 26% of the total variation in juvenile proportions. The proportion of juveniles declined the year following lemming peaks (Fig. 3c), implying that lemming population troughs that followed high abundance peaks were poor years for long-tailed duck recruitment. Lemming population variation explained 9% of juvenile proportions (Supplementary Table S1b). High NAOI was followed by declines in juvenile proportion with a three-year lag (Fig. 3d), suggesting poor recruitment three years after a warm winter. Together, the climate parameters (Fig. 3) explained 39% of the total variance estimated for the long-tailed duck juvenile proportion (Supplementary Table S1b).

**Figure 4:**
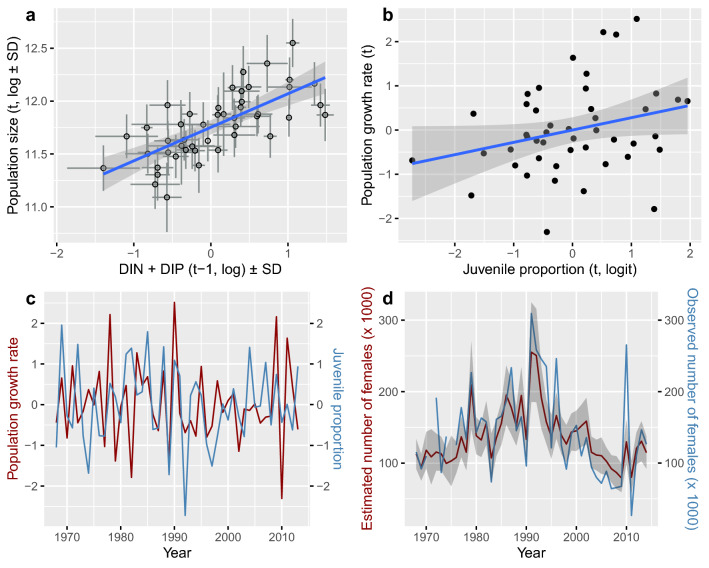
(**a**) Joint effect of (weighted) DIN and DIP (dissolved inorganic nitrogen and phosphorus) in the previous year on long-tailed duck state population size from spring migration counts at the Söderskär observatory. Nutrient amounts were measured in the southern Baltic Sea and their joint effect was calculated with the formulation ${\left({1-w}_{D}\right)DIN}_{\left(t-1\right)}+{w}_{D}\left({-1*DIP}_{\left(t-1\right)}\right)$ designed for estimation with Eq. (4). (**b**) The effect of juvenile proportions estimated using Eq. (2) based on wing data from Danish hunting returns on the population growth rate (*t*) = ${n}_{\left(t+1\right)}-{n}_{\left(t\right)}$ of long-tailed ducks estimated using Eq. (4), and (**c**) the trajectories of these during 1968–2013. Values are scaled to mean zero and unit variance. (**d**) Observed and predicted numbers of spring migrating female long-tailed ducks at Söderskär. Population estimates and confidence interval are calculated using a hierarchical state-space model as given in Eq. (4).

Long-tailed duck spring migration counts as estimated with Eq. (4) increased with increasing DIN and decreasing DIP in the southern Baltic Sea in the previous year (24% variance partition proportion for the joint effect with high statistical confidence, Supplementary Table S1c). The weighting parameter ${w}_{D}$ (= 0.49) suggested similar effect sizes for DIN and DIP. Figure 4a shows the joint effect. Increases in juvenile proportions in autumn and winter, *i.e.* ${R}_{(t)}$ from Eq. (2), were followed by slight increases in numbers of spring-migrating long-tailed ducks ${n}_{\left(t+1\right)}$ from Eq. (4) (5% variance partition proportion, Supplementary Table S1c). This trend is best illustrated by plotting annual population growth rates, ${n}_{\left(t+1\right)}-{n}_{\left(t\right)}$, against ${R}_{(t)}$, although this relationship is noisy (Fig. 4b,c) with parameter distribution skewed rightward: *P*(${\beta }_{R}$> 0.05) = 0.9 (mean of ${\beta }_{R}$ = 0.25 ± 0.17 *SD*). Density-dependent population regulation of the long-tailed duck was observed (22% variance partition proportion).

**Figure 5:**
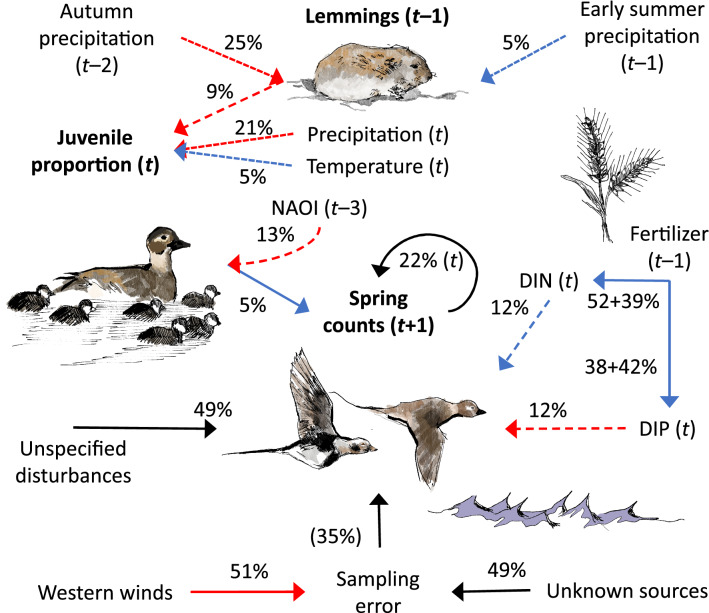
Summary of model parameters affecting long-tailed duck (LtD) *Clangula hyemalis* population size (Spring counts). Dissolved inorganic nitrogen (DIN) and dissolved inorganic phosphorus (DIP) in the southern Baltic Sea are affected by fertilizer use in the Danish farmland. Climate variables affect lemming *Lemmus sibiricus* and *Dicrostonyx torquatus* abundances and juvenile proportions of LtD. “Precipitation” and “Temperature” refer to North-western Siberia in June and July, and “NAOI” is the North Atlantic Oscillation Index. Precipitation in the Western Taimyr Peninsula affects lemmings. Red/blue arrows indicate a negative/positive effect. Solid lines indicate direct effects. Indirect effects (long dash lines) involve trophic cascade forcing from one trophic level to another. Mechanisms behind Siberian climate effects (short dash lines) on lemmings and juvenile proportions were out of the scope of this study and were not included in the general discussion. Black arrows denote random errors from unknown sources and circle arrow illustrates density dependence. Percentages for DIN and DIP describe direct fertilizer plus smoother effects based on the fertilizer series. Variance partition proportions (%) shown for the predictors. The sampling error proportion of the total LtD system (including sampling error variance) is in parentheses. Long-tailed ducks (female and hatchlings, and male and female on the sea), lemming *L. sibiricus*, and fertilizer-use symbol (crop) drawings by Kati Rintala.

The observation process variation (*i.e.*, sampling error or observation error) of spring migration counts was affected by east–west aspect winds such that observed counts decreased with strengthening west winds during the annual census period. The details of wind effects on the observations, which delineated the performance of the population model given in Eq. (4), are reported in Supplementary Methods. After correcting for measurement errors, the number of spring migrant long-tailed ducks showed relatively smooth annual variation and increased from the end of the 1960s up to 1991 followed by a steep decline from 1992 until the end of the 2000s. Some recovery took place in the beginning of the 2010s (Fig. 4d). The whole system with respect to the long-tailed duck is summarized in Fig. 5.

Application of more fertilizer to Danish farmland led to higher DIN and DIP in the southern Baltic Sea the following year. This 1-year lagged response (*lag* = 1) resulted from 94.25% of the simulation updates (Supplementary Table S2).

**Figure 6:**
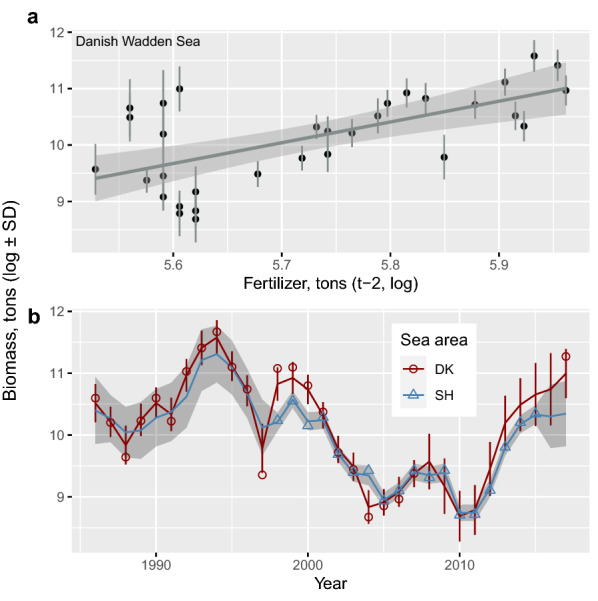
(**a**) Effect of fertilizer application to the Danish (DK) farmland on mussel biomass on the DK Wadden Sea two years later. (**b**) Estimated (lines) and observed (symbols) biomass trends of blue mussel populations in the DK and Schleswig–Holstein (SH) Wadden Sea in 1986–2017.

**Figure 7:**
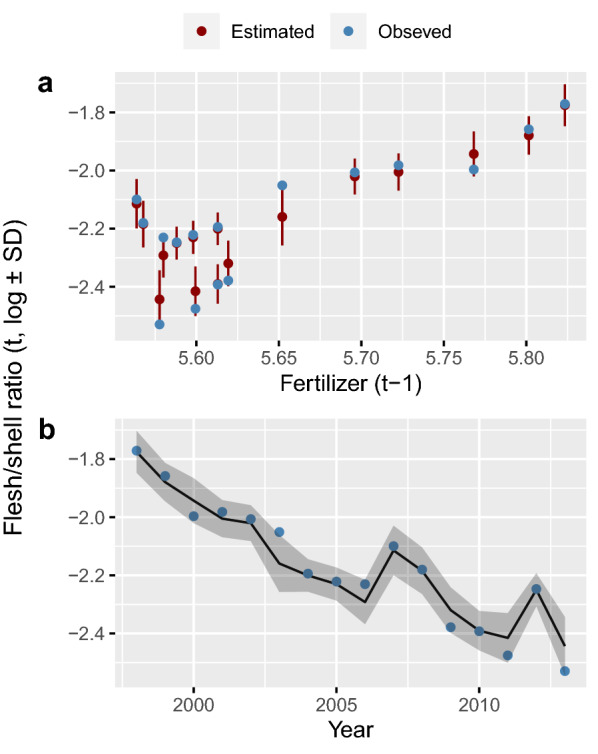
Estimated and observed mussel quality (increasing Flesh/shell ratio means improving quality) based on samples from the Baltic and Wadden Sea and modelling with Eq. (6). (**a**) Flesh/Shell ratio plotted against fertilizer use in the Danish farmland in the previous year. (**b**) Trajectory of Flesh/shell ratio during the period 1998–2013.

**Figure 8:**
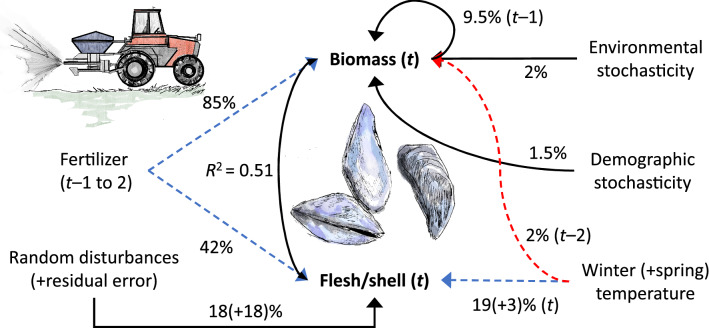
Summary of the models for the blue mussel *Mytilus edulis* biomasses in the Wadden Sea and flesh/shell ratios in the Wadden and Baltic Sea. Red/blue arrow indicates a negative/positive effect of a predictor on a response variable. Fertilizers are applied to Danish farmland while temperature refers to conditions on the Wadden Sea coastline. Dashed lines indicate indirect effects. Solid black arrows denote stochastic effects from unknown sources and circle arrow illustrates density dependence. Numbers represent the per cent of the total response variance explained by a predictor. *R*^2^ comes from regressing log mussel biomass in the Wadden Sea against logit flesh proportion on data the from the Wadden and Baltic Sea (*regression coefficient*: 1.84, *F*_(1, 14)_ = 14.4, *P* = 0.002). Blue mussels and fertilizer-spreading-tractor drawings by Kati Rintala.

High winter temperature was followed by lower mussel stocks in the Wadden Sea 2–3 years later (more weight for 2-year lag). Mussel biomass increased with the increasing use of fertilizers (Fig. 6a) in previous years, most strongly 1–2 years before. Simulation chains revealed frequencies for the lags of 0, 1, 2, and 3 years of 6.3, 31.0, 43.4, and 19.3 percent, respectively. Fertilizer and the smoother based on the spline function dominated the estimated variance for mussel biomass (Supplementary Table S3a). More demographic variance caused by local disturbances affecting mussel dynamics was found for the population in the Danish than the Schleswig–Holstein part of the Wadden Sea (Supplementary Table S3a). Population trends were not perfectly parallel in the two areas (Fig. 6b). Together, mussel biomass showed large variation during the period 1986–1998, entering steep declines from the turn of the 1990s and recovering from 2011 up to 2017 (Fig. 6b). The quality of blue mussels in autumn in terms of (log) flesh/shell ratio of the total biomass, *i.e.* $${\begin{array}{c}logit\end{array}f}_{p\left(t\right)}$$ from Eq. (6), increased with the previous winter’s temperature (19% variance partition proportion, Supplementary Table S3b) and fertiliser use over the two previous years (42% variance partition proportion, Supplementary Table S3b, Fig. 7a,b) but spring temperatures had no effect on mussel quality. These results suggest that mussels in the Wadden Sea were in better condition, as measured in autumn, during the years of high nutrient availability and after warm winters. The system with respect to blue mussel flesh/shell ratio and biomass is summarized in Fig. 8.

## Discussion

This integrated hierarchical model for the long-tailed duck population investigates a complex stochastic system affected dynamically by sub-systems that are also stochastic and regulated by independent variables^73,79^. This model allowed us first, to correct for population estimate errors due to variation in wind velocity and direction at Söderskär, Gulf of Finland, during spring migration, which accounted for 51% of the total observation error variance. This result underlines that ignoring wind effects on counts would have led to serious misspecifications in the state-space model for long-tailed ducks.

This model allowed us to identify and quantify the variables contributing to population size variation of the long-tailed duck by using sub-models for trophic processes driven by nutrient and climate factors occurring at both the breeding and the wintering grounds. The model attributes 51% of total variation in long-tailed duck population size, estimated from spring migration counts, to ecological processes occurring at the breeding or overwintering grounds (Fig. 5). The processes influencing long-tailed duck populations in these two habitats hypothetically are driven by different ecological interactions, for example predator-driven on the breeding grounds^12,14,80^ and resource-driven on the wintering grounds^45,58,70,71^. For example, precipitation evidently influences lemming dynamics on the breeding grounds while temperature and nutrient runoff from the land are thought to affect mussel reproduction, growth and survival on the wintering grounds.

On the breeding grounds, lemming population cycles were related to the number of new recruits of long-tailed ducks, estimated from the proportion of juvenile birds killed by hunters in the winter. The year following high lemming years had few recruits while those following low lemming years had many recruits (Fig. 3c). This is as expected if predator numbers are high the year after a lemming peak and if these predators switch to eggs and nestlings when lemmings are rare^12,16,81^. Western Taimyr lemming dynamics was associated with, and presumably driven by climate, particularly autumn and winter precipitation^38^ (Fig. 5). High autumn precipitation was associated with low lemming population size the next year. Heavy autumn precipitation may cause ice formation at the soil surface in the subnivean space, reducing the insulation properties of snow^39,40^, which leads to poor lemming winter success^22,38,82–84^.

High late spring and early summer precipitation with low temperatures in North-western Siberia were related to decreases in juvenile proportions in long-tailed ducks (Fig. 5). This may be linked to varying nesting areas and predation pressure during snow melt: more precipitation during cold springs delays snow melt^85,86^, which limits open areas for nesting and thus enhances predation on birds’ nests during early breeding season^86^.

Recruitment, as estimated from the juvenile proportion of hunters’ returns, explained 5% of variation in spring migration numbers. This weak association is as expected for a long-lived species like the long-tailed duck^87^, where adult survival drives population dynamics more than does fecundity^88–91^. Furthermore, the juvenile proportion of hunters’ returns is probably representative of the population. Long-tailed duck juveniles resemble mature females in autumn and winter so hunting pressure is unlikely to be age dependent, as for some other quarry species^92^.

On the wintering grounds long-tailed ducks feed mainly on blue mussels^43,44,68^. Mussels reproduce more during cold than warm winters^45,68^. NAOI values that represent variation in winter temperatures showed the strongest association with juvenile long-tailed duck proportion at the wintering grounds in the southern Baltic Sea three years later. If mussels require two or three years to reach the preferred size for long-tailed duck food items^44,72^, then food on the wintering grounds will be scarce two or three years after a warm winter but abundant two or three years after a cold winter. In the Wadden Sea the best-fit lag between cold winters and mussel biomass increase was two years (Fig. 8). Years of high food abundance on the wintering grounds should enhance female condition, increasing their fecundity and maternal investment^45,70^. Thus, we would expect, as we find, higher nesting and fledging success three years after cold winters.

We detected apparent consequences of fertilizer runoff on long-tailed duck populations via the bottom-up forcing primed by dissolved nutrients^47^. Long-tailed duck populations increased with increasing DIN that, through increasing phytoplankton abundance, likely translated into increased biomass and quality of mussels. However, the opposite trend was observed for DIP because increased DIP led to larger and more long-lasting hypoxia and bottom death^46,47,93^. Similar results have been seen in related studies of eiders and other waterbirds^45,58^.

Here we estimated the bottom-up forcing of dissolved nutrients, in the form of nutrient load from Danish farmland, on mussel biomass data from the Wadden Sea. Though we have argued that the predictions about how DIN influences food supply for long-tailed ducks are likely to be appropriate, the results on the Wadden Sea mussels related to DIP cannot be directly generalized to the Baltic Sea. This is because, unlike the Wadden Sea, the Baltic Sea experiences hypoxia due to nutrient overload affecting the cycling of phosphorus^46,47,93^. Hypoxia in the Baltic Sea has resulted in the elimination of benthic fauna over large areas and has severely disrupted food webs^58,94–97^ with expectable adverse effects on sea birds^6,58,98,99^. It is known that hypoxia increases with increasing DIP in the Baltic Sea^47,93^, which accords with the apparent decline in long-tailed ducks abundance with increasing DIP.

In conclusion, we used hierarchical Bayesian models to quantify the effects of a complex array of processes on the abundance of the long-tailed duck, an Arctic migratory bird. This approach allowed us to estimate the amount of variance accounted for by environmental conditions on both the breeding and wintering grounds. Considering drivers at the Arctic breeding grounds, our analysis emphasizes the hypothesis that predation limits nesting success when lemming predators shift to alternative prey such as the long-tailed duck. In the marine winter quarters fertilizer runoff from agriculture apparently drives a bottom-up effect, stimulating food availability by increasing primary production and thereby mussel biomass and quality. However, phosphorus overload could limit food availability by inducing hypoxia and bottom death. These conditions at the wintering grounds affect juvenile survival and adult female body condition before spring migration to the Arctic breeding grounds. The originality of this study resides in our ability to quantify the effects at both breeding and wintering areas that present very different ecological and environmental conditions and challenges. This approach should be useful for analysing the dynamics of other migratory species confronted with divergent environmental conditions and trophic interactions in varying habitats.

## Supplementary Information

Supplementary Information.

## Data Availability

The data supporting the results of this study are provided as Supplementary Data (*R* workspace list-type objects printed as “data_*.docx” and “inits_*.docx” files) referenced in the Bayesian analyses (section “Program codes” in Supplementary Methods).
