# Supplementary material for: Large-scale changes in marine and terrestrial environments drive the population dynamics of long-tailed ducks breeding in Siberia
Source: Sci Rep. 2022 Jul 19;12:12355. doi: 10.1038/s41598-022-16166-7 (PMC9296647; doi:10.1038/s41598-022-16166-7)
Supplement: Supplementary file 1 — Supplementary Information. [file 41598_2022_16166_MOESM1_ESM.zip › data_din.docx]

$N_din

[1] 47

$din

, , 1

[,1] [,2]

[1,] 1.791759 5.905362

[2,] 2.564949 5.739793

[3,] 2.772589 5.988961

[4,] 2.197225 5.883322

[5,] 2.995732 6.327937

[6,] 2.772589 6.234411

[7,] 2.890372 6.415097

[8,] 3.044522 6.287859

[9,] 3.332205 6.514713

[10,] 2.564949 6.390241

[11,] 3.218876 6.338594

[12,] 3.401197 6.565265

[13,] 3.258097 6.369901

[14,] 3.178054 6.436150

[15,] 3.258097 6.369901

[16,] 2.708050 6.487684

[17,] 3.044522 6.655440

[18,] 3.178054 6.684612

[19,] 3.218876 6.375025

[20,] 3.135494 6.338594

[21,] 3.761200 6.739337

[22,] 3.295837 6.779922

[23,] 2.833213 6.617403

[24,] 3.258097 6.434547

[25,] 3.295837 6.711740

[26,] 3.044522 6.769642

[27,] 2.944439 6.675823

[28,] 3.135494 6.650279

[29,] 3.218876 6.682109

[30,] 3.178054 6.694562

[31,] 3.258097 6.487684

[32,] 3.091042 6.350886

[33,] 3.258097 6.347389

[34,] 2.995732 6.393591

[35,] 2.890372 6.349139

[36,] 2.890372 6.340359

[37,] 2.944439 6.308098

[38,] 3.044522 6.270988

[39,] 2.708050 6.388561

[40,] 2.564949 6.369901

[41,] 2.772589 6.333280

[42,] 3.258097 6.395262

[43,] 2.890372 6.165418

[44,] 2.995732 6.326149

[45,] 3.091042 6.375025

[46,] 3.091042 6.410175

[47,] 2.890372 6.395262

, , 2

[,1] [,2]

[1,] 1.223775 5.717028

[2,] 1.526056 5.631212

[3,] 1.280934 5.720312

[4,] 1.223775 5.755742

[5,] 2.116256 5.780744

[6,] 1.808289 5.690359

[7,] 1.589235 5.762051

[8,] 1.280934 5.780744

[9,] 1.704748 5.805135

[10,] 1.547563 5.686975

[11,] 1.931521 5.746203

[12,] 1.629241 5.837730

[13,] 1.871802 5.897154

[14,] 2.028148 5.860786

[15,] 1.945910 5.823046

[16,] 1.547563 5.831882

[17,] 1.808289 5.886104

[18,] 1.609438 5.602119

[19,] 1.774952 5.710427

[20,] 1.974081 5.846439

[21,] 2.332144 5.736572

[22,] 2.091864 5.855072

[23,] 1.648659 5.683580

[24,] 1.960095 5.587249

[25,] 1.686399 5.598422

[26,] 1.504077 5.669881

[27,] 1.280934 5.717028

[28,] 1.252763 5.774552

[29,] 1.547563 5.837730

[30,] 1.098612 5.929589

[31,] 1.547563 5.942799

[32,] 1.435085 5.921578

[33,] 1.686399 5.934894

[34,] 1.223775 5.924256

[35,] 1.360977 5.973810

[36,] 1.791759 6.075346

[37,] 1.609438 6.021023

[38,] 1.648659 6.001415

[39,] 1.722767 6.073045

[40,] 1.526056 6.016157

[41,] 2.001480 5.998937

[42,] 1.308333 5.966147

[43,] 1.740466 5.986452

[44,] 1.791759 5.991465

[45,] 1.435085 6.003887

[46,] 1.609438 6.030685

[47,] 1.526056 6.054439

$Z

[,1] [,2] [,3] [,4] [,5]

[1,] 1.5675042007 -0.556152248 -0.2131421644 -0.488642596 0.3550945426

[2,] 1.0661276055 -0.297990217 -0.1246028868 -0.280414488 0.1947824939

[3,] 0.7021881436 -0.126197241 -0.0646707268 -0.144443341 0.0957517670

[4,] 0.4433679118 -0.018418474 -0.0261183842 -0.061522517 0.0392649825

[5,] 0.2644865677 0.042645095 -0.0033431428 -0.016817888 0.0109930824

[6,] 0.1456920012 0.070505105 0.0080514210 0.001241370 -0.0001010946

[7,] 0.0720006811 0.073863322 0.0142471270 0.003926256 -0.0023031053

[8,] 0.0323160470 0.057204634 0.0237650869 0.004267655 -0.0005894087

[9,] 0.1706837772 0.066032628 0.0060609342 -0.001312777 0.0015651174

[10,] 0.0806116325 0.074799069 0.0132080398 0.003939144 -0.0022903514

[11,] 0.0309985916 0.056053609 0.0242738594 0.004363633 -0.0004037334

[12,] 0.0124519807 0.029300651 0.0326290528 0.010439732 0.0069155270

[13,] 0.0049013111 0.010563929 0.0302198914 0.026245565 0.0208933432

[14,] -0.0002643195 0.003936769 0.0203937022 0.046733156 0.0446548634

[15,] 0.0032916142 0.007603838 0.0274613710 0.032388182 0.0264702450

[16,] 0.0013302570 0.005064293 0.0234675732 0.040487977 0.0353443277

[17,] -0.0004856207 0.003846755 0.0200256245 0.047519891 0.0461131698

[18,] -0.0023520129 0.003623077 0.0175926337 0.053233493 0.0607731222

[19,] 0.0004082596 0.004308816 0.0216182991 0.044200349 0.0404765864

[20,] 0.0032916142 0.007603838 0.0274613710 0.032388182 0.0264702450

[21,] 0.0032916142 0.007603838 0.0274613710 0.032388182 0.0264702450

[22,] 0.0232935527 -0.030030841 0.0105022620 0.009695350 0.3028695881

[23,] 0.0293678193 -0.037482327 0.0088122612 0.001029056 0.3272411745

[24,] 0.0709796862 -0.087214295 -0.0033066162 -0.054054984 0.4653939072

[25,] 0.0610861836 -0.075568218 -0.0003807465 -0.041443159 0.4355796062

[26,] 0.0376094385 -0.047504071 0.0064678669 -0.010391520 0.3579193383

[27,] 0.0169170789 -0.022146726 0.0122214033 0.019092147 0.2750413292

[28,] 0.0038409889 -0.005845432 0.0153408620 0.039960257 0.2044672879

[29,] -0.0028372635 0.002184469 0.0161435665 0.052713136 0.1458478633

[30,] -0.0043106078 0.003873067 0.0160500165 0.056464766 0.1167732572

[31,] -0.0043253763 0.004147891 0.0160998773 0.057364673 0.0915809243

[32,] -0.0032147123 0.003781660 0.0168115043 0.055351734 0.0701364631

[33,] 0.0047495390 0.010254095 0.0299920046 0.026790118 0.0213644825

[34,] 0.0067766565 0.014861493 0.0323591906 0.020268676 0.0158376890

[35,] 0.0159468480 0.036525877 0.0311122110 0.007871077 0.0042266023

[36,] 0.0500038654 0.067972486 0.0183144877 0.003833970 -0.0019291161

[37,] 0.0656041527 0.072745088 0.0151859007 0.003893297 -0.0022644913

[38,] 0.0851463140 0.075076916 0.0127355305 0.003921776 -0.0022546945

[39,] 0.1090623991 0.074752218 0.0107138137 0.003445210 -0.0017575042

[40,] 0.2112354216 0.056997584 0.0023151832 -0.007038150 0.0050708456

[41,] 0.0500038654 0.067972486 0.0183144877 0.003833970 -0.0019291161

[42,] 0.0500038654 0.067972486 0.0183144877 0.003833970 -0.0019291161

[43,] 0.0656041527 0.072745088 0.0151859007 0.003893297 -0.0022644913

[44,] 0.0851463140 0.075076916 0.0127355305 0.003921776 -0.0022546945

[45,] 0.0851463140 0.075076916 0.0127355305 0.003921776 -0.0022546945

[46,] 0.1377507567 0.071699163 0.0086379045 0.001876133 -0.0005437859

[47,] 0.1377507567 0.071699163 0.0086379045 0.001876133 -0.0005437859

[48,] 0.0851463140 0.075076916 0.0127355305 0.003921776 -0.0022546945

[49,] 0.0656041527 0.072745088 0.0151859007 0.003893297 -0.0022644913

[50,] 0.0159468480 0.036525877 0.0311122110 0.007871077 0.0042266023

$X

[,1]

[1,] 5.298317

[2,] 5.355477

[3,] 5.409541

[4,] 5.460836

[5,] 5.509627

[6,] 5.556148

[7,] 5.600597

[8,] 5.643158

[9,] 5.544716

[10,] 5.594057

[11,] 5.645238

[12,] 5.689961

[13,] 5.730862

[14,] 5.770157

[15,] 5.742250

[16,] 5.757232

[17,] 5.771989

[18,] 5.788341

[19,] 5.764639

[20,] 5.742250

[21,] 5.742250

[22,] 5.915091

[23,] 5.922996

[24,] 5.961611

[25,] 5.954007

[26,] 5.932402

[27,] 5.905523

[28,] 5.877901

[29,] 5.849495

[30,] 5.832398

[31,] 5.815008

[32,] 5.797309

[33,] 5.731900

[34,] 5.718655

[35,] 5.677833

[36,] 5.620674

[37,] 5.605859

[38,] 5.590821

[39,] 5.575554

[40,] 5.528301

[41,] 5.620674

[42,] 5.620674

[43,] 5.605859

[44,] 5.590821

[45,] 5.590821

[46,] 5.560050

[47,] 5.560050

[48,] 5.590821

[49,] 5.605859

[50,] 5.677833

$len

[1] 50

$n_knots

[1] 5

$din_1

[1] 1.791759 1.223775
