# Supplementary material for: Large-scale changes in marine and terrestrial environments drive the population dynamics of long-tailed ducks breeding in Siberia
Source: Sci Rep. 2022 Jul 19;12:12355. doi: 10.1038/s41598-022-16166-7 (PMC9296647; doi:10.1038/s41598-022-16166-7)
Supplement: Supplementary file 1 — Supplementary Information. [file 41598_2022_16166_MOESM1_ESM.zip › data_fs.docx]

$N_f

[1] 16

$logit_f_obs

[1] -1.771135 -1.858194 -1.996437 -1.981886 -2.006474 -2.051009 -2.194095 -2.221388 -2.230368 -2.099548 -2.180253 -2.378476 -2.392407 -2.475434 -2.246949

[16] -2.529463

$n_f_beta

[1] 3

$fer_fs

[1] 5.832398 5.815008 5.797309 5.731900 5.718655 5.677833 5.620674 5.605859 5.590821 5.575554 5.528301 5.620674 5.620674 5.605859 5.590821 5.590821

[17] 5.560050 5.560050 5.590821 5.605859 5.677833 5.649662 5.635273

$fsT_mx_Wi

[,1] [,2] [,3]

[1,] 5.5402494 6.2119937 8.295211

[2,] 4.8356552 6.2778815 4.560558

[3,] 5.9872024 5.6946229 7.304116

[4,] 6.4163506 4.0833357 5.570489

[5,] 4.1157616 5.6581742 8.591632

[6,] 2.7080813 3.8232927 3.093105

[7,] 6.2640495 3.7049297 6.175501

[8,] 5.5314038 6.3155159 3.380279

[9,] 5.1494651 2.1129785 3.587501

[10,] 8.5263696 8.3676099 6.562857

[11,] 5.4026902 7.1954193 7.515890

[12,] 4.5001094 2.6991927 4.352749

[13,] 3.4891015 -0.1853919 2.167898

[14,] -0.1598473 4.1507672 4.634226

[15,] 7.3872411 5.5638952 2.680208

[16,] 5.0172422 3.2801717 2.590274

$fsT_mn_Wi

[,1] [,2] [,3]

[1,] 1.5117799 1.53855988 3.2577125

[2,] 0.3483759 1.65395489 -0.5552414

[3,] 1.3645802 1.33166500 2.2946240

[4,] 2.3701469 -0.01428745 -0.2270055

[5,] -0.9212143 1.39799689 3.0434790

[6,] -1.0925703 -1.24681968 -3.4000928

[7,] 1.0947095 -0.64998216 0.7825012

[8,] 1.0146720 2.01795993 -1.3637364

[9,] 0.9563529 -2.72670564 -0.8126235

[10,] 4.7073577 3.64363135 2.1538619

[11,] 1.6687139 3.10019222 1.9963880

[12,] 0.4305037 -1.81150284 -0.3645397

[13,] -0.7134649 -4.54330621 -2.5349978

[14,] -5.3420597 0.03777694 -0.3759635

[15,] 3.0619399 1.21043510 -3.2364989

[16,] 0.5374842 -0.83555265 -1.3609760

$fsT_mx_Sp

[,1] [,2] [,3]

[1,] 9.270986 11.69246 18.15411

[2,] 8.888321 13.01486 17.45605

[3,] 8.399048 13.85238 18.88407

[4,] 6.263392 10.94260 18.01649

[5,] 9.535096 12.37510 17.26549

[6,] 9.469485 13.66050 17.11377

[7,] 8.389914 13.89617 15.78001

[8,] 7.473706 13.60160 16.30091

[9,] 5.009978 11.35324 17.26955

[10,] 10.922942 16.06780 17.38125

[11,] 8.072945 12.12429 19.33808

[12,] 8.274051 15.96348 17.56059

[13,] 8.177386 12.98880 13.48640

[14,] 8.413241 16.43958 18.31968

[15,] 11.001381 11.59865 18.11091

[16,] 3.586280 11.58349 15.58030

$fsT_mn_Sp

[,1] [,2] [,3]

[1,] 2.5382695 5.125426 8.806037

[2,] 2.9287153 4.896093 8.119046

[3,] 2.6164514 5.232548 8.967803

[4,] 0.4003378 3.284077 7.905447

[5,] 2.1002756 4.094256 9.055396

[6,] 1.7140744 3.502909 8.294307

[7,] 1.3224065 4.903853 7.281079

[8,] 0.6690817 4.596088 7.418624

[9,] -1.4633231 3.808182 8.402964

[10,] 3.3903723 5.907729 8.656215

[11,] 1.6157719 3.668304 9.005900

[12,] 2.0227989 5.904636 7.996706

[13,] 1.2463301 4.034641 5.839583

[14,] 0.9031045 6.246927 8.123014

[15,] 3.1722212 3.495114 8.569058

[16,] -2.7402189 3.053104 7.559088
