# Supplementary material for: Large-scale changes in marine and terrestrial environments drive the population dynamics of long-tailed ducks breeding in Siberia
Source: Sci Rep. 2022 Jul 19;12:12355. doi: 10.1038/s41598-022-16166-7 (PMC9296647; doi:10.1038/s41598-022-16166-7)
Supplement: Supplementary file 1 — Supplementary Information. [file 41598_2022_16166_MOESM1_ESM.zip › data_ltd.docx]

$N

[1] 47

$N_juv

[1] 51

$logitp_obs

[1] NA NA NA NA NA NA NA NA NA NA NA NA

[13] NA NA NA 0.91629073 -0.38566248 -0.46203546 0.88376754 -0.69314718 -0.82098055 1.05431203 -2.12026354 0.72754861

[25] 0.26236426 -2.99573227 -0.46203546 0.00000000 0.22314355 -1.66073121 -2.20727491 -0.51082562 -0.96758403 -1.02165125 -0.34249031 -0.89159812

[37] -1.17118298 0.94390590 -0.32850407 -0.19845094 0.36464311 -0.69314718 0.33647224 -0.71334989 -0.59783700 -1.23787436 0.07696104 -1.30933332

[49] -0.56651453 0.41739368 -0.54835444

$nao

[1] -0.70 -0.66 -1.99 0.00 -0.60 -2.36 -1.09 -0.46 -0.02 1.17 0.49 1.02 1.17 -1.69 -1.09 -2.04 -0.18 1.03 -0.84 1.41 1.09 -0.97 -0.90 -0.50 -0.21

[26] 2.45 1.21 0.96 1.63 1.84 0.27 1.35 -1.36 0.21 -0.22 1.10 1.83 -0.66 0.53 -0.31 -0.38 1.08 -0.40 0.86 1.14 -0.09 -2.98 -1.18 1.53 -0.82

[51] 0.57

$n_beta_L

[1] 1

$n_bCj

[1] 2

$Tmx_s

[,1] [,2] [,3] [,4] [,5] [,6] [,7] [,8] [,9] [,10] [,11] [,12]

[1,] -27.31666 -19.16747 -24.586338 -17.762634 -7.3257068 2.624150 12.281697 9.821680 6.2664637 -5.665297 -18.556467 -18.04339

[2,] -23.84335 -27.40466 -18.444232 -13.133828 -7.1682523 1.823171 14.438249 10.575283 4.1710010 -6.578576 -21.459465 -20.79001

[3,] -26.34935 -33.03787 -26.346372 -16.605989 -5.9233470 2.057226 11.202357 9.356264 4.5142766 -10.595984 -16.562617 -13.96331

[4,] -26.53846 -24.95504 -15.762285 -9.735955 -5.6312222 4.119435 13.955909 9.910482 4.3613295 -1.059603 -8.912442 -14.82615

[5,] -24.76415 -20.78032 -14.511929 -14.059279 -5.6239358 1.477425 8.801902 8.934223 2.3638768 -7.324263 -25.720993 -27.92565

[6,] -28.94216 -30.95096 -24.173698 -16.681999 -7.6545014 3.376380 13.244264 8.000042 2.5614363 -6.778198 -9.644464 -16.16217

[7,] -26.29442 -25.75654 -18.445955 -16.103198 -7.7036995 1.632099 10.323193 6.544886 4.2882569 -7.398148 -14.914046 -20.35964

[8,] -25.45775 -27.54560 -20.861979 -16.691369 -4.2926748 2.526904 10.169482 12.049726 4.8969485 -7.485354 -11.651656 -17.97332

[9,] -25.86238 -22.02181 -21.609238 -12.000285 -8.1662239 3.843242 9.627089 8.756980 2.3392088 -5.490786 -21.700079 -20.23875

[10,] -26.15889 -19.09700 -17.424188 -12.345926 -5.2330560 3.029094 10.201168 10.831237 3.0431305 -7.047272 -16.010204 -22.18783

[11,] -28.34079 -27.47074 -17.796528 -12.856282 -6.1757864 2.376937 10.119557 9.497201 4.3965605 -9.283833 -23.651807 -16.54718

[12,] -20.94742 -24.01850 -20.334462 -12.654925 -6.0278743 3.823309 11.222467 9.696474 5.7001376 -6.321719 -16.691832 -16.70801

[13,] -22.33553 -24.49061 -18.850965 -9.873726 -5.9063156 3.747563 10.144704 10.287675 4.5299503 -10.693293 -14.020622 -22.18963

[14,] -24.49667 -30.84561 -24.412691 -10.903326 -3.4185101 5.786238 10.947807 8.541507 3.7156269 -11.334638 -11.132450 -20.95673

[15,] -25.73831 -24.84674 -22.130522 -17.278804 -6.1599546 1.922640 11.749747 8.413533 3.7426330 -3.759035 -11.981604 -26.45600

[16,] -30.50696 -34.39144 -25.532138 -16.524655 -5.4908294 4.276006 12.923415 9.361771 4.9389274 -7.317478 -15.251798 -21.54540

[17,] -25.42075 -21.98836 -21.632729 -12.337082 -5.4277509 2.763369 8.746616 8.858712 3.7493543 -4.948647 -20.978695 -18.60729

[18,] -14.34750 -22.67828 -23.970426 -12.646629 -7.3008808 3.945838 10.127769 12.499037 3.8785393 -5.001067 -9.860960 -16.03334

[19,] -26.41128 -25.28063 -23.225028 -10.121395 -4.2985694 4.002715 12.341411 10.163062 3.5239942 -10.676391 -17.011346 -16.36981

[20,] -19.48316 -22.67004 -19.041392 -16.024953 -6.6257939 3.998156 10.956810 10.636522 5.4164443 -3.654249 -12.077445 -18.26774

[21,] -16.29904 -18.49696 -17.803676 -20.111637 -5.3835235 5.015341 13.909263 10.316952 6.0713154 -6.007758 -19.318316 -17.30195

[22,] -26.84480 -26.46253 -18.901326 -14.528654 -6.5787717 6.145313 9.732010 10.095856 5.7440728 -3.284789 -13.681047 -20.67279

[23,] -23.44858 -25.13780 -18.472836 -14.710379 -5.6483076 2.510209 12.604834 8.302325 2.4044469 -3.593626 -9.801024 -25.33621

[24,] -27.91725 -24.87021 -20.491913 -14.888694 -4.5774500 1.102032 13.718741 9.689980 4.5373495 -4.000884 -18.006850 -21.43801

[25,] -20.33033 -24.94931 -18.169433 -19.290211 -3.8857267 4.146403 10.629442 9.629598 4.3512449 -6.464439 -16.044804 -14.25255

[26,] -26.38599 -21.42209 -16.197922 -15.757655 -3.3402934 3.188351 11.499307 9.334600 2.9414769 -5.661499 -17.562559 -20.57910

[27,] -28.47961 -23.20119 -15.874213 -8.422883 -4.0155743 5.912446 14.567529 9.211871 4.0523145 -7.373581 -21.838035 -21.93854

[28,] -23.28540 -26.34588 -22.345003 -11.977263 -3.8127513 4.932362 12.185732 9.025105 7.3571469 -5.362440 -13.564144 -20.90320

[29,] -24.21527 -22.00124 -18.563588 -18.797602 -3.1705833 1.311677 10.795310 9.709624 1.7174347 -11.138175 -19.278821 -16.74545

[30,] -20.26865 -21.31586 -15.462645 -13.791982 -5.0390382 5.768116 11.458013 9.464172 4.7952560 -6.784209 -10.775291 -21.81864

[31,] -25.65293 -31.66697 -15.339430 -12.285623 -6.2408585 5.110884 11.710139 9.302679 3.4202249 -2.670515 -17.495299 -19.87385

[32,] -20.63324 -16.03053 -17.528172 -5.897809 -3.8663147 2.847836 12.139904 12.451350 4.4404302 -4.231638 -15.752256 -21.81042

[33,] -22.72792 -20.12698 -15.390388 -14.149647 -4.6325403 2.434802 12.608852 8.299348 0.6974147 -5.453919 -9.499048 -21.75873

[34,] -26.74750 -26.72253 -16.569819 -7.226273 -2.8890505 2.645289 9.608991 8.667847 6.5908562 -2.109667 -17.415874 -24.75512

[35,] -23.24915 -29.88074 -17.999694 -15.789696 -5.7967086 2.849465 12.616007 11.301930 0.5972947 -11.554831 -21.357332 -22.06976

[36,] -26.62005 -18.64244 -24.470326 -15.264109 -5.0334448 2.634425 11.299336 9.378594 3.9975320 -5.101457 -14.933492 -14.72232

[37,] -25.86213 -21.43690 -16.790893 -11.275324 -2.6651855 4.580953 11.177269 12.355637 3.7178118 -6.284146 -16.341562 -25.88042

[38,] -27.93752 -25.04459 -22.307141 -15.579733 -3.7915371 6.131670 11.716496 12.829834 5.1166230 -5.838538 -11.135646 -16.08342

[39,] -26.48875 -24.14928 -16.921408 -14.389935 -5.4166818 5.609405 11.740778 10.660442 1.8294105 -4.775008 -15.173740 -23.18840

[40,] -23.77240 -26.38545 -20.499015 -12.592985 -2.9772137 6.974718 9.677668 13.210676 5.2181213 -5.711720 -18.832416 -17.79619

[41,] -24.44253 -26.18075 -23.007303 -15.803436 -5.3350925 5.018918 12.076497 8.255630 4.0827517 -5.223844 -12.687140 -20.93221

[42,] -18.93426 -20.95154 -21.851895 -14.228820 -2.2907909 5.245527 11.172132 9.424426 7.1335238 -2.898021 -11.183724 -17.24760

[43,] -24.84095 -21.60452 -19.836420 -16.972196 -4.4183293 5.474359 13.868478 9.315922 4.1190027 -7.678891 -15.513815 -17.02478

[44,] -16.04702 -29.00842 -16.709140 -6.775891 -5.6342923 3.611434 12.465485 9.395067 5.5928888 -1.418448 -13.664476 -18.61072

[45,] -18.02964 -20.99636 -19.574592 -12.757281 -3.9776210 3.874729 11.320068 11.182570 6.6831560 -2.671285 -12.379027 -15.97735

[46,] -19.58210 -27.14552 -20.958503 -11.324246 -5.7420282 4.437135 13.194178 11.044093 6.5215971 -2.447030 -14.781766 -23.97754

[47,] -21.24319 -28.85192 -17.743075 -10.263205 -3.5057319 4.431164 9.771878 9.635083 3.2118464 -2.048842 -13.204206 -22.91578

[48,] -20.30986 -23.05145 -12.382103 -7.779161 -0.8431148 7.504064 9.974096 8.886307 6.5852875 -1.701137 -12.593446 -11.86759

[49,] -18.32680 -15.05536 -17.289847 -9.539156 -3.3097232 7.728656 13.930282 9.847823 7.2930141 -3.232675 -12.917408 -17.00756

[50,] -25.08466 -22.84752 -24.344657 -10.244757 -2.2735265 5.133299 14.651928 11.253970 4.3515642 -4.902209 -11.147645 -18.59031

[51,] -27.91685 -23.80046 -12.501669 -9.818806 -3.0457279 5.086757 10.185352 8.614343 3.6278967 -5.492631 -16.167154 -15.56906

[52,] -24.60413 -22.77794 -16.136790 -7.745376 -1.6946293 7.378347 12.169147 9.155436 5.2592384 -3.944753 -11.608538 -15.71128

[53,] -17.58178 -16.06499 -14.676930 -7.944677 -2.6325331 8.319859 15.318136 11.929583 6.7685802 -1.542677 -15.218505 -20.39068

[54,] -19.17556 -21.29183 -9.346645 -11.587183 -6.1111888 5.178201 12.491338 9.025459 3.8132752 -2.818471 -12.408298 -14.89415

$Tmn_s

[,1] [,2] [,3] [,4] [,5] [,6] [,7] [,8] [,9] [,10] [,11] [,12]

[1,] -35.05607 -26.34332 -33.53132 -26.94673 -14.833555 -2.03878912 4.757467 3.281615 0.92697173 -11.315414 -24.58340 -25.77208

[2,] -32.28932 -33.88967 -27.39391 -22.95908 -14.705584 -2.65417615 7.091961 4.362275 -0.29194196 -11.778290 -27.57041 -29.30880

[3,] -34.62993 -40.39629 -33.50403 -26.30794 -14.059618 -2.58737190 3.602452 3.017692 -0.55858057 -16.111954 -23.79499 -22.05005

[4,] -35.51293 -33.52581 -24.49818 -18.93561 -13.561240 -1.29882118 5.606453 3.175415 -0.45323812 -6.011170 -16.04483 -21.47678

[5,] -31.16729 -27.73221 -23.58631 -22.59844 -12.664353 -3.48470417 3.226389 2.892504 -1.89176441 -12.626907 -32.03656 -34.50347

[6,] -35.24458 -36.88963 -31.73577 -25.84616 -14.196160 -1.22629016 5.069084 2.576043 -1.22528426 -11.702544 -16.30771 -23.54756

[7,] -33.63633 -32.12188 -26.02759 -24.57830 -15.352074 -2.00981289 4.137285 1.828789 0.05547607 -13.691330 -22.77081 -27.35924

[8,] -34.32355 -33.85671 -28.44049 -26.46751 -11.507537 -1.21027544 3.856065 5.385917 0.95027414 -12.940920 -19.71324 -24.89859

[9,] -33.08765 -30.45498 -28.80443 -21.56529 -15.623719 -0.60711607 3.737156 3.370956 -1.38512391 -10.246823 -27.59267 -29.03389

[10,] -34.23218 -26.98183 -26.92057 -21.29590 -11.865727 -1.16453556 4.027331 4.259269 -1.17243928 -13.567058 -23.29050 -29.59990

[11,] -34.32112 -34.48280 -26.87586 -22.22972 -12.804848 -1.81230152 4.008292 3.375714 -0.36326050 -15.979393 -30.59152 -24.16768

[12,] -29.31627 -31.47615 -28.85140 -21.60268 -12.299284 -0.26323385 5.055111 4.387793 1.50287549 -11.700444 -23.67631 -23.20169

[13,] -29.65814 -31.84103 -27.84064 -18.08484 -12.112406 -0.16731752 4.499101 4.152350 0.10632857 -16.376579 -22.34205 -29.92121

[14,] -31.90588 -38.13204 -31.93971 -19.56836 -9.730656 0.85002944 5.236293 3.390399 -0.33218654 -17.064720 -18.68876 -27.98244

[15,] -32.96526 -32.32942 -29.69845 -26.36126 -12.373904 -1.21860034 5.018450 3.500482 0.05243510 -8.234814 -18.41308 -32.90721

[16,] -37.57465 -40.75874 -33.70618 -25.13521 -12.571521 -0.55058413 5.717140 4.129349 1.10749840 -12.164603 -23.64137 -28.82332

[17,] -33.04349 -29.83517 -28.70985 -20.09772 -11.167428 -0.95703573 3.659044 3.405760 -0.01883299 -9.327439 -26.58844 -25.59120

[18,] -23.56187 -30.13459 -30.82534 -21.23404 -14.072200 -0.59921599 3.968712 5.906046 -0.50081521 -10.450178 -17.19065 -24.55409

[19,] -33.78851 -31.99581 -30.83724 -18.06246 -10.797991 -1.16679254 5.213770 4.100010 -0.59263493 -16.528228 -23.15551 -23.25298

[20,] -26.92330 -29.83688 -25.97407 -25.04370 -13.004870 -0.78881925 4.725915 4.535647 1.30878353 -8.260159 -18.67111 -26.42424

[21,] -23.61026 -27.14137 -26.15012 -29.20689 -12.364240 -0.09059554 6.901913 4.086796 1.57007067 -11.350709 -26.91918 -26.47544

[22,] -34.41129 -34.50231 -26.96252 -23.19839 -13.744554 0.86673383 4.335070 4.527776 1.59871997 -7.245298 -20.02717 -27.15459

[23,] -30.22121 -31.96761 -26.87608 -24.42871 -12.356994 -1.23185912 5.163574 3.448523 -1.33457237 -8.127052 -16.67053 -32.86540

[24,] -35.86802 -32.32170 -30.04324 -23.58239 -10.168816 -2.71135198 5.628045 3.816745 0.26782780 -9.394161 -25.16568 -28.67949

[25,] -28.69797 -31.73030 -26.95922 -27.51887 -10.463096 0.10797315 4.523904 4.091798 -0.01144936 -11.938752 -23.73084 -22.90803

[26,] -32.61007 -28.39636 -25.82732 -24.93778 -9.055142 -1.12263292 4.697248 3.777048 -0.89406243 -10.823276 -25.94121 -27.72575

[27,] -34.28942 -29.51732 -24.55663 -16.29623 -10.531876 0.81613786 6.849291 3.768193 -0.28419401 -12.836872 -27.72840 -30.75311

[28,] -30.89738 -34.82326 -29.32678 -21.04614 -9.857174 0.29437013 5.762004 3.465035 3.15400695 -11.201495 -21.57101 -27.88515

[29,] -31.79007 -29.52985 -26.11763 -27.06443 -9.149670 -3.08401034 3.556435 3.882468 -2.32080581 -17.329138 -26.27284 -25.32394

[30,] -28.34704 -28.57000 -23.50774 -22.66060 -11.577792 0.98659985 4.695093 4.204710 0.66500006 -12.047975 -18.49751 -29.11831

[31,] -32.15233 -37.68669 -22.52729 -20.85584 -12.935469 0.72160112 5.012592 3.656508 -0.83306866 -7.901225 -24.38683 -27.16980

[32,] -28.31369 -23.32682 -25.63059 -14.79838 -10.507547 -1.37866990 5.523411 6.634082 0.41519636 -9.386763 -22.79514 -29.13146

[33,] -30.31760 -27.45047 -23.73696 -23.10926 -11.296602 -1.86360689 5.973214 2.720332 -3.40825874 -10.599152 -16.35462 -29.06681

[34,] -34.44033 -33.20572 -23.39596 -16.51119 -8.640267 -1.22132958 4.195036 3.903850 2.86760145 -5.904450 -24.57623 -32.00717

[35,] -30.51114 -37.26859 -26.46959 -23.92993 -12.492677 -0.52906322 6.117664 5.989017 -2.43151224 -17.163371 -27.56620 -29.01897

[36,] -33.87224 -27.91336 -31.57222 -23.56000 -11.153617 -1.01811651 4.319805 4.033680 -0.01939703 -10.297001 -21.50362 -20.77144

[37,] -32.52814 -27.32523 -23.79308 -20.01341 -9.304427 0.73120868 4.532050 6.315528 0.09794042 -10.814259 -23.27121 -31.12294

[38,] -34.26089 -32.33887 -30.02564 -25.00478 -9.713086 1.64643544 5.450927 5.866693 1.43318707 -10.729600 -18.25002 -22.65184

[39,] -34.00821 -31.47122 -23.64660 -21.87385 -12.400666 1.14508088 5.224804 4.970865 -2.03428156 -10.016450 -22.19175 -30.60051

[40,] -31.42811 -33.52615 -28.80835 -22.09830 -8.806427 1.55580777 4.416618 6.620195 1.27857272 -9.969058 -25.04336 -24.68415

[41,] -30.83831 -32.15703 -29.06868 -23.82910 -11.330443 -0.02199220 6.157023 3.440435 0.32157786 -10.506750 -19.67045 -26.93017

[42,] -25.00711 -26.65022 -28.67344 -22.69936 -8.802249 1.37829476 7.060234 4.699278 2.99538904 -7.137163 -18.66965 -23.07691

[43,] -31.21972 -28.18965 -26.93450 -24.83283 -10.463008 1.18760894 6.426260 4.328852 0.59795913 -12.791860 -22.53386 -23.86188

[44,] -23.39156 -35.49340 -24.25643 -14.30642 -11.386380 0.54068062 7.082008 4.395529 1.47095039 -5.669331 -19.75497 -25.40179

[45,] -25.70849 -29.05510 -27.58462 -21.67205 -10.655161 -0.62640761 5.562699 5.239041 1.76257744 -7.819406 -18.54711 -23.89887

[46,] -26.96566 -33.69997 -28.08780 -20.67152 -11.255802 0.01045664 5.655173 5.308759 2.52580715 -6.073668 -21.49473 -30.29225

[47,] -29.85740 -34.25973 -24.64048 -18.49275 -8.869553 0.12491713 4.643789 4.226459 0.02881012 -6.660233 -20.53918 -29.58948

[48,] -27.46716 -31.18558 -19.89173 -15.65202 -7.526504 2.64434763 5.139315 4.139442 3.34099596 -5.733331 -17.82497 -17.97253

[49,] -26.13843 -22.66485 -24.71873 -17.59658 -8.744213 2.33189157 7.811032 5.285381 3.19875308 -7.519319 -18.97936 -24.09854

[50,] -31.21617 -29.48862 -31.70171 -18.91563 -7.911528 0.73036007 6.997993 5.402327 0.33691522 -9.476479 -17.94243 -24.85040

[51,] -35.11866 -31.10935 -20.17897 -18.15944 -9.206651 0.94504124 4.374803 3.972531 0.47715768 -10.557470 -23.20110 -21.46711

[52,] -32.04088 -28.94642 -22.28464 -15.95150 -8.409940 2.96717163 5.718198 4.149226 1.60745017 -9.047814 -17.63215 -22.20758

[53,] -24.68651 -22.80941 -22.19218 -16.15629 -10.230243 2.74196963 8.430310 5.990074 3.53895087 -5.133456 -20.83483 -27.86937

[54,] -26.44394 -27.43993 -17.49629 -19.43830 -11.877860 0.76590488 6.387232 4.340787 0.01364296 -7.218396 -19.34299 -21.63303

$Pre_s

[,1] [,2] [,3] [,4] [,5] [,6] [,7] [,8] [,9] [,10] [,11] [,12]

[1,] 33.288770 38.047729 26.65114 13.533411 13.99447 28.22741 27.90142 56.50945 44.45008 26.61769 12.800993 19.31436

[2,] 27.172151 21.985142 19.42310 17.567520 19.98743 23.46302 23.83895 39.57465 42.52227 20.78687 12.000984 21.72436

[3,] 28.901407 10.243647 16.42556 16.833089 27.30329 33.33723 50.08098 39.73502 54.05156 26.64649 24.266695 23.95480

[4,] 25.628957 26.626103 26.83726 33.679019 19.53471 26.46772 36.89210 39.74089 44.62790 45.14736 35.343487 25.10423

[5,] 16.638875 20.037503 35.65716 22.747127 18.89491 30.33799 47.85615 57.17848 34.06093 25.65718 19.628529 25.10598

[6,] 18.695279 15.841551 19.28221 17.364871 11.76445 22.63284 48.25390 35.57335 30.39577 16.10899 29.489271 24.38778

[7,] 20.349717 14.439383 19.28349 13.671110 16.63007 24.36671 47.17166 29.14354 31.82362 24.21118 28.835174 26.49691

[8,] 43.872687 23.713654 16.19682 10.907506 18.47579 20.97258 41.08237 60.90019 55.48421 24.41292 39.414952 22.27527

[9,] 23.108438 30.611221 15.05707 26.962610 14.78891 16.37134 42.92819 47.87304 29.95925 33.40529 10.288786 42.95260

[10,] 19.372465 30.091769 23.20089 12.954006 26.65223 40.10340 23.75459 55.93603 40.01690 35.77490 19.275520 16.09604

[11,] 17.036320 23.618179 26.44474 16.323279 27.82589 24.28898 28.52144 32.18634 47.77343 24.38443 8.243328 27.97647

[12,] 27.771530 13.390254 16.29255 22.192321 15.59343 33.01052 31.35891 31.45771 37.57584 32.06658 23.062493 35.10486

[13,] 17.022080 22.019557 17.99724 26.991541 17.80338 36.33852 31.29370 48.09584 39.82177 20.18203 24.010224 18.89959

[14,] 15.788699 9.674517 25.65689 26.536557 22.73111 21.22674 28.57069 23.11301 38.21247 20.03647 30.842142 22.44955

[15,] 18.699687 20.491419 22.10141 10.256809 15.43113 18.52304 36.58056 29.48645 19.44078 26.98622 21.947379 15.97239

[16,] 18.298246 12.218435 11.16429 10.312523 12.36278 38.96465 30.59914 37.50183 31.67642 19.90450 25.180291 24.39927

[17,] 19.426399 23.774641 10.98520 16.143746 17.87311 34.18854 41.55845 54.70787 39.73309 36.83360 13.098796 27.80813

[18,] 60.757315 16.346497 15.10135 19.561961 11.07437 39.83744 19.53772 34.05078 30.21275 31.12319 25.202904 22.11757

[19,] 19.572991 16.334477 15.52531 26.116686 15.27638 45.87148 27.52069 67.05138 40.46120 23.25897 15.617935 25.38706

[20,] 25.410267 26.548240 14.57077 15.370276 12.96278 24.45300 40.47139 42.97787 31.39395 29.83756 33.175791 24.45449

[21,] 24.498621 34.747180 21.36093 11.268222 12.50438 18.06301 33.60744 39.51476 40.30004 17.78787 16.007057 30.99430

[22,] 19.519498 16.021217 23.16288 13.734297 11.49680 30.06729 29.05269 53.73560 40.86371 33.77987 24.161348 14.53984

[23,] 19.286935 17.753813 29.11297 23.415425 14.06777 31.23894 43.48989 42.28306 40.36029 26.99156 32.383950 15.09978

[24,] 26.657610 16.352429 24.93931 14.906061 21.93192 19.40292 24.39080 39.31891 22.48978 39.79236 21.450766 21.25525

[25,] 37.828706 20.967709 26.16489 11.349087 16.13717 28.96809 31.01224 43.73859 50.17000 34.43269 27.863429 43.26404

[26,] 27.739450 27.216080 28.04697 19.191635 26.88053 35.13326 47.44692 41.79015 42.65818 32.40868 27.573292 35.60389

[27,] 8.519226 16.501106 25.48886 23.357731 23.00907 18.84494 20.43746 51.46371 23.68127 25.81443 19.299125 35.16138

[28,] 25.667734 24.196550 10.97870 27.832789 20.64242 23.74605 25.41926 28.47770 37.37507 38.76317 20.159762 21.37149

[29,] 18.753056 17.142909 18.82015 7.505118 22.02358 26.01029 41.30631 42.98464 33.34521 21.54247 22.341051 29.17380

[30,] 37.375957 22.426939 31.85913 23.066101 15.90622 16.50861 25.02433 45.87353 27.67786 25.29124 32.896593 27.94211

[31,] 12.447913 6.970411 20.87172 13.338347 15.34666 14.75547 23.31406 30.86706 38.71084 37.74354 23.034973 16.02514

[32,] 32.880120 20.286778 27.22056 20.362424 15.62024 19.13624 18.37538 44.41047 30.28377 31.45127 28.938566 26.70345

[33,] 21.530972 27.785560 23.60568 11.189080 16.59764 24.20649 43.59353 33.56206 17.05066 26.44639 21.218220 18.24687

[34,] 27.030145 27.182503 20.30600 23.165303 19.85539 30.86230 26.35786 22.36134 28.89507 37.53163 21.132126 21.72535

[35,] 23.438046 11.900754 23.39919 14.293177 24.51179 30.82359 23.41830 49.80211 27.67704 18.41681 9.638827 27.28159

[36,] 11.645980 21.975299 12.28836 15.834601 18.34750 23.84993 40.97013 41.87900 34.29123 37.71779 26.848286 26.12693

[37,] 21.413545 19.326948 18.23278 25.538850 15.00582 33.44364 22.23104 36.22342 43.72328 26.95839 19.334994 10.77651

[38,] 15.316417 14.143659 12.84699 18.693351 19.37594 24.40172 36.39603 30.26275 40.55069 31.82240 27.333253 31.48872

[39,] 16.777211 20.832294 16.94483 20.017825 17.41021 25.15987 44.34577 44.93158 42.06741 29.91429 20.159103 15.48646

[40,] 28.196792 26.389705 29.59909 23.817040 23.85052 19.45296 24.18721 35.00976 36.13841 23.31510 18.598959 25.35833

[41,] 22.792283 19.439082 22.50998 12.380757 15.07593 29.65883 20.61423 39.47291 24.13660 32.34837 26.352459 24.21117

[42,] 21.729276 28.194842 21.19780 21.509967 24.37652 28.27022 38.01413 34.01046 42.76895 36.84842 30.731501 25.65637

[43,] 19.553141 27.258004 18.06221 7.090901 21.16608 26.19481 37.93573 30.70771 58.92356 33.28708 30.187775 34.03819

[44,] 33.489848 7.854222 20.48698 24.398211 15.71484 15.78946 36.90125 44.50091 38.19462 39.92939 29.471012 33.09120

[45,] 43.545422 31.914751 22.77224 20.155775 21.11106 26.68820 14.26477 33.23102 42.21984 35.06903 26.692623 45.52089

[46,] 47.225316 14.990744 19.82033 37.044316 18.07833 27.85933 40.37328 43.69673 52.54292 40.67856 20.624577 19.12986

[47,] 41.963771 9.380812 23.63970 27.700315 20.04548 40.71536 46.69525 48.70206 32.13043 28.70191 32.026151 35.31650

[48,] 28.176805 23.151026 34.23789 22.531613 18.21181 28.42709 35.90922 38.72355 27.02388 44.03614 30.811473 24.52816

[49,] 21.129122 27.704645 18.95156 16.311041 22.94001 33.81545 48.07800 47.65877 31.75197 23.20548 20.594178 26.06159

[50,] 26.396534 29.335787 11.11505 16.317845 21.43184 25.15563 17.03648 26.85946 28.71012 27.29451 27.303880 12.66729

[51,] 13.291993 12.393515 23.40122 24.384554 32.66961 24.60571 36.23917 52.93501 41.99956 18.38774 31.968505 22.61047

[52,] 14.429098 22.816380 32.38797 12.162018 22.92700 38.18712 48.99581 23.60451 38.78917 25.92445 32.472507 39.25904

[53,] 19.439547 30.359396 24.40037 29.534150 18.08630 22.84512 19.91923 48.49692 37.76754 39.71049 20.117107 14.06194

[54,] 36.664299 26.490445 23.09382 12.552015 17.50478 37.45860 17.01631 48.07398 27.74584 32.41174 18.980386 24.94545

$mean_alpha_juv

[1] 6.130633

$mean_bCj

[1] -2.1322490 0.0989914

$mean_beta_L

[1] -0.2302939

$mean_beta_nao3

[1] -0.2945997

$n

[1] 11.65635 11.42858 11.60578 NA 12.16263 11.37247 11.82897 NA 11.73846 12.08756 11.80323 12.33141 11.86954 12.00208 11.96799 11.20730

[17] 11.99020 12.01732 12.13953 12.36096 11.88740 12.01184 11.47158 12.64233 12.46155 12.41086 12.36830 11.70655 12.41397 11.73464 11.42467 11.85712

[33] 11.93641 11.60993 11.81792 11.45611 11.28888 11.18243 11.39193 11.06946 11.09368 11.11971 12.48866 10.19369 11.61492 11.89465 11.74775

$east

[1] 0.413514315 -0.142907769 -0.414847572 -0.456427754 1.540477896 -0.456468638 1.679093328 0.003069765 1.071275326 0.457165106 1.760761319

[12] -1.194722224 0.391775482 0.888112957 -0.283512610 0.527118577 0.733488386 -0.096820919 -0.675374075 0.515291597 0.272221698 -1.204248336

[23] -0.626341013 -0.496131429 -0.856424824 0.235973828 1.530793453 0.528365226 1.389090719 -0.225215789 -0.639804444 -0.196868492 0.660416767

[34] -1.582124823 -1.300284166 -0.252714042 -1.116035013 -0.885679024 -0.514426627 -0.993529324 0.212862483 0.606292945 1.963642779 -3.182098921

[45] -0.278892180 -0.319307018 1.010403073

$s_var

[1] 0.03736048

$n_beta_din

[1] 1

$n_wD

[1] 1

$pool

[,1] [,2]

[1,] NA NA

[2,] NA NA

[3,] NA NA

[4,] -4.098292597 -1.477728482

[5,] -2.717653126 -1.578955161

[6,] -1.694520200 -1.023976569

[7,] -1.551256074 -0.676855146

[8,] -0.486984207 -0.520457951

[9,] -0.435208506 -0.928343051

[10,] -0.082169984 -0.639280565

[11,] -0.421007403 -0.478538959

[12,] 0.097733376 -0.428451183

[13,] -0.171308315 -0.890501569

[14,] -0.006141473 -0.538374611

[15,] 0.415951678 -0.045414375

[16,] 0.287935460 0.229069667

[17,] 0.244010788 0.125867948

[18,] 0.214393966 -0.077350962

[19,] 0.305360928 -0.123982437

[20,] 0.742744359 -0.039842357

[21,] 0.716436578 -1.276709969

[22,] 0.177978426 -0.838556486

[23,] 0.146126679 -0.219377600

[24,] 1.398570695 -0.488856360

[25,] 1.362890916 -0.003201643

[26,] 0.684653012 -1.159192993

[27,] 0.602302600 -1.867567821

[28,] 1.131835996 -1.846159642

[29,] 1.304987315 -1.366105647

[30,] 1.187135826 -0.909889268

[31,] 1.162751989 -0.447367603

[32,] 1.136435967 0.025556232

[33,] 1.030497032 0.522755766

[34,] 0.669893244 0.628326097

[35,] 0.098338049 0.589815743

[36,] 0.067176335 0.709905313

[37,] -0.026352297 0.752806385

[38,] -0.167577353 1.025993272

[39,] -0.226109272 1.479369551

[40,] -0.298036632 1.292863863

[41,] -0.362359354 1.209789567

[42,] -0.487241056 1.578961725

[43,] -0.293663624 1.301383023

[44,] -0.245847026 1.192380238

[45,] -0.067190165 1.002291842

[46,] -0.480191018 1.079572984

[47,] -0.256030506 1.110174709

[48,] -0.244766366 1.202818365

[49,] -0.181114559 1.380838209

[50,] -0.185120101 1.450497913

$pool_priors

[,1] [,2]

[1,] -3.951264 -1.117659

[2,] -3.386887 -1.452274

[3,] -3.499626 -1.382370

$mean_alpha_LtD

[1] 10.05128

$N_L

[1] 53

$yr

[1] 0 1 2 3 4 5 6 7 8 9 10 11 12 13 14 15 16 17 18 19 20 21 22 23 24 25 26 27 28 29 30 31 32 33 34 35 36 37 38 39 40 41 42 43 44 45 46 47 48 49

[51] 50 51 52

$lemmings

[,1] [,2] [,3]

[1,] 0.78900643 NA NA

[2,] 2.49844224 NA NA

[3,] 3.35316014 NA NA

[4,] -0.06571148 NA NA

[5,] 1.64372433 NA NA

[6,] 3.35316014 NA NA

[7,] 0.78900643 NA NA

[8,] 0.78900643 NA NA

[9,] 3.35316014 NA NA

[10,] -0.06571148 NA NA

[11,] 1.64372433 NA NA

[12,] 2.49844224 NA NA

[13,] -0.06571148 NA NA

[14,] 0.78900643 NA NA

[15,] 3.35316014 NA NA

[16,] 0.78900643 NA NA

[17,] -0.06571148 NA NA

[18,] 2.49844224 NA NA

[19,] 1.64372433 NA NA

[20,] -0.06571148 NA NA

[21,] 3.35316014 NA NA

[22,] -0.06571148 NA NA

[23,] 0.78900643 NA NA

[24,] 2.49844224 NA NA

[25,] -0.06571148 NA NA

[26,] -0.06571148 NA 0.3576744

[27,] 2.49844224 NA 1.5748465

[28,] 0.78900643 NA 0.4446858

[29,] 0.78900643 NA 0.9242589

[30,] 2.49844224 2.49844224 2.1713368

[31,] -0.06571148 -0.06571148 0.0000000

[32,] 1.64372433 2.49844224 NA

[33,] 1.64372433 -0.06571148 NA

[34,] -0.06571148 0.78900643 NA

[35,] 2.49844224 2.49844224 NA

[36,] -0.06571148 0.78900643 NA

[37,] 0.78900643 -0.06571148 NA

[38,] 0.78900643 1.64372433 1.0784096

[39,] -0.06571148 1.21636538 NA

[40,] -0.06571148 2.07108328 0.3987761

[41,] 2.49844224 3.35316014 2.3702437

[42,] -0.06571148 -0.06571148 0.0000000

[43,] 0.78900643 2.07108328 1.0152307

[44,] 0.78900643 NA 0.8796267

[45,] NA NA NA

[46,] NA 2.49844224 NA

[47,] NA 2.49844224 NA

[48,] NA -0.06571148 NA

[49,] NA NA NA

[50,] NA 3.35316014 NA

[51,] NA -0.06571148 NA

[52,] NA 1.21636538 NA

[53,] NA -0.06571148 NA

$indicator0

[1] 0 0 0 1 0 0 0 0 0 1 0 0 1 0 0 0 1 0 0 1 0 1 0 0 1 1 0 0 0 0 1 0 0 1 0 1 0 0 1 1 0 1 0 0 0 0 0 1 0 0 1 0 1

$indicator_peak_yrs

[1] 3 6 9 15 21

$indicator_not_peak_yrs

[1] 1 2 4 5 7 8 10 11 12 13 14 16 17 18 19 20 22 23 24 25 26 27 28 29 30 31 32 33 34 35 36 37 38 39 40 41 42 43 44 45 46 47 48 49 50 51 52 53

$n_peaks

[1] 5

$L_ini

[1] 2.498442

$Pre

, , 1

[,1] [,2] [,3] [,4] [,5] [,6] [,7] [,8] [,9] [,10] [,11] [,12]

[1,] 48.552436 64.589748 28.774732 14.319030 9.423264 29.760365 32.87278 51.88109 44.42653 21.29378 6.724276 15.538083

[2,] 31.463516 32.716411 21.788014 19.333132 17.488207 18.095632 18.55528 27.32848 33.93983 12.65778 7.811277 21.085952

[3,] 28.824731 8.161172 12.091737 12.265552 27.703065 28.340442 62.38937 36.55573 61.34694 18.27663 18.059364 22.479210

[4,] 20.928130 30.655697 28.752378 41.783506 22.530023 23.699318 48.85302 50.08167 48.49490 45.76401 45.459617 24.417513

[5,] 16.518121 20.669950 30.400574 24.861466 13.801944 42.954681 62.64542 60.04275 35.41301 16.47790 24.041260 34.626911

[6,] 19.606345 17.944042 14.428071 10.976530 9.839856 21.003518 66.94091 33.29532 26.53874 10.86464 30.764374 30.029805

[7,] 13.286374 13.099783 22.446856 13.312633 16.404127 19.784010 52.94610 19.41446 30.40584 24.72637 33.672393 30.576591

[8,] 67.444176 38.221748 17.433182 6.916205 17.211923 15.110160 40.25303 54.27454 60.91869 17.87848 41.829649 25.731430

[9,] 25.092600 47.849622 19.896280 33.845901 11.650395 18.232422 60.44643 45.05794 32.30872 29.93665 5.814019 64.952511

[10,] 18.519811 36.220548 28.198729 8.784783 30.940389 42.615536 18.94322 67.60562 36.31430 35.81971 16.526628 14.757793

[11,] 24.608509 38.706178 36.461655 18.618616 43.529660 27.303422 40.28722 32.53817 48.44125 26.61607 5.060543 26.908357

[12,] 35.569577 10.679509 13.485315 27.286418 10.447766 44.158482 33.58921 19.17711 35.39454 33.56673 28.883832 50.026896

[13,] 18.706250 27.102833 21.628549 32.964862 15.511764 48.801438 51.78507 59.58180 50.65206 16.25845 21.724955 21.832124

[14,] 10.470230 9.273945 27.493530 30.773250 22.123286 13.262979 20.18551 19.49588 33.29336 13.69564 33.706012 23.442646

[15,] 16.127516 14.550327 12.791210 5.767160 11.100016 15.012767 42.26566 34.28229 16.53841 25.11170 20.972501 11.050742

[16,] 12.286596 7.432531 8.873578 9.672933 7.974401 51.218990 36.02266 39.26971 34.16338 9.88947 18.173680 28.548994

[17,] 15.122885 28.410874 9.866846 13.172719 14.702698 36.759625 50.62614 66.95674 45.34881 34.49300 7.484476 23.078939

[18,] 95.448925 13.179989 9.983239 21.757758 8.257107 43.504951 15.21288 42.05958 24.73700 20.05345 27.123688 10.592360

[19,] 12.297876 14.897663 17.837687 23.644209 11.283297 40.542471 19.75538 68.84638 37.53502 18.11803 6.278997 18.138274

[20,] 22.511257 32.098682 17.350940 13.608395 12.728175 18.561712 47.58590 32.17912 28.43667 25.01427 31.193294 22.615264

[21,] 27.171159 37.586000 24.258704 11.688874 10.399876 16.676101 42.99713 26.33833 42.15095 13.84894 13.624482 23.000923

[22,] 15.923003 18.526441 21.359800 9.956384 4.999799 36.766785 23.93806 48.96586 45.14510 32.95217 24.116891 10.992151

[23,] 13.884186 12.341389 33.258483 25.633615 11.210322 35.813601 49.00006 51.25681 41.54132 22.93775 38.238875 14.945358

[24,] 32.922237 13.539151 29.383353 20.872545 20.928774 20.258215 22.81397 48.08940 21.78886 41.86729 28.864621 19.609111

[25,] 49.599916 26.963241 32.237771 9.571543 9.451893 22.353601 39.08837 33.12054 55.44928 35.60448 29.331395 61.625509

[26,] 35.840262 33.176565 31.154584 26.979604 25.998859 37.700525 55.99985 42.42714 48.81179 24.28981 23.957705 43.588473

[27,] 5.282068 15.544168 26.710109 31.922194 19.734313 18.519596 24.02277 59.28903 12.64925 21.93547 16.244670 30.505043

[28,] 34.764419 17.551721 13.300900 23.485526 16.301292 19.617352 22.62032 21.38854 31.23907 36.10845 13.885897 24.924023

[29,] 16.149472 16.382403 20.867530 6.363142 17.756551 15.291206 53.52657 38.98293 34.67578 11.49525 22.578941 29.260046

[30,] 40.754914 17.370517 28.823977 23.466159 13.619567 16.984671 27.33038 32.09911 22.33238 20.05889 37.637157 32.397499

[31,] 4.934073 2.581208 19.871775 9.347007 9.695632 7.137220 17.66319 27.27167 36.76525 36.55879 21.677798 12.804331

[32,] 31.944329 15.811901 31.113358 22.379063 10.943302 15.383310 13.65016 39.61763 30.59136 28.74311 30.738231 27.878870

[33,] 16.106244 23.316849 24.356870 8.069381 12.177936 26.867043 50.00096 28.54474 13.79105 17.72205 22.597927 17.007828

[34,] 31.665670 30.796743 21.389554 27.187036 15.037426 27.941699 21.95491 17.31036 28.05839 30.20348 17.844158 17.122134

[35,] 22.327101 8.200619 16.878996 13.064877 28.523548 32.751979 13.74013 31.03170 26.68305 12.72879 10.081922 27.602536

[36,] 8.746573 25.799761 9.572594 15.128793 15.970103 24.408890 40.53082 37.52739 37.38516 35.24942 24.382598 27.028884

[37,] 17.957813 21.072503 14.077073 27.867459 14.113284 32.622891 20.42749 24.74324 44.33281 21.56754 19.999307 8.725556

[38,] 18.186927 12.371567 10.160088 16.310893 20.729935 21.511288 36.41062 13.77774 41.42442 29.45303 27.033983 42.032768

[39,] 15.784287 22.130366 17.443016 22.203816 19.062725 28.900690 54.37055 48.66074 47.72293 31.73822 19.799903 14.755361

[40,] 42.581746 33.399842 42.068842 26.915913 26.966184 13.516072 13.45419 14.03780 28.52717 17.44524 18.098763 22.187269

[41,] 16.939804 16.097062 15.552371 12.042530 13.085209 21.830267 20.95238 32.45840 18.73465 33.02098 28.198213 32.640402

[42,] 31.879714 45.275836 21.991677 21.664095 33.976983 29.017536 36.74324 28.75275 40.01517 32.49424 37.435910 29.843521

[43,] 25.967408 33.433090 15.997197 6.131511 23.698551 25.175376 32.82748 26.84949 69.23473 44.28469 46.672164 41.142387

[44,] 29.342014 5.417659 27.258555 30.706056 15.058929 8.586473 31.98002 37.40933 38.45919 43.85490 29.208728 33.838406

[45,] 66.928295 45.850127 30.205470 23.345207 21.204204 29.488583 12.09441 21.47881 50.37190 35.86668 36.732675 54.639070

[46,] 79.850473 14.499937 21.818602 44.308874 18.522725 38.301075 39.76817 46.36596 56.03933 42.03091 22.502837 22.021424

[47,] 51.806019 8.622748 30.810860 39.965503 21.701301 42.125546 43.56216 47.89024 28.29338 19.97718 31.295584 46.533182

[48,] 37.166272 27.273024 40.917681 23.638294 14.318329 29.771084 39.26463 40.94818 29.76689 39.35546 31.584762 22.652468

[49,] 22.400072 32.154334 23.534762 15.952304 21.940964 29.263497 56.11490 51.37345 29.18143 20.66794 22.280456 30.972859

[50,] 36.237403 28.989841 7.406862 18.076283 28.706264 23.703420 15.92159 21.78507 23.49116 23.15975 24.650084 6.054599

[51,] 9.014621 11.916354 26.724736 30.421832 38.792977 21.755165 41.70094 65.53908 48.45476 14.57081 32.001481 23.137760

[52,] 13.471698 29.010313 35.054169 10.305564 23.930929 35.322084 64.44960 22.75186 44.25804 23.53473 43.347207 63.414911

[53,] 16.425915 27.499993 25.108773 34.748451 16.475736 29.941042 21.61007 58.28833 45.08493 41.93322 17.877382 9.525068

[54,] 49.773200 30.684152 16.958183 7.674832 6.678714 40.444550 12.81855 44.70122 27.35117 32.02640 11.789213 18.251671

, , 2

[,1] [,2] [,3] [,4] [,5] [,6] [,7] [,8] [,9] [,10] [,11] [,12]

[1,] 56.418796 72.195815 29.360605 13.572267 9.961808 31.008816 33.01978 57.93475 49.57473 25.42260 5.561456 15.124265

[2,] 35.339232 36.091234 22.684512 18.469017 19.882783 18.064662 17.29090 27.69216 37.72754 14.62490 6.602561 21.137585

[3,] 32.035522 8.765063 14.013036 16.268011 29.768897 31.200374 60.16949 42.68619 59.98177 22.77675 20.089616 22.952763

[4,] 25.292387 32.992242 29.098583 39.108319 22.465320 27.029886 57.81414 50.17305 53.30695 54.83877 38.665863 26.567909

[5,] 17.288835 23.652454 37.148197 23.301892 16.067334 35.184319 56.57288 66.45890 39.42813 19.63780 22.153300 28.446455

[6,] 21.491164 19.584611 18.890153 12.217676 10.175564 20.202660 67.39221 35.57001 30.59050 13.93295 28.608834 29.394678

[7,] 20.634552 17.487600 24.653089 12.490366 18.410228 19.836990 57.97578 18.15055 31.36582 27.80928 34.052359 36.096579

[8,] 74.833527 40.392300 17.783210 7.723304 17.769707 15.291881 40.85589 72.35696 73.19364 19.18461 40.385852 25.579895

[9,] 28.666113 46.519773 17.450478 30.195299 16.156134 20.341971 61.28397 44.42932 35.49570 37.37649 4.531591 60.666103

[10,] 19.658628 39.225760 27.763125 10.859086 34.579005 50.362474 20.96105 73.84084 44.50614 42.79304 16.584593 14.674918

[11,] 23.257769 36.243853 31.420583 17.700301 41.661805 28.816198 39.98057 29.88599 57.53688 29.89503 3.790152 28.618835

[12,] 40.328473 13.465381 15.652267 27.802333 11.855783 36.709403 33.86898 22.87767 39.35223 40.13693 24.195594 46.528918

[13,] 18.825244 28.860491 18.950543 34.292484 18.410236 49.654438 43.99146 59.98083 48.08206 19.04122 19.998011 16.406868

[14,] 12.133475 6.059829 31.030208 29.514734 29.655656 13.697042 21.37508 20.80481 37.33627 17.18222 26.571790 22.970565

[15,] 17.003522 16.721205 16.038310 5.705793 13.683908 17.959590 44.40711 36.58106 15.38620 28.69154 21.335172 12.252865

[16,] 17.135708 8.513960 9.382022 9.463540 8.221486 47.541568 36.10160 35.05760 29.51199 13.71884 17.869655 25.651668

[17,] 17.207898 30.720864 10.062149 14.577551 19.556128 39.679312 54.28772 67.66675 48.00010 44.39685 9.821395 31.319938

[18,] 98.140756 15.530457 12.804835 21.425746 8.280099 47.060807 17.83679 43.75102 28.56550 30.71223 26.707233 14.275727

[19,] 14.866577 17.291494 16.303802 24.764197 12.780233 60.937036 19.56725 83.99135 46.22823 24.33550 12.184349 25.073076

[20,] 29.229951 35.402075 14.107426 13.214447 13.494667 18.656106 53.70973 34.11980 31.07415 33.58012 34.205405 21.804189

[21,] 30.353156 43.130296 24.051614 9.371058 10.271706 15.992418 47.38350 34.88446 51.15497 17.82630 14.130185 26.858516

[22,] 21.576795 16.971107 21.067635 10.500522 7.488720 36.098313 30.28513 62.42373 51.67738 44.61399 24.164716 13.360335

[23,] 18.555536 13.716436 39.447078 25.021833 12.893775 35.079912 54.91544 48.38602 49.58746 30.12759 36.010296 14.310399

[24,] 34.164053 13.671033 28.029108 16.190051 26.290340 19.159057 23.22783 42.24685 22.40231 50.97006 25.636728 20.258361

[25,] 57.262874 28.374662 36.091603 8.672080 12.327921 26.902843 29.89017 33.45762 58.16591 40.96752 29.623723 59.064855

[26,] 37.502845 36.433517 32.189026 22.070016 32.885662 42.018524 55.88704 46.38360 54.18092 30.27890 25.888325 52.036988

[27,] 4.990315 16.445942 28.516666 27.651249 22.673615 16.506867 23.63933 62.82200 15.85391 25.97275 18.354823 41.171599

[28,] 37.005903 25.789983 11.674726 26.218428 19.754907 20.694624 23.17932 22.25718 44.28928 44.56366 12.929755 25.573464

[29,] 18.559970 16.347244 19.496460 4.604981 21.186251 17.237475 60.92142 42.97042 36.62634 16.34714 24.528572 34.199535

[30,] 51.992968 21.554102 35.569183 27.308536 17.504977 18.745505 28.63360 42.63800 26.89995 26.38568 44.192595 39.250493

[31,] 4.957824 2.464785 24.413204 10.347662 10.775834 7.131201 19.39764 30.48884 43.46511 48.15215 23.474593 12.914907

[32,] 39.420166 23.081614 37.485435 22.207151 13.684133 14.492964 13.39454 46.65840 34.90817 34.58539 34.372353 34.327988

[33,] 20.086679 32.511663 28.527920 9.459569 15.034958 31.604738 57.52131 32.18511 16.78070 26.48176 21.202541 20.430977

[34,] 40.556989 43.039244 24.092498 26.872968 17.960948 33.899862 25.70294 21.80816 31.79162 41.45482 20.727340 21.260473

[35,] 27.753725 8.508486 18.399271 15.564819 35.502515 40.866517 12.70586 48.93031 34.08964 16.45097 8.602455 34.958486

[36,] 8.416537 30.046671 12.692650 16.075595 20.129593 22.809795 47.28971 44.78179 41.89767 46.00278 24.780041 31.457416

[37,] 26.483169 26.674392 18.381131 30.783604 17.595514 35.640959 20.04970 30.47256 55.66818 31.21762 18.299508 6.913830

[38,] 16.943162 14.069390 11.587157 16.926195 22.716748 25.474971 42.55179 14.84417 55.43291 39.67437 32.825344 52.984379

[39,] 17.183999 23.637037 17.975874 21.446171 21.820067 30.022996 55.94068 52.57840 53.69916 39.55949 21.742296 14.781018

[40,] 45.965229 37.717699 45.294729 26.268834 30.314300 17.135438 16.01684 20.91416 34.44189 22.11953 17.996839 23.956568

[41,] 23.429677 23.611805 23.695762 12.235062 14.261263 35.253357 24.86874 38.83585 23.37874 39.67028 29.000759 32.886573

[42,] 30.846718 46.364874 23.016248 23.149311 35.017988 35.077468 44.52583 36.10077 47.70236 41.62777 36.980898 34.040494

[43,] 25.756041 40.477696 18.210301 4.915286 26.021957 31.261772 46.18903 31.31611 75.79541 46.86195 39.608011 48.296145

[44,] 39.954280 5.415571 25.168335 30.180866 17.762876 8.332209 33.84455 40.21784 41.45149 49.08400 31.433272 41.168869

[45,] 68.310750 45.801926 25.609611 24.590696 24.645859 29.422779 11.06428 25.76003 56.39242 44.34457 32.628696 62.824903

[46,] 79.368662 14.387296 26.710937 46.203725 20.692903 40.666303 49.84160 52.78494 63.81937 55.02950 23.702291 23.720160

[47,] 61.118230 9.520622 31.866503 39.924501 26.059002 47.773641 50.28045 48.66356 33.74946 25.93402 36.944457 52.436205

[48,] 44.640597 27.658467 42.439128 22.077301 18.045200 32.539611 44.63402 40.49475 28.47927 55.47466 33.474268 26.204364

[49,] 23.070796 29.055339 23.891612 15.329569 24.394635 32.453097 58.79440 55.64244 34.79364 25.13237 22.498130 22.944984

[50,] 37.874864 35.357716 9.076657 17.268858 30.882932 27.554712 14.15918 23.44222 25.41741 30.36584 22.895592 6.268475

[51,] 9.565905 11.119895 26.542562 27.480499 42.875104 26.878139 47.86030 66.11368 54.62527 16.51640 30.995774 22.195735

[52,] 11.823139 30.966175 38.138167 10.810816 28.313028 41.321087 69.45844 20.31267 49.93202 28.87970 41.999819 57.721057

[53,] 19.313947 38.779060 27.367049 38.717892 20.099961 22.466728 20.76713 54.12924 43.35182 55.18186 18.689201 11.469632

[54,] 53.120828 38.774193 20.905056 7.457433 13.883187 43.360469 14.43278 51.95660 32.54111 40.66755 15.570758 21.840708

, , 3

[,1] [,2] [,3] [,4] [,5] [,6] [,7] [,8] [,9] [,10] [,11] [,12]

[1,] 39.052433 53.793795 31.553787 14.393005 9.372560 24.652524 31.28362 42.46619 34.34176 18.139942 10.149836 18.044232

[2,] 28.237892 29.231407 22.891200 19.105753 13.867210 18.076000 22.98714 26.54694 27.45507 12.099288 11.021194 22.968656

[3,] 27.730315 8.248567 11.728105 11.219936 23.367002 23.388857 55.92564 31.10786 51.52737 14.888538 16.657660 22.834976

[4,] 19.921449 29.694485 31.046676 40.115365 19.006923 20.307347 38.45982 44.56168 39.47549 36.544927 46.175581 23.800748

[5,] 16.603158 20.101941 31.162247 25.014815 11.264899 41.183068 58.78333 49.37544 27.92508 14.214102 22.707140 38.307251

[6,] 19.204638 17.886720 14.166429 10.543429 8.544659 18.713294 58.82881 29.00093 21.48183 8.947637 30.241307 31.042396

[7,] 12.245353 11.557781 22.715618 12.952371 13.232812 18.284521 44.46959 19.08539 26.71494 20.196168 30.846499 27.372422

[8,] 60.114389 36.849126 18.370710 6.787062 15.000044 13.454422 36.98847 40.89816 45.61542 15.239335 41.323539 25.952804

[9,] 22.952912 46.843398 23.004135 32.644870 7.959723 14.200271 53.73713 39.49782 25.87632 22.848262 6.135777 64.534965

[10,] 17.054209 35.183835 30.348054 7.574811 24.077802 33.265176 15.87723 55.87967 27.58613 28.244061 15.295288 14.864633

[11,] 24.052599 38.208001 40.501141 16.640832 36.047204 22.307363 34.24924 30.52641 37.33252 21.113451 8.601190 26.654634

[12,] 31.596297 9.256739 13.556369 24.186775 8.403223 41.343398 28.43629 15.05535 28.67011 25.080585 28.715523 49.484996

[13,] 17.531429 25.043009 24.150896 28.864875 11.931825 39.675844 48.29456 50.44181 42.52856 12.784818 20.880429 24.411361

[14,] 10.391042 11.422987 25.880511 29.471154 16.336722 11.944724 17.87953 16.12655 26.31181 10.649409 35.374950 25.037110

[15,] 15.630270 13.429050 13.010863 5.575145 9.009143 12.540270 35.50029 27.73742 15.17311 20.088632 18.769603 10.370806

[16,] 9.957861 7.255641 9.019151 8.908150 6.805403 44.411409 31.31731 35.25445 30.49475 7.496387 16.835793 29.904609

[17,] 13.753040 26.256063 10.726598 11.713359 10.288178 30.858270 39.46406 57.53765 35.53377 25.676864 6.446013 20.067733

[18,] 89.861466 12.827684 9.187576 19.602866 7.318776 37.000639 13.65308 34.77769 19.49413 14.349796 25.386488 10.632306

[19,] 12.878437 14.796182 20.121267 21.283489 9.299598 27.249894 18.80918 51.33590 29.85645 13.518198 5.131796 15.747605

[20,] 20.287078 31.036379 20.935451 12.848842 10.931806 18.929245 40.26938 29.41891 23.14159 18.606986 26.985609 24.063978

[21,] 25.594657 36.557430 26.459902 12.319704 9.093885 15.377470 34.97651 21.31303 32.87050 10.731796 12.661752 22.765884

[22,] 14.126350 20.210058 23.298383 9.583903 4.207855 32.958298 18.41581 36.67735 36.67966 24.445810 24.495805 10.543492

[23,] 12.580975 12.824921 33.065292 24.862336 9.263315 31.777048 40.21154 44.96895 30.44751 16.250852 36.221563 15.477057

[24,] 32.451838 14.180593 33.358430 21.900995 15.663879 18.083471 20.88145 46.04542 18.66263 32.247324 28.485200 18.946987

[25,] 43.414940 27.553562 32.064342 9.444002 7.484680 18.241805 41.94834 29.55161 44.77667 28.947390 26.665003 60.808643

[26,] 34.192649 31.241122 32.394319 27.812541 18.587832 29.869037 48.21061 35.54330 37.79975 19.192526 21.707745 38.580234

[27,] 6.148388 15.606961 28.087756 31.864267 16.609582 17.133096 23.07250 49.41305 10.16637 17.597333 14.517719 25.335012

[28,] 32.781944 15.042369 15.658167 21.230227 12.873697 17.767094 20.27808 20.27868 21.37198 27.623844 14.158425 24.934129

[29,] 14.774683 17.009706 23.242645 7.071769 13.620323 12.588470 42.68794 30.85530 28.28978 8.267112 18.700590 26.074168

[30,] 32.614504 15.206327 26.722568 20.134168 10.358233 14.279296 22.94650 23.00752 17.14264 15.067244 31.674246 27.625149

[31,] 5.088913 3.229211 17.665609 8.120405 8.531393 6.501946 15.91581 21.38055 26.65715 27.005255 19.293466 12.987812

[32,] 27.793618 11.851884 29.410254 19.793226 8.431654 14.327085 12.48790 29.14181 22.53213 22.003907 24.948235 23.303900

[33,] 14.917480 20.027953 24.135659 6.923792 9.930252 19.498422 37.36245 22.53746 10.28162 11.393245 21.281185 14.239853

[34,] 26.721497 24.175199 21.461340 25.669119 11.670849 21.367022 17.53642 13.03948 22.37152 21.868043 14.900639 14.858007

[35,] 19.310246 7.929415 17.711512 10.464611 21.035458 23.010404 13.00556 22.08942 18.48205 9.793210 10.188476 23.322429

[36,] 8.566914 22.498420 8.751605 12.990822 11.960382 21.780268 31.77066 27.56029 27.78176 25.710876 23.485508 25.818361

[37,] 15.287941 19.589635 14.366169 23.140457 10.267285 26.830273 17.76854 18.96611 31.97638 14.728874 19.922925 9.603732

[38,] 18.827998 11.556651 10.044011 14.759391 17.090099 16.724334 28.82603 11.77304 28.09171 21.344057 21.480131 35.141970

[39,] 16.706675 21.923198 19.445111 20.813117 14.900635 24.833268 45.56631 40.25777 36.40559 23.455125 17.226177 15.451000

[40,] 36.704712 30.183296 39.712135 24.381795 20.945858 11.000264 11.79522 11.80757 22.62332 13.985496 17.722811 21.283612

[41,] 14.074995 13.018247 13.902826 10.667556 10.830435 15.136827 16.34975 25.89861 13.30084 26.078093 25.918408 32.094061

[42,] 31.883874 43.367185 23.253774 20.106191 28.214666 21.457843 28.00103 22.74895 33.29593 24.638434 34.522807 28.050754

[43,] 25.033420 29.575318 16.008000 6.286977 18.842396 19.422787 25.00034 21.54275 53.51596 36.517965 46.518940 35.686220

[44,] 24.352430 5.730932 30.927556 27.885828 11.485451 8.018194 27.99695 32.41269 33.46069 35.336058 24.948838 28.240492

[45,] 63.283905 45.006136 36.472699 20.377293 15.995960 24.899020 11.99376 18.30916 38.32544 26.648270 35.913255 48.285190

[46,] 80.747935 13.943524 19.702267 40.713488 15.144038 31.636807 32.91025 37.75417 43.97649 30.957909 19.649759 20.488586

[47,] 43.082432 8.040248 30.949670 35.229098 16.493874 34.388826 37.59714 44.38661 21.16863 15.097319 25.628946 42.974967

[48,] 30.460366 26.822425 41.640969 22.962602 10.660425 23.849289 31.64671 36.32418 26.06177 27.132501 27.564249 20.568959

[49,] 21.487925 34.504545 24.334150 14.795890 17.069230 25.810676 48.50451 42.57100 22.76895 15.967975 19.836927 36.623057

[50,] 34.403545 25.926630 7.402313 17.272254 22.700591 19.142968 17.03680 18.46199 20.77834 16.710349 23.642983 6.172906

[51,] 11.774744 14.229459 27.431701 27.374798 27.870569 18.988128 35.21099 52.90925 36.93721 13.061371 29.917168 23.956950

[52,] 14.527706 27.335380 35.722736 9.177458 18.067547 29.353319 56.20975 22.27602 35.45784 18.213113 39.544691 66.566066

[53,] 14.470638 22.300035 26.254112 28.148022 11.857294 29.715200 21.68034 54.00705 40.76759 29.390271 16.007337 8.846285

[54,] 47.220264 25.493574 15.850271 7.291552 4.204590 35.002433 12.63755 34.63710 20.93751 23.688581 10.269470 16.323783

$prec_pop

[1] 1 2 1

$chi_pop

[1] 1 1 1

$yr_pop

[1] 1 1 1

$n_pop

[1] 3

$n_clim_variable

[1] 2

$n_wL_para

[1] 2

$n_b

[1] 17

$Z_1

[,1] [,2] [,3] [,4] [,5] [,6] [,7] [,8] [,9] [,10] [,11] [,12] [,13] [,14] [,15] [,16] [,17]

[1,] 0 0 0 0 0 0 0 0 0 0 0 0 0 0 0 0 0

[2,] 0 0 0 0 0 0 0 0 0 0 0 0 0 0 0 0 0

[3,] 1 0 0 0 0 0 0 0 0 0 0 0 0 0 0 0 0

[4,] 0 0 0 0 0 0 0 0 0 0 0 0 0 0 0 0 0

[5,] 0 0 0 0 0 0 0 0 0 0 0 0 0 0 0 0 0

[6,] 0 1 0 0 0 0 0 0 0 0 0 0 0 0 0 0 0

[7,] 0 0 0 0 0 0 0 0 0 0 0 0 0 0 0 0 0

[8,] 0 0 0 0 0 0 0 0 0 0 0 0 0 0 0 0 0

[9,] 0 0 1 0 0 0 0 0 0 0 0 0 0 0 0 0 0

[10,] 0 0 0 0 0 0 0 0 0 0 0 0 0 0 0 0 0

[11,] 0 0 0 0 0 0 0 0 0 0 0 0 0 0 0 0 0

[12,] 0 0 0 1 0 0 0 0 0 0 0 0 0 0 0 0 0

[13,] 0 0 0 0 0 0 0 0 0 0 0 0 0 0 0 0 0

[14,] 0 0 0 0 0 0 0 0 0 0 0 0 0 0 0 0 0

[15,] 0 0 0 0 1 0 0 0 0 0 0 0 0 0 0 0 0

[16,] 0 0 0 0 0 0 0 0 0 0 0 0 0 0 0 0 0

[17,] 0 0 0 0 0 0 0 0 0 0 0 0 0 0 0 0 0

[18,] 0 0 0 0 0 1 0 0 0 0 0 0 0 0 0 0 0

[19,] 0 0 0 0 0 0 0 0 0 0 0 0 0 0 0 0 0

[20,] 0 0 0 0 0 0 0 0 0 0 0 0 0 0 0 0 0

[21,] 0 0 0 0 0 0 1 0 0 0 0 0 0 0 0 0 0

[22,] 0 0 0 0 0 0 0 0 0 0 0 0 0 0 0 0 0

[23,] 0 0 0 0 0 0 0 0 0 0 0 0 0 0 0 0 0

[24,] 0 0 0 0 0 0 0 1 0 0 0 0 0 0 0 0 0

[25,] 0 0 0 0 0 0 0 0 0 0 0 0 0 0 0 0 0

[26,] 0 0 0 0 0 0 0 0 0 0 0 0 0 0 0 0 0

[27,] 0 0 0 0 0 0 0 0 1 0 0 0 0 0 0 0 0

[28,] 0 0 0 0 0 0 0 0 0 0 0 0 0 0 0 0 0

[29,] 0 0 0 0 0 0 0 0 0 0 0 0 0 0 0 0 0

[30,] 0 0 0 0 0 0 0 0 0 1 0 0 0 0 0 0 0

[31,] 0 0 0 0 0 0 0 0 0 0 0 0 0 0 0 0 0

[32,] 0 0 0 0 0 0 0 0 0 0 0 0 0 0 0 0 0

[33,] 0 0 0 0 0 0 0 0 0 0 1 0 0 0 0 0 0

[34,] 0 0 0 0 0 0 0 0 0 0 0 0 0 0 0 0 0

[35,] 0 0 0 0 0 0 0 0 0 0 0 0 0 0 0 0 0

[36,] 0 0 0 0 0 0 0 0 0 0 0 1 0 0 0 0 0

[37,] 0 0 0 0 0 0 0 0 0 0 0 0 0 0 0 0 0

[38,] 0 0 0 0 0 0 0 0 0 0 0 0 0 0 0 0 0

[39,] 0 0 0 0 0 0 0 0 0 0 0 0 1 0 0 0 0

[40,] 0 0 0 0 0 0 0 0 0 0 0 0 0 0 0 0 0

[41,] 0 0 0 0 0 0 0 0 0 0 0 0 0 0 0 0 0

[42,] 0 0 0 0 0 0 0 0 0 0 0 0 0 1 0 0 0

[43,] 0 0 0 0 0 0 0 0 0 0 0 0 0 0 0 0 0

[44,] 0 0 0 0 0 0 0 0 0 0 0 0 0 0 0 0 0

[45,] 0 0 0 0 0 0 0 0 0 0 0 0 0 0 1 0 0

[46,] 0 0 0 0 0 0 0 0 0 0 0 0 0 0 0 0 0

[47,] 0 0 0 0 0 0 0 0 0 0 0 0 0 0 0 0 0

[48,] 0 0 0 0 0 0 0 0 0 0 0 0 0 0 0 1 0

[49,] 0 0 0 0 0 0 0 0 0 0 0 0 0 0 0 0 0

[50,] 0 0 0 0 0 0 0 0 0 0 0 0 0 0 0 0 0

[51,] 0 0 0 0 0 0 0 0 0 0 0 0 0 0 0 0 1

[52,] 0 0 0 0 0 0 0 0 0 0 0 0 0 0 0 0 0

[53,] 0 0 0 0 0 0 0 0 0 0 0 0 0 0 0 0 0

$mean_alpha_L

[1] 5.249631 -7.008300 -7.941879

$mean_chi_L

[1] -0.04134554

$mean_betaC

[,1] [,2] [,3]

[1,] 1.003370 -0.04427962 0.03693964

[2,] -2.398814 2.34410967 2.69779010

$mean_beta_yr

[1] -0.005852561
