# Supplementary material for: Large-scale changes in marine and terrestrial environments drive the population dynamics of long-tailed ducks breeding in Siberia
Source: Sci Rep. 2022 Jul 19;12:12355. doi: 10.1038/s41598-022-16166-7 (PMC9296647; doi:10.1038/s41598-022-16166-7)
Supplement: Supplementary file 1 — Supplementary Information. [file 41598_2022_16166_MOESM1_ESM.zip › data_mus.docx]

$N

[1] 32

$len

[1] 35

$n_knots

[1] 5

$mus2

[,1] [,2]

[1,] 10.596635 NA

[2,] 10.201739 NA

[3,] 9.642123 NA

[4,] 10.229909 NA

[5,] 10.597634 NA

[6,] 10.223395 NA

[7,] 11.028417 NA

[8,] 11.408564 NA

[9,] 11.670271 NA

[10,] 11.100738 NA

[11,] 10.740735 NA

[12,] 9.354441 NA

[13,] 11.077207 10.233870

[14,] 11.096076 10.551873

[15,] 10.799576 10.151324

[16,] 10.373491 10.285172

[17,] 9.717158 9.686140

[18,] 9.441452 9.397484

[19,] 8.672486 9.425613

[20,] 8.853665 8.934323

[21,] 8.963928 9.098291

[22,] 9.379070 9.422868

[23,] NA 9.313168

[24,] NA 9.428110

[25,] NA 8.705662

[26,] NA 8.716044

[27,] NA 9.104980

[28,] NA 9.802506

[29,] NA 10.203592

[30,] NA 10.341742

[31,] NA NA

[32,] 11.270854 NA

$fer

[1] 5.788341 5.764639 5.742250 5.742250 5.915091 5.922996 5.961611 5.954007 5.932402 5.905523 5.877901 5.849495 5.832398 5.815008 5.797309 5.731900

[17] 5.718655 5.677833 5.620674 5.605859 5.590821 5.575554 5.528301 5.620674 5.620674 5.605859 5.590821 5.590821 5.560050 5.560050 5.590821 5.605859

[33] 5.677833 5.649662 5.635273

$s_var

[1] 0.5161525

$T_mx_Wi

[,1] [,2] [,3]

[1,] 4.9560476 4.45282796 3.0950931

[2,] 4.8598426 -1.27155261 1.0454278

[3,] 6.8175157 3.10995807 -1.7441622

[4,] 5.9830062 -1.29634529 3.1945101

[5,] 5.5180782 6.92084327 5.6602695

[6,] 7.1719237 6.60346385 7.4500085

[7,] 6.1950824 6.68257886 9.5399816

[8,] 5.2055151 4.93465723 1.6906115

[9,] 5.6363070 4.98944782 6.8670665

[10,] 4.8768007 5.89509818 3.5240220

[11,] 5.7138092 5.98986914 2.5154087

[12,] 7.0589566 4.38216383 7.6606033

[13,] 0.6256867 -0.02991736 0.8177981

[14,] 1.9186065 1.26794094 7.2771878

[15,] 5.5402494 6.21199371 8.2952110

[16,] 4.8356552 6.27788149 4.5605582

[17,] 5.9872024 5.69462295 7.3041164

[18,] 6.4163506 4.08333570 5.5704886

[19,] 4.1157616 5.65817423 8.5916325

[20,] 2.7080813 3.82329270 3.0931049

[21,] 6.2640495 3.70492974 6.1755009

[22,] 5.5314038 6.31551592 3.3802789

[23,] 5.1494651 2.11297854 3.5875009

[24,] 8.5263696 8.36760990 6.5628573

[25,] 5.4026902 7.19541930 7.5158905

[26,] 4.5001094 2.69919269 4.3527490

[27,] 3.4891015 -0.18539185 2.1678978

[28,] -0.1598473 4.15076719 4.6342263

[29,] 7.3872411 5.56389520 2.6802084

[30,] 5.0172422 3.28017170 2.5902738

[31,] 7.6331792 5.41621919 8.5078265

[32,] 6.1400306 5.39929950 5.1248674

[33,] 10.4986918 4.52838348 6.2117161

[34,] 7.0123607 4.27623095 6.0628148

$T_mn_Wi

[,1] [,2] [,3]

[1,] 0.09198035 0.30368501 -1.1544461

[2,] 1.08812856 -6.92851102 -5.1358772

[3,] 2.61423077 -1.54356856 -7.8327874

[4,] 1.30682689 -7.07116434 -1.9741344

[5,] 1.48213946 2.83903121 1.2808222

[6,] 3.18452054 2.29864111 2.1442671

[7,] 0.98694297 2.86154309 3.7038235

[8,] 1.11550191 0.61213800 -4.0637934

[9,] 0.67698660 0.34876451 1.8195416

[10,] 1.10908839 0.56727171 -0.7495644

[11,] 1.75911677 2.10786106 -2.7613555

[12,] 2.35694845 -0.47339833 2.7162283

[13,] -4.14694819 -4.01061873 -4.4023885

[14,] -2.84074089 -4.10196396 2.3376646

[15,] 1.51177993 1.53855988 3.2577125

[16,] 0.34837588 1.65395489 -0.5552414

[17,] 1.36458017 1.33166500 2.2946240

[18,] 2.37014688 -0.01428745 -0.2270055

[19,] -0.92121431 1.39799689 3.0434790

[20,] -1.09257029 -1.24681968 -3.4000928

[21,] 1.09470950 -0.64998216 0.7825012

[22,] 1.01467202 2.01795993 -1.3637364

[23,] 0.95635287 -2.72670564 -0.8126235

[24,] 4.70735766 3.64363135 2.1538619

[25,] 1.66871392 3.10019222 1.9963880

[26,] 0.43050374 -1.81150284 -0.3645397

[27,] -0.71346489 -4.54330621 -2.5349978

[28,] -5.34205974 0.03777694 -0.3759635

[29,] 3.06193994 1.21043510 -3.2364989

[30,] 0.53748417 -0.83555265 -1.3609760

[31,] 3.10210062 1.06549629 2.4830266

[32,] 1.86906877 1.24596564 -0.2491181

[33,] 6.11838843 -0.03500762 1.1005942

[34,] 2.18736915 -0.70614679 0.8843172

$obs_j

[1] 1 2

$Z

[,1] [,2] [,3] [,4] [,5]

[1,] 0.0042990402 0.0081603191 2.823810e-02 0.029853222 0.0203684056

[2,] 0.0088645579 0.0157154631 3.139768e-02 0.017898681 0.0118730259

[3,] 0.0149095010 0.0265897212 3.001384e-02 0.010108069 0.0062376024

[4,] 0.0149095010 0.0265897212 3.001384e-02 0.010108069 0.0062376024

[5,] 0.0008596549 -0.0016309675 1.187303e-02 0.047991994 0.1678886792

[6,] 0.0027109863 -0.0042359073 1.114518e-02 0.043785307 0.1851710180

[7,] 0.0186793961 -0.0271088764 4.506754e-03 0.012307870 0.2869218462

[8,] 0.0145023658 -0.0211673627 6.250011e-03 0.020042183 0.2644953177

[9,] 0.0054775620 -0.0082008395 1.001589e-02 0.037841675 0.2072463311

[10,] -0.0008605711 0.0006989981 1.251149e-02 0.052162754 0.1484649473

[11,] -0.0031289032 0.0035642783 1.362297e-02 0.058676416 0.1010627913

[12,] -0.0023899502 0.0033567324 1.545928e-02 0.057042016 0.0645834765

[13,] -0.0010473486 0.0032306843 1.789868e-02 0.052110030 0.0479919940

[14,] 0.0006926047 0.0040446498 2.177414e-02 0.044353155 0.0347833720

[15,] 0.0029314133 0.0063211035 2.620830e-02 0.034835091 0.0245386764

[16,] 0.0186082628 0.0323495588 2.821650e-02 0.007663403 0.0042462619

[17,] 0.0244777300 0.0398752326 2.526634e-02 0.005432838 0.0021791256

[18,] 0.0535757417 0.0589996891 1.571546e-02 0.003230684 -0.0016309675

[19,] 0.1334728476 0.0593358627 1.023541e-02 0.001900672 -0.0012245009

[20,] 0.1631454125 0.0535757417 8.864558e-03 -0.001047349 0.0008596549

[21,] 0.1974777054 0.0451742426 6.790917e-03 -0.006124390 0.0041975593

[22,] 0.2369496293 0.0338058350 3.856925e-03 -0.013711128 0.0090989939

[23,] 0.3916693732 -0.0221321129 -1.129712e-02 -0.055250476 0.0368065328

[24,] 0.1334728476 0.0593358627 1.023541e-02 0.001900672 -0.0012245009

[25,] 0.1334728476 0.0593358627 1.023541e-02 0.001900672 -0.0012245009

[26,] 0.1631454125 0.0535757417 8.864558e-03 -0.001047349 0.0008596549

[27,] 0.1974777054 0.0451742426 6.790917e-03 -0.006124390 0.0041975593

[28,] 0.1974777054 0.0451742426 6.790917e-03 -0.006124390 0.0041975593

[29,] 0.2820868599 0.0190835025 -5.164554e-05 -0.024164599 0.0159107637

[30,] 0.2820868599 0.0190835025 -5.164554e-05 -0.024164599 0.0159107637

[31,] 0.1974777054 0.0451742426 6.790917e-03 -0.006124390 0.0041975593

[32,] 0.1631454125 0.0535757417 8.864558e-03 -0.001047349 0.0008596549

[33,] 0.0535757417 0.0589996891 1.571546e-02 0.003230684 -0.0016309675

[34,] 0.0864838270 0.0635892648 1.228312e-02 0.003614845 -0.0025984573

[35,] 0.1080451176 0.0626294807 1.125186e-02 0.003269850 -0.0023037411
