# Supplementary material for: Large-scale changes in marine and terrestrial environments drive the population dynamics of long-tailed ducks breeding in Siberia
Source: Sci Rep. 2022 Jul 19;12:12355. doi: 10.1038/s41598-022-16166-7 (PMC9296647; doi:10.1038/s41598-022-16166-7)
Supplement: Supplementary file 1 — Supplementary Information. [file 41598_2022_16166_MOESM1_ESM.zip › inits_din.docx]

[[1]]

[[1]]$st_din

[,1] [,2]

[1,] 0.22864301 0.07885160

[2,] 1.58623743 -0.87213759

[3,] -0.82065470 0.58717094

[4,] -1.27605287 0.54367678

[5,] 2.32576223 -0.75232974

[6,] -0.89442706 -0.50781395

[7,] -1.60486684 -2.35374692

[8,] 0.41102560 -0.63396321

[9,] -1.20236626 0.58261307

[10,] -0.70966935 0.19676832

[11,] -0.56300140 -1.49713682

[12,] -0.48625202 -1.09302229

[13,] -1.02625364 0.10991808

[14,] -0.07791581 0.30879930

[15,] 0.97546464 -0.72403546

[16,] 0.94625285 1.42021539

[17,] -0.08139085 -0.44212750

[18,] 0.14924838 -1.19559499

[19,] 0.69965005 -0.43815976

[20,] -1.11165682 0.99536026

[21,] 0.55784161 -0.34054625

[22,] 0.63244362 0.79910029

[23,] -1.00618268 -1.39234485

[24,] 0.63641345 1.23175523

[25,] -0.64455730 -0.62086181

[26,] -2.12121790 -0.35609775

[27,] 0.59435778 -0.45092534

[28,] -0.39974735 -1.20734364

[29,] -0.08378377 -0.24706777

[30,] 0.41101711 0.12166512

[31,] 0.11608960 -0.48708158

[32,] -1.32062016 0.25929609

[33,] 0.21600738 -0.52970638

[34,] 0.89552885 0.35820407

[35,] 0.53068515 -0.06402176

[36,] -0.44258640 -0.82821995

[37,] 1.32135212 0.13849989

[38,] 1.15938940 -1.58592708

[39,] -0.60686025 0.91049955

[40,] -0.48326377 1.35818281

[41,] 0.61907774 -0.41031811

[42,] -1.17700867 -0.39164589

[43,] 1.19343030 -1.57796600

[44,] -0.79721097 -1.00613682

[45,] 1.40517382 0.50165769

[46,] 0.14191622 0.93522776

[47,] 0.40978695 0.11250267

[[2]]

[[2]]$st_din

[,1] [,2]

[1,] -1.3155994 -0.38330780

[2,] -0.5657169 -1.06502911

[3,] -0.1197658 -1.31382880

[4,] 0.7335246 -0.32511438

[5,] 0.4903617 1.71596121

[6,] -2.1532261 -1.06640713

[7,] -0.4975491 1.57466654

[8,] 0.8301961 0.29482576

[9,] -1.1169443 -0.27630126

[10,] 1.4704447 0.95121709

[11,] -0.8604230 1.52926330

[12,] -0.4194121 0.31611741

[13,] -1.4334384 -0.90171293

[14,] -0.5385498 -0.79593337

[15,] -0.4309086 -0.35199782

[16,] 0.4517788 -0.55852600

[17,] -1.9637526 -3.88961315

[18,] -1.3305267 1.09204608

[19,] 0.6099032 -0.84887432

[20,] 0.3207042 0.15113623

[21,] -1.7339108 0.30224934

[22,] -1.2538224 -0.59877063

[23,] -0.4328578 0.88540805

[24,] -1.5152278 -1.99685821

[25,] 0.8577970 1.69590531

[26,] 2.5958476 0.36620859

[27,] 0.1781876 0.19125450

[28,] -0.1227319 -1.70945514

[29,] -0.3541402 0.71991931

[30,] 0.8027179 0.02916216

[31,] -0.2767439 -1.33631782

[32,] -0.7924787 -1.41774166

[33,] 1.0921427 0.75079255

[34,] 1.2566279 0.64627192

[35,] 0.6435945 0.08030856

[36,] -0.1145687 -0.64658342

[37,] 1.4971337 -0.68171646

[38,] -1.0441182 -0.75467135

[39,] 0.8647336 0.07055975

[40,] -0.9757116 1.22378714

[41,] 1.2016931 -0.49166199

[42,] -1.0156296 -0.47941080

[43,] 1.1920107 0.07840574

[44,] -1.1485380 -0.50074959

[45,] -2.7109138 0.14424762

[46,] 0.9674611 0.28429025

[47,] 0.6804183 -0.26858847
