# Supplementary material for: Large-scale changes in marine and terrestrial environments drive the population dynamics of long-tailed ducks breeding in Siberia
Source: Sci Rep. 2022 Jul 19;12:12355. doi: 10.1038/s41598-022-16166-7 (PMC9296647; doi:10.1038/s41598-022-16166-7)
Supplement: Supplementary file 1 — Supplementary Information. [file 41598_2022_16166_MOESM1_ESM.zip › inits_fs.docx]

[[1]]

[[1]]$alpha_f

[1] 0.6087849

[[1]]$eps_rnd_f

[1] -0.7621729 -0.9274246 0.3027671 0.4841992 -0.1057837 -0.4854407 -1.0602817 0.5777284 2.5568072 0.5965891 0.4003102 -0.2203178 -0.2843749

[14] 0.1099261 1.1751363 0.4512994

[[1]]$sigma_f_rnd

[1] 0.07214156

[[1]]$sigma_f

[1] 9.247168

[[1]]$logit_f_obs

[1] NA NA NA NA NA NA NA NA NA NA NA NA NA NA NA NA

[[1]]$f_beta

[1] 0.2445579 0.6004009 0.5358762

[[2]]

[[2]]$alpha_f

[1] 1.467785

[[2]]$eps_rnd_f

[1] 2.04785822 -0.06856724 1.25105166 -0.87799312 -0.27739429 0.78589400 -0.91687498 -0.94204876 0.13422782 -0.30101900 -0.14056945 -1.39348841

[13] 0.67401484 -0.57805401 0.40281043 0.39972563

[[2]]$sigma_f_rnd

[1] 0.6530093

[[2]]$sigma_f

[1] 2.878284

[[2]]$logit_f_obs

[1] NA NA NA NA NA NA NA NA NA NA NA NA NA NA NA NA

[[2]]$f_beta

[1] 0.1646011 0.5510261 0.6965805
