# Supplementary material for: Large-scale changes in marine and terrestrial environments drive the population dynamics of long-tailed ducks breeding in Siberia
Source: Sci Rep. 2022 Jul 19;12:12355. doi: 10.1038/s41598-022-16166-7 (PMC9296647; doi:10.1038/s41598-022-16166-7)
Supplement: Supplementary file 1 — Supplementary Information. [file 41598_2022_16166_MOESM1_ESM.zip › inits_ltd.docx]

[[1]]

[[1]]$sigma_juv

[1] 0.4518168 0.6865173

[[1]]$alpha_juv

[1] 0.7768678

[[1]]$beta_nao3

[1] 0.3112362

[[1]]$beta_L

[1] 0.9448335

[[1]]$bCj

[1] -0.2534494 -0.4421448

[[1]]$wj

[1] 0.4991671 0.4522193

[[1]]$logitp_obs

[1] 0.28596824 -1.36474762 0.56531749 -0.85838363 -0.03561586 0.27732685 -0.68552754 -0.29333247 0.68472288 -0.60840587 -0.63943501 -2.84587159

[13] -0.26929981 -1.11366117 -0.33751154 NA NA NA NA NA NA NA NA NA

[25] NA NA NA NA NA NA NA NA NA NA NA NA

[37] NA NA NA NA NA NA NA NA NA NA NA NA

[49] NA NA NA

[[1]]$beta_yr

[1] 1.339955

[[1]]$sigma_oLTD

[1] 0.5855896

[[1]]$alpha_LtD

[1] -0.5103251

[[1]]$sigma_pLTD

[1] 0.5329684

[[1]]$beta_juv

[1] 1

[[1]]$dd_LtD

[1] 0.1529577

[[1]]$beta_o_east

[1] -0.2152308

[[1]]$state_ltd

[1] 143377 143699 143077 144075 143215 143488 142780 144025 143103 144368 143277 143267 143537 143426 142973 143064 143564 144408 143712 144053 143753

[22] 143663 143603 143414 143919 143420 143272 144038 143452 143478 143752 143587 143514 144470 144853 143368 143689 143752 143436 143893 143870 143528

[43] 144464 143330 144379 143531 142970

[[1]]$n

[1] NA NA NA 11.44550 NA NA NA 12.65885 NA NA NA NA NA NA NA NA

[17] NA NA NA NA NA NA NA NA NA NA NA NA NA NA NA NA

[33] NA NA NA NA NA NA NA NA NA NA NA NA NA NA NA

[[1]]$beta_din

[1] -0.5666642

[[1]]$wD

[1] 0.5739872

[[1]]$pool

[,1] [,2]

[1,] -4.098293 -1.477728

[2,] -4.098293 -1.477728

[3,] -4.098293 -1.477728

[4,] NA NA

[5,] NA NA

[6,] NA NA

[7,] NA NA

[8,] NA NA

[9,] NA NA

[10,] NA NA

[11,] NA NA

[12,] NA NA

[13,] NA NA

[14,] NA NA

[15,] NA NA

[16,] NA NA

[17,] NA NA

[18,] NA NA

[19,] NA NA

[20,] NA NA

[21,] NA NA

[22,] NA NA

[23,] NA NA

[24,] NA NA

[25,] NA NA

[26,] NA NA

[27,] NA NA

[28,] NA NA

[29,] NA NA

[30,] NA NA

[31,] NA NA

[32,] NA NA

[33,] NA NA

[34,] NA NA

[35,] NA NA

[36,] NA NA

[37,] NA NA

[38,] NA NA

[39,] NA NA

[40,] NA NA

[41,] NA NA

[42,] NA NA

[43,] NA NA

[44,] NA NA

[45,] NA NA

[46,] NA NA

[47,] NA NA

[48,] NA NA

[49,] NA NA

[50,] NA NA

[[1]]$alpha_L_sc

[1] 0.2944781 0.5893911 0.3538002

[[1]]$chi_L

[1] 0.004182754

[[1]]$wL

[1] 0.5638745 0.5791477

[[1]]$sigma_L_o

[1] 6.944628e-05 5.388812e-05 3.064399e-05

[[1]]$sigma_L_rnd

[1] 5.395775e-05 1.400602e-05 5.734714e-05

[[1]]$L_st

[,1] [,2] [,3]

[1,] 9 10 8

[2,] 8 9 7

[3,] 9 9 9

[4,] 10 10 7

[5,] 8 9 9

[6,] 9 7 10

[7,] 11 10 8

[8,] 8 9 10

[9,] 10 9 10

[10,] 10 8 9

[11,] 8 10 9

[12,] 9 8 10

[13,] 10 9 10

[14,] 10 8 9

[15,] 9 8 7

[16,] 9 10 9

[17,] 10 9 9

[18,] 9 10 8

[19,] 8 8 8

[20,] 9 9 7

[21,] 8 9 10

[22,] 8 7 10

[23,] 9 9 8

[24,] 10 9 10

[25,] 8 9 9

[26,] 7 8 9

[27,] 9 9 9

[28,] 9 9 8

[29,] 9 8 8

[30,] 7 9 8

[31,] 10 9 8

[32,] 9 10 10

[33,] 9 8 9

[34,] 9 9 10

[35,] 11 8 9

[36,] 9 10 8

[37,] 11 9 9

[38,] 9 10 9

[39,] 8 10 8

[40,] 10 9 9

[41,] 9 10 9

[42,] 8 7 10

[43,] 10 9 10

[44,] 8 10 10

[45,] 10 11 8

[46,] 10 11 9

[47,] 9 9 9

[48,] 10 9 9

[49,] 7 9 10

[50,] 9 10 9

[51,] 11 8 8

[52,] 9 10 10

[53,] 8 8 9

[[1]]$rnd_L

[,1] [,2] [,3]

[1,] 0.69418193 0.067766994 0.07274529

[2,] -0.43294951 -0.305820331 2.05863128

[3,] -0.76824472 -0.642549614 1.00633925

[4,] 1.32726346 -0.180666600 -1.09927825

[5,] -0.05537455 -1.360969473 -1.18371455

[6,] -0.18452797 0.885048098 0.58937864

[7,] -0.87042081 0.017471932 1.77135495

[8,] -1.61013680 -1.732125328 -0.67286688

[9,] -1.11745715 -0.384544728 0.41969084

[10,] 0.70471815 -1.158690015 -0.95926624

[11,] 0.23954072 -0.160855654 0.78274847

[12,] 1.22027941 -0.463428526 1.63786199

[13,] 0.38086512 -0.335856298 0.56337430

[14,] 1.77420529 0.757303507 1.74130051

[15,] 0.61047621 -1.081211764 -0.29225225

[16,] 0.98623466 0.215821979 0.87425360

[17,] -0.61241499 -0.561089333 -1.97252638

[18,] -1.24669001 -0.994700693 -0.04473913

[19,] -0.46410006 -1.657570693 -0.18666603

[20,] -0.65208281 -0.338819595 -0.42555316

[21,] -1.70736671 0.351073715 1.03356641

[22,] -0.26740600 0.340349977 -0.80281697

[23,] 0.94601342 -1.478348895 1.04028604

[24,] 0.38304315 0.550031292 0.06498363

[25,] 1.52309573 -0.764083270 0.84180865

[26,] 1.91281988 -0.175926184 -0.19036290

[27,] 0.25135275 -0.343867238 1.36555234

[28,] 0.68172835 0.930483977 2.00381672

[29,] -0.66273467 0.244019145 -0.30570937

[30,] -0.86200153 -0.634878889 -0.92279309

[31,] 0.81494806 -0.350236661 1.49890196

[32,] 0.42996408 -1.219118710 -0.14964631

[33,] 0.78072267 -1.523264850 -0.85998724

[34,] -0.74995958 0.671021396 1.28925998

[35,] 0.22092278 -0.832121546 1.96500817

[36,] 2.00818590 1.007061560 0.65652728

[37,] -0.52035802 -0.425484859 0.01252331

[38,] -1.19939657 1.173289232 -0.41067633

[39,] 1.25807146 -1.002183678 -0.62902889

[40,] -0.57670088 -0.719340535 -0.73363131

[41,] -0.48370530 -1.230905602 -0.05546046

[42,] -3.05931015 1.979531996 1.88115772

[43,] -0.78314654 -0.298730387 1.68367268

[44,] 0.48404114 -0.865448718 -1.30903474

[45,] 0.17679090 -1.360252073 -1.17995087

[46,] 0.79956784 -0.002727912 0.26241860

[47,] -1.51811358 -0.145750233 -0.08785107

[48,] 1.24159778 -1.513162057 -0.25875099

[49,] -0.37807712 -1.389834131 -0.96703943

[50,] -0.01684637 -0.076319552 -1.07322077

[51,] 1.89343533 -1.209441411 -1.79724738

[52,] -1.46374491 -1.089432813 1.67159126

[53,] -0.80087391 0.467491142 0.75136068

[[1]]$lemmings

[,1] [,2] [,3]

[1,] NA 1.83806156 0.08006383

[2,] NA 0.93104507 0.10177887

[3,] NA 2.30264308 0.68060690

[4,] NA 1.39249786 1.63398987

[5,] NA 0.72424897 0.60341039

[6,] NA 1.08751358 2.76193103

[7,] NA 1.10134435 1.55943460

[8,] NA 1.37839520 0.28862235

[9,] NA 3.03506674 0.52978236

[10,] NA 6.52610070 0.54125586

[11,] NA 4.09818621 1.21432810

[12,] NA 1.27085724 0.24884648

[13,] NA 1.28609568 6.82324597

[14,] NA 1.92538105 0.50355871

[15,] NA 1.00139435 0.98090632

[16,] NA 2.74031075 0.35315229

[17,] NA 1.38808060 0.90323711

[18,] NA 0.79521444 0.70230881

[19,] NA 0.77527768 1.12363553

[20,] NA 1.04161606 0.49849806

[21,] NA 1.74351746 0.42497071

[22,] NA 1.02372335 0.32241368

[23,] NA 0.44498238 0.37713931

[24,] NA 0.09973082 1.06183407

[25,] NA 1.34006886 0.99656185

[26,] NA 0.83221095 NA

[27,] NA 5.33993163 NA

[28,] NA 2.33251228 NA

[29,] NA 5.08137918 NA

[30,] NA NA NA

[31,] NA NA NA

[32,] NA NA 0.27736058

[33,] NA NA 2.26865174

[34,] NA NA 1.37409269

[35,] NA NA 0.75513802

[36,] NA NA 2.79855731

[37,] NA NA 0.44986234

[38,] NA NA NA

[39,] NA NA 1.11401105

[40,] NA NA NA

[41,] NA NA NA

[42,] NA NA NA

[43,] NA NA NA

[44,] NA 3.54005277 NA

[45,] 0.9841415 0.38028983 0.57367648

[46,] 3.6712661 NA 1.89709883

[47,] 1.4428200 NA 0.12459621

[48,] 0.4857245 NA 2.24432361

[49,] 1.6296049 0.09140167 0.36326682

[50,] 0.8329119 NA 1.41177701

[51,] 2.0030606 NA 0.35221132

[52,] 1.9851532 NA 2.22732534

[53,] 8.5204161 NA 1.05156043

[[1]]$tau0

[,1]

[1,] 0.6523802

[2,] 0.6523802

[3,] 0.6523802

[4,] 0.6523802

[5,] 0.6523802

[6,] 0.6523802

[7,] 0.6523802

[8,] 0.6523802

[9,] 0.6523802

[10,] 0.6523802

[11,] 0.6523802

[12,] 0.6523802

[13,] 0.6523802

[14,] 0.6523802

[15,] 0.6523802

[16,] 0.6523802

[17,] 0.6523802

[18,] 0.6523802

[19,] 0.6523802

[20,] 0.6523802

[21,] 0.6523802

[22,] 0.6523802

[23,] 0.6523802

[24,] 0.6523802

[25,] 0.6523802

[26,] 0.6523802

[27,] 0.6523802

[28,] 0.6523802

[29,] 0.6523802

[30,] 0.6523802

[31,] 0.6523802

[32,] 0.6523802

[33,] 0.6523802

[34,] 0.6523802

[35,] 0.6523802

[36,] 0.6523802

[37,] 0.6523802

[38,] 0.6523802

[39,] 0.6523802

[40,] 0.6523802

[41,] 0.6523802

[42,] 0.6523802

[43,] 0.6523802

[44,] 0.6523802

[45,] 0.6523802

[46,] 0.6523802

[47,] 0.6523802

[48,] 0.6523802

[49,] 0.6523802

[50,] 0.6523802

[51,] 0.6523802

[52,] 0.6523802

[[1]]$bC_L_scale

[,1] [,2] [,3]

[1,] 0.6631071 NA NA

[2,] 0.3944435 NA NA

[[1]]$rnd_effect_peak

[1] 0.4289013 0.4654187 0.3731704 0.2930591 0.5850010

[[1]]$r_nb_L

[1] 1.077702 1.328653 1.264790

[[2]]

[[2]]$sigma_juv

[1] 0.8058385 0.2510155

[[2]]$alpha_juv

[1] 0.5751332

[[2]]$beta_nao3

[1] 0.5915989

[[2]]$beta_L

[1] 0.9091858

[[2]]$bCj

[1] -0.1662734 -2.4470824

[[2]]$wj

[1] 0.5216721 0.4586363

[[2]]$logitp_obs

[1] 0.5794551 -0.4646736 -0.1658537 -0.8042357 -0.9798544 -0.8552297 -1.1016053 0.3940862 0.5901769 0.9499890 -0.1321482 -1.0366533 1.1525726

[14] 0.1776582 -0.6725324 NA NA NA NA NA NA NA NA NA NA NA

[27] NA NA NA NA NA NA NA NA NA NA NA NA NA

[40] NA NA NA NA NA NA NA NA NA NA NA NA

[[2]]$beta_yr

[1] 0.379331

[[2]]$sigma_oLTD

[1] 0.3084695

[[2]]$alpha_LtD

[1] 1.10547

[[2]]$sigma_pLTD

[1] 0.543547

[[2]]$beta_juv

[1] 1

[[2]]$dd_LtD

[1] 0.2879817

[[2]]$beta_o_east

[1] -0.7857295

[[2]]$state_ltd

[1] 142856 143426 144168 143411 143355 143692 143399 144879 143696 144127 143535 143958 143834 143587 143734 143684 143714 144017 144095 144446 143818

[22] 143598 143933 143894 143500 144108 143917 143806 144222 143230 144082 144074 143492 143522 143631 142706 144150 143997 143719 142699 143038 144085

[43] 143386 143532 143235 143362 143710

[[2]]$n

[1] NA NA NA 11.24603 NA NA NA 12.61030 NA NA NA NA NA NA NA NA

[17] NA NA NA NA NA NA NA NA NA NA NA NA NA NA NA NA

[33] NA NA NA NA NA NA NA NA NA NA NA NA NA NA NA

[[2]]$beta_din

[1] 0.2698039

[[2]]$wD

[1] 0.5459826

[[2]]$pool

[,1] [,2]

[1,] -4.098293 -1.477728

[2,] -4.098293 -1.477728

[3,] -4.098293 -1.477728

[4,] NA NA

[5,] NA NA

[6,] NA NA

[7,] NA NA

[8,] NA NA

[9,] NA NA

[10,] NA NA

[11,] NA NA

[12,] NA NA

[13,] NA NA

[14,] NA NA

[15,] NA NA

[16,] NA NA

[17,] NA NA

[18,] NA NA

[19,] NA NA

[20,] NA NA

[21,] NA NA

[22,] NA NA

[23,] NA NA

[24,] NA NA

[25,] NA NA

[26,] NA NA

[27,] NA NA

[28,] NA NA

[29,] NA NA

[30,] NA NA

[31,] NA NA

[32,] NA NA

[33,] NA NA

[34,] NA NA

[35,] NA NA

[36,] NA NA

[37,] NA NA

[38,] NA NA

[39,] NA NA

[40,] NA NA

[41,] NA NA

[42,] NA NA

[43,] NA NA

[44,] NA NA

[45,] NA NA

[46,] NA NA

[47,] NA NA

[48,] NA NA

[49,] NA NA

[50,] NA NA

[[2]]$alpha_L_sc

[1] 0.9565470 0.0314358 -0.9715166

[[2]]$chi_L

[1] -0.1627568

[[2]]$wL

[1] 0.5595016 0.4410313

[[2]]$sigma_L_o

[1] 5.976851e-05 6.934664e-05 5.275779e-05

[[2]]$sigma_L_rnd

[1] 6.135404e-05 9.942219e-05 5.504120e-05

[[2]]$L_st

[,1] [,2] [,3]

[1,] 7 9 8

[2,] 7 8 8

[3,] 7 9 9

[4,] 10 10 11

[5,] 7 10 8

[6,] 11 7 9

[7,] 10 11 8

[8,] 9 8 9

[9,] 9 9 6

[10,] 10 10 10

[11,] 9 10 8

[12,] 9 10 9

[13,] 9 9 8

[14,] 8 8 9

[15,] 10 11 8

[16,] 8 9 10

[17,] 9 10 10

[18,] 8 10 10

[19,] 10 10 10

[20,] 9 10 5

[21,] 8 10 9

[22,] 9 8 10

[23,] 9 9 9

[24,] 8 10 11

[25,] 10 10 10

[26,] 10 8 8

[27,] 9 9 10

[28,] 10 8 10

[29,] 11 10 8

[30,] 8 9 9

[31,] 11 10 9

[32,] 9 10 11

[33,] 10 8 8

[34,] 10 8 8

[35,] 10 8 8

[36,] 9 9 9

[37,] 10 10 8

[38,] 7 9 9

[39,] 10 14 10

[40,] 10 9 9

[41,] 9 9 8

[42,] 8 10 9

[43,] 9 8 8

[44,] 9 10 10

[45,] 10 7 11

[46,] 9 11 9

[47,] 8 10 9

[48,] 11 10 8

[49,] 8 11 7

[50,] 10 9 8

[51,] 9 10 8

[52,] 9 10 9

[53,] 10 8 9

[[2]]$rnd_L

[,1] [,2] [,3]

[1,] -1.68193084 0.766195944 0.16344757

[2,] -0.30318822 -0.104313131 0.87642181

[3,] 0.98632772 -0.136259416 0.12769032

[4,] -0.47978419 -0.002373026 -0.20670013

[5,] 1.17437438 0.952317831 0.41458845

[6,] 1.16986807 -0.578393782 0.20957420

[7,] -0.26580029 -0.285800578 -0.13253357

[8,] -0.19445678 0.955885812 -1.23426599

[9,] 0.64021133 -0.247395816 0.68884107

[10,] -0.59267804 0.298090156 0.31411459

[11,] 2.04491079 0.204985026 -1.63590718

[12,] 0.16354973 0.614323202 0.44102694

[13,] -0.78174034 0.048013334 -0.45593646

[14,] -0.30758000 -0.311290052 0.63443445

[15,] 2.14441993 1.712645082 -2.33620218

[16,] -0.30210580 2.731178855 1.00528500

[17,] -0.50378546 -0.150875488 -0.31619129

[18,] 1.13736256 -1.589421277 0.15261823

[19,] -1.72480675 -1.797481015 2.25107225

[20,] 1.82746441 0.041140055 -1.34074799

[21,] -1.09590244 1.253653662 -1.41202611

[22,] 0.52393075 -0.441136230 0.12378729

[23,] -0.06322628 -1.928301616 -0.40064689

[24,] -1.72669922 -0.832252539 0.09802083

[25,] 1.18243707 -0.438554930 0.64523739

[26,] 0.27868708 1.722111958 1.37057193

[27,] -1.04679286 0.967566480 0.53923349

[28,] -0.23369056 -1.333673860 -0.54812276

[29,] -1.39191223 0.614599181 0.21129246

[30,] -0.84269837 0.724448992 -1.40243680

[31,] -0.68505137 1.716479177 -0.91062211

[32,] 0.51687246 -0.919736048 -0.82803194

[33,] 0.22898705 0.488092003 1.46308058

[34,] -0.49812708 -0.865647348 0.52569077

[35,] 0.18300445 -0.099463504 0.09620462

[36,] -0.52791652 0.109267962 0.35110124

[37,] 0.77914891 -0.167234999 0.88944185

[38,] 1.59303271 -0.180113818 -1.63645244

[39,] 0.19721220 -0.030202506 -0.48227022

[40,] 0.46467742 -0.206811601 -0.76022625

[41,] 0.74284684 0.126418247 -0.67545714

[42,] -0.53830751 -1.650017737 0.21331780

[43,] -1.09851409 0.324407539 -0.42785896

[44,] -0.40572345 -0.389961062 1.03287116

[45,] 0.02681788 -0.787232574 0.07717440

[46,] -0.83601586 1.057586106 -0.82445388

[47,] 0.53208620 0.787864100 0.75571008

[48,] 0.81961850 -0.776487220 -0.14968728

[49,] -1.02081187 -1.107785554 1.78156535

[50,] 1.58850379 0.580005121 -0.54609200

[51,] 0.54656919 -0.556172753 -1.19642224

[52,] -0.45554063 -0.861696707 0.87878610

[53,] -2.38744620 -0.290657131 -0.17441517

[[2]]$lemmings

[,1] [,2] [,3]

[1,] NA 0.6418015 0.7384702

[2,] NA 0.6203342 0.8452280

[3,] NA 0.6158513 1.0283439

[4,] NA 2.0405100 1.6561590

[5,] NA 1.2508658 2.8755305

[6,] NA 0.6440219 0.1722810

[7,] NA 0.1851505 0.8397796

[8,] NA 5.6257262 0.2107649

[9,] NA 1.4756331 0.8146250

[10,] NA 4.8050372 1.2174628

[11,] NA 0.4127117 0.2269534

[12,] NA 2.0401681 0.5074902

[13,] NA 0.9783595 0.2865724

[14,] NA 0.7167812 0.3938452

[15,] NA 0.1874984 1.9110357

[16,] NA 0.3371348 0.0377962

[17,] NA 0.6196287 1.8212993

[18,] NA 1.2854785 0.1084576

[19,] NA 0.4650499 1.6005409

[20,] NA 0.7279845 0.2653538

[21,] NA 0.9500247 1.5588936

[22,] NA 1.2861714 1.2593144

[23,] NA 0.5176184 3.1376826

[24,] NA 0.6204886 4.5948544

[25,] NA 3.8409607 0.9544011

[26,] NA 1.3996787 NA

[27,] NA 0.7878041 NA

[28,] NA 2.0286717 NA

[29,] NA 0.5701282 NA

[30,] NA NA NA

[31,] NA NA NA

[32,] NA NA 4.3370876

[33,] NA NA 0.1725318

[34,] NA NA 0.4542588

[35,] NA NA 1.4629337

[36,] NA NA 1.6515236

[37,] NA NA 0.7634362

[38,] NA NA NA

[39,] NA NA 1.9768472

[40,] NA NA NA

[41,] NA NA NA

[42,] NA NA NA

[43,] NA NA NA

[44,] NA 2.2673943 NA

[45,] 2.7541638 0.4910921 0.5145180

[46,] 2.3781863 NA 1.3963814

[47,] 0.8871775 NA 1.4394759

[48,] 0.4857033 NA 2.0679347

[49,] 2.1118764 1.5943822 1.7832247

[50,] 1.5472034 NA 0.3984208

[51,] 0.4218719 NA 4.1714029

[52,] 1.1199236 NA 1.6985413

[53,] 0.3974329 NA 16.6126004

[[2]]$tau0

[,1]

[1,] 0.8134732

[2,] 0.8134732

[3,] 0.8134732

[4,] 0.8134732

[5,] 0.8134732

[6,] 0.8134732

[7,] 0.8134732

[8,] 0.8134732

[9,] 0.8134732

[10,] 0.8134732

[11,] 0.8134732

[12,] 0.8134732

[13,] 0.8134732

[14,] 0.8134732

[15,] 0.8134732

[16,] 0.8134732

[17,] 0.8134732

[18,] 0.8134732

[19,] 0.8134732

[20,] 0.8134732

[21,] 0.8134732

[22,] 0.8134732

[23,] 0.8134732

[24,] 0.8134732

[25,] 0.8134732

[26,] 0.8134732

[27,] 0.8134732

[28,] 0.8134732

[29,] 0.8134732

[30,] 0.8134732

[31,] 0.8134732

[32,] 0.8134732

[33,] 0.8134732

[34,] 0.8134732

[35,] 0.8134732

[36,] 0.8134732

[37,] 0.8134732

[38,] 0.8134732

[39,] 0.8134732

[40,] 0.8134732

[41,] 0.8134732

[42,] 0.8134732

[43,] 0.8134732

[44,] 0.8134732

[45,] 0.8134732

[46,] 0.8134732

[47,] 0.8134732

[48,] 0.8134732

[49,] 0.8134732

[50,] 0.8134732

[51,] 0.8134732

[52,] 0.8134732

[[2]]$bC_L_scale

[,1] [,2] [,3]

[1,] 0.8836368 NA NA

[2,] 0.9123991 NA NA

[[2]]$rnd_effect_peak

[1] 0.664562595 0.002251966 0.938945883 0.707343789 0.772592752

[[2]]$r_nb_L

[1] 1.214734 1.082709 1.144162
