# Supplementary material for: Large-scale changes in marine and terrestrial environments drive the population dynamics of long-tailed ducks breeding in Siberia
Source: Sci Rep. 2022 Jul 19;12:12355. doi: 10.1038/s41598-022-16166-7 (PMC9296647; doi:10.1038/s41598-022-16166-7)
Supplement: Supplementary file 1 — Supplementary Information. [file 41598_2022_16166_MOESM1_ESM.zip › inits_mus.docx]

[[1]]

[[1]]$chi

[1] 0.5208115

[[1]]$sigma.st

[1] 0.06661859

[[1]]$sigma_obs

[1] 0.04096936 0.16735829

[[1]]$mus2

[,1] [,2]

[1,] NA 9.082381

[2,] NA 9.376530

[3,] NA 8.980414

[4,] NA 9.648732

[5,] NA 9.377974

[6,] NA 10.692875

[7,] NA 8.983335

[8,] NA 9.635350

[9,] NA 9.319421

[10,] NA 10.672512

[11,] NA 10.207485

[12,] NA 9.844469

[13,] NA NA

[14,] NA NA

[15,] NA NA

[16,] NA NA

[17,] NA NA

[18,] NA NA

[19,] NA NA

[20,] NA NA

[21,] NA NA

[22,] NA NA

[23,] 10.263560 NA

[24,] 9.131114 NA

[25,] 10.048408 NA

[26,] 10.463420 NA

[27,] 10.000449 NA

[28,] 10.410052 NA

[29,] 10.896266 NA

[30,] 11.162674 NA

[31,] 9.590278 10.511594

[32,] NA 9.755986

[[2]]

[[2]]$chi

[1] 0.3703607

[[2]]$sigma.st

[1] 0.907702

[[2]]$sigma_obs

[1] 0.5424022 0.2071932

[[2]]$mus2

[,1] [,2]

[1,] NA 8.367752

[2,] NA 10.124080

[3,] NA 10.509559

[4,] NA 9.800650

[5,] NA 9.624851

[6,] NA 9.619355

[7,] NA 9.604351

[8,] NA 9.302175

[9,] NA 9.792982

[10,] NA 10.337575

[11,] NA 10.024127

[12,] NA 9.610271

[13,] NA NA

[14,] NA NA

[15,] NA NA

[16,] NA NA

[17,] NA NA

[18,] NA NA

[19,] NA NA

[20,] NA NA

[21,] NA NA

[22,] NA NA

[23,] 10.774634 NA

[24,] 10.665538 NA

[25,] 9.902376 NA

[26,] 9.639397 NA

[27,] 10.626121 NA

[28,] 10.557077 NA

[29,] 9.802886 NA

[30,] 9.436340 NA

[31,] 10.689855 8.723442

[32,] NA 9.571211
