# Supplementary material for: Large-scale changes in marine and terrestrial environments drive the population dynamics of long-tailed ducks breeding in Siberia
Source: Sci Rep. 2022 Jul 19;12:12355. doi: 10.1038/s41598-022-16166-7 (PMC9296647; doi:10.1038/s41598-022-16166-7)
Supplement: Supplementary file 1 — Supplementary Information. [file 41598_2022_16166_MOESM1_ESM.zip › suppl_information_2022_06_23.docx]

Supplementary Information

Title: Large-scale changes in marine and terrestrial environments drive the population dynamics of long-tailed ducks breeding in Siberia

Authors: J. Rintala, M. Hario, K. Laursen, and A. P. Møller

Contents

[Supplementary methods 3](#_Toc106913819)

[Wind variables for long-tailed duck observation model 3](#_Toc106913820)

[Hierarchical modeling and statistical analyses 6](#_Toc106913821)

[Long-tailed duck spring counts 7](#_Toc106913822)

[Juvenile proportions of long-tailed ducks 9](#_Toc106913823)

[Fertilizer effect on dissolved nitrogen and phosphorus 11](#_Toc106913824)

[Lemming abundances 14](#_Toc106913825)

[The Wadden Sea mussel populations 19](#_Toc106913826)

[Summary of hierarchical models 21](#_Toc106913827)

[Model estimation 22](#_Toc106913828)

[References 28](#_Toc106913829)

[Supplementary tables 30](#_Toc106913830)

[**Supplementary Table S1a**:Lemmings 30](#_Toc106913831)

[**Supplementary Table S1b**:Juvenile proportions 32](#_Toc106913832)

[**Supplementary Table S1c**:Long-tailed duck spring counts 33](#_Toc106913833)

[**Supplementary Table S2**:DIN and DIP 35](#_Toc106913834)

[**Supplementary Table S3a**:Mussel biomass 38](#_Toc106913835)

[**Supplementary Table S3b**: Mussel flesh/shell ratio 40](#_Toc106913836)

[Supplementary Methods: Program codes 41](#_Toc106913837)

[BUGS code for the hierarchical model on the long-tailed duck spring counts, juvenile proportions, and lemmings 41](#_Toc106913838)

[BUGS code for the log model on DIN and DIP amounts in the Danish Straits and Baltic Proper 52](#_Toc106913839)

[BUGS code for the hierarchical model on the blue mussel biomasses 56](#_Toc106913840)

[BUGS code for the hierarchical model on the blue mussel flesh/shell ratios 59](#_Toc106913841)

# Supplementary methods

## Wind variables for long-tailed duck observation model

One of the challenges in the estimation of population dynamics based on annual spring migration counts was attributed to the highly varying counts being partly due to varying wind conditions during annual surveys. Møller 1 shows changes in wind over time. We implemented the wind direction and velocity variables into the population model as an observation error process, which was assumed to improve the precision of long-tailed duck population size estimates and the confidence of environmental parameters affecting the population estimates. We developed a diurnal weight factor for a potential wind effect on spring migration in long-tailed ducks. We did so with the following calculation:

| looping through years and days (S1)

In Supplementary Equation (S1) is the expected number of migrating long-tailed ducks per day based on pooled data from all years (see below). is daily wind velocity in year . Each annual and daily wind velocity value is multiplied with corresponding daily value . Wind direction variables were used as 0 to 2π radians (increasing clockwise) (Supplementary Fig. S1). Annual wind-effect is the weighted mean from daily scores for the north-south aspect as:

, (S2)

and for the east-west aspect as:

. (S3)

The vector comprise daily weights for year , and correspondently radians is the vector of daily wind directions.


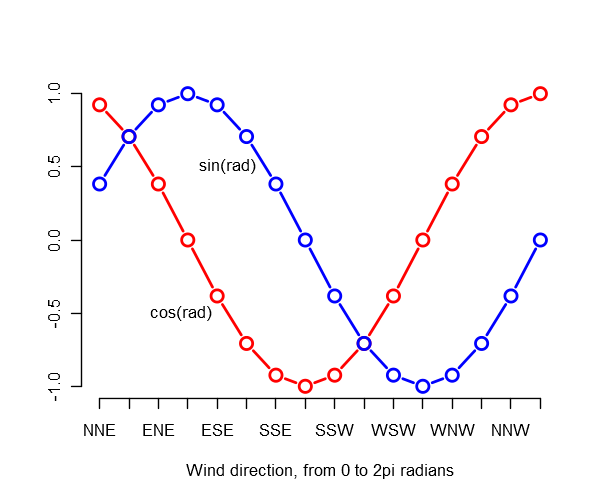


**Supplementary Fig. S1**. Wind direction variables as 0 to 2π radians reflecting North-South (red) and East-West (blue) aspects.

Calculation with Supplementary Equation (S1) resulted in a frequency distribution of pooled daily numbers of long-tailed ducks (Supplementary Fig. S2).


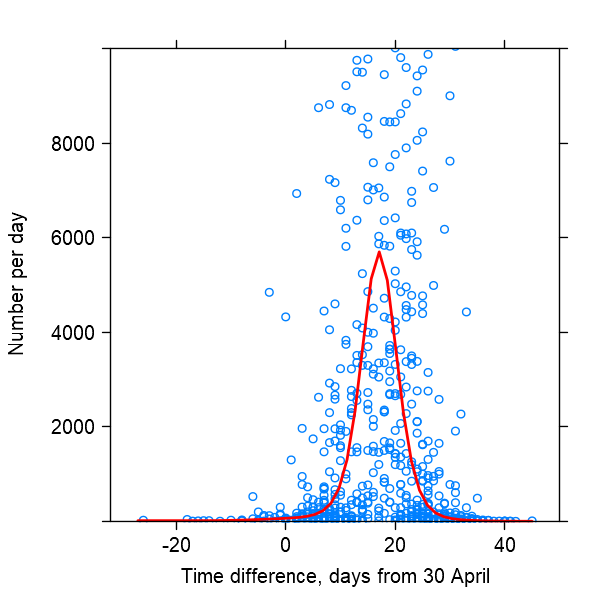


**Supplementary Fig. S2**. The number of long-tailed ducks at Söderskär, Gulf of Finland, during spring migration after adjustment for daily scores. 0 refers to April 30.

Based on a generalized additive model (GAM) 2,3 on the observed daily counts of long-tailed ducks, we projected the smoothed distribution of counts relative to April 30 (Supplementary Fig. S3).


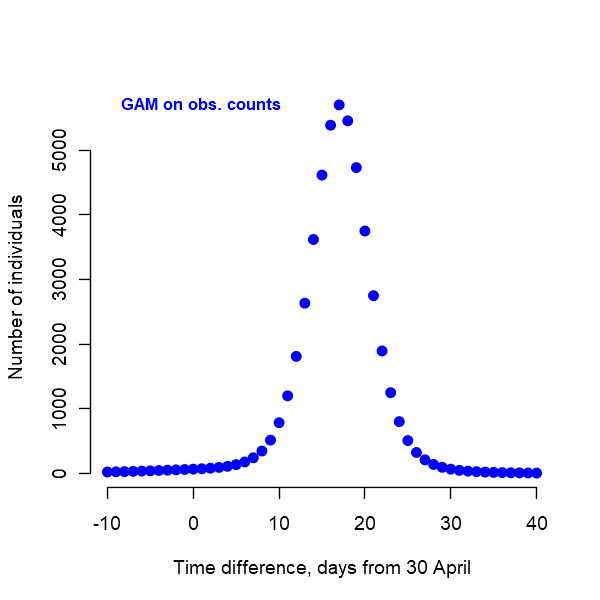


**Supplementary Fig. S3**. Expected daily numbers of migrating long-tailed ducks, in Supplementary Equation (S1), during spring migration according to GAM analysis based on observed counts. 0 refers to April 30.

Annual weighted mean scores based on Supplementary Equations (S2) and (S3) for diurnal wind direction and velocity were calculated for north-south and east-west aspect winds (Supplementary Fig. S4).

We did not have digitized wind data specific for Söderskär in 2008–2010. For this period, we used wind observations from the nearby weather observatory Eestiluoto located at ca. 10 km to west-northwest from Söderskär. We accessed these data (Finnish Meteorological Institute) by using the *R* package ‘fmir’ (<https://github.com/mikmart/fmir>). Preliminary models indicated that the east-west aspect (Supplementary Fig. S4) is relevant in the long-tailed duck observation model, and we retained this in the final formulation for the wind effect. The performance of the wind variable in the hierarchical model on long-tailed ducks, Supplementary Equations (S4a) – (S4d), is clarified with the explanation for the observation model, Supplementary Equation (S5), below.


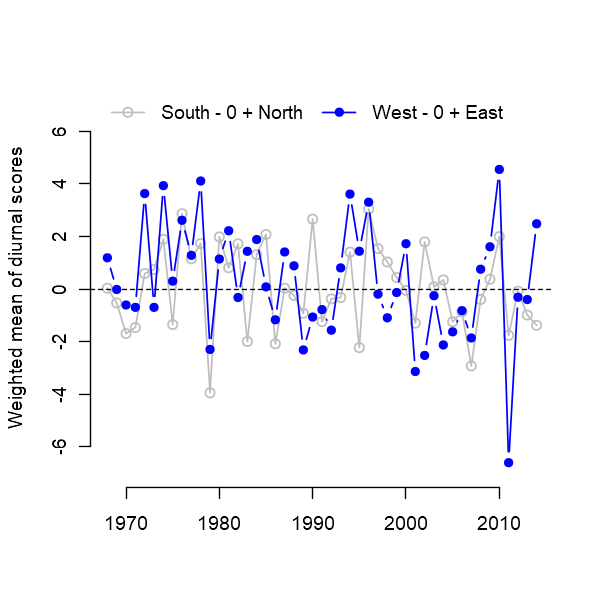


**Supplementary Fig. S4**. Weighted means for annual wind effects by Supplementary Equation (S3) from diurnal wind scores expressing direction and velocity for North-South and East-West aspects of wind.

## Hierarchical modeling and statistical analyses

We used an autoregressive state-space model 4, which is an integrated hierarchical model 5 with Bayesian MCMC posterior sampling 6,7 for the long-tailed duck. As sub-models, functioning dynamically under the main model for long-tailed ducks, we included a logit-model for juvenile proportions (supposed to describe the recruitment of young into winter population) based on Danish wing samples 8 and a state-space model for log lemming dynamics (assumed link to predation pressure at the breeding grounds) in the Western Taimyr Peninsula. We built a model to quantify the link between nutrient runoff from farmland areas and the Baltic Sea nutrient pools (assumed effect on mussel abundances and hence also long-tailed ducks) based on fertilizers applied in Danish farmland 9,10 and dissolved nitrogen (DIN) and phosphorus (DIP) amounts in the Danish Straits and Baltic Proper 11. A model was constructed for the Wadden Sea blue mussels *Mytilus edulis* to study the effects of nutrient availability on mussel population dynamics and another model for flesh contents in mussels. In each model, explanatory environmental variables were log-transformed (if possible).

### Long-tailed duck spring counts

The main model expressing long-tailed duck dynamics (1968–2014) is written as:

(S4a) (S4b)
 (S4c)
 (S4d)

that formulates log state population dynamics based on yearly spring migration counts, , of long-tailed ducks. State process errors, or dispersion from predicted population size , were modeled based on negative binomial distribution 6,12 that was needed to control for over-dispersed error pattern. , for variable (> 1), quantifies the (multiplicative) effect of juvenile proportion in the autumn (year ) and winter (years and ), based on Supplementary Equations (S6), (S7), (S8), and (S9), on migration counts in the next spring. Dissolved nitrogen and phosphorus amounts correspond to trajectories estimated from the nutrient amounts from two of the Baltic Sea basins, the Danish Straits and the Baltic Proper (output from Supplementary Equation (S10)). The nutrient series implemented into Supplementary Equation (S4a) were scaled to a mean of zero and unit variance. is a weighting parameter for nutrient effects. The intercept, , and are normally distributed with uninformative and weakly informative priors, respectively. and parameters are beta distributed varying between zero and one (uninformative prior beta-distribution with and , *i.e.* 13). A prior for density-dependent parameter was defined as , *i.e*., truncated normal distributions 14 returning values between 0 and 1.

**
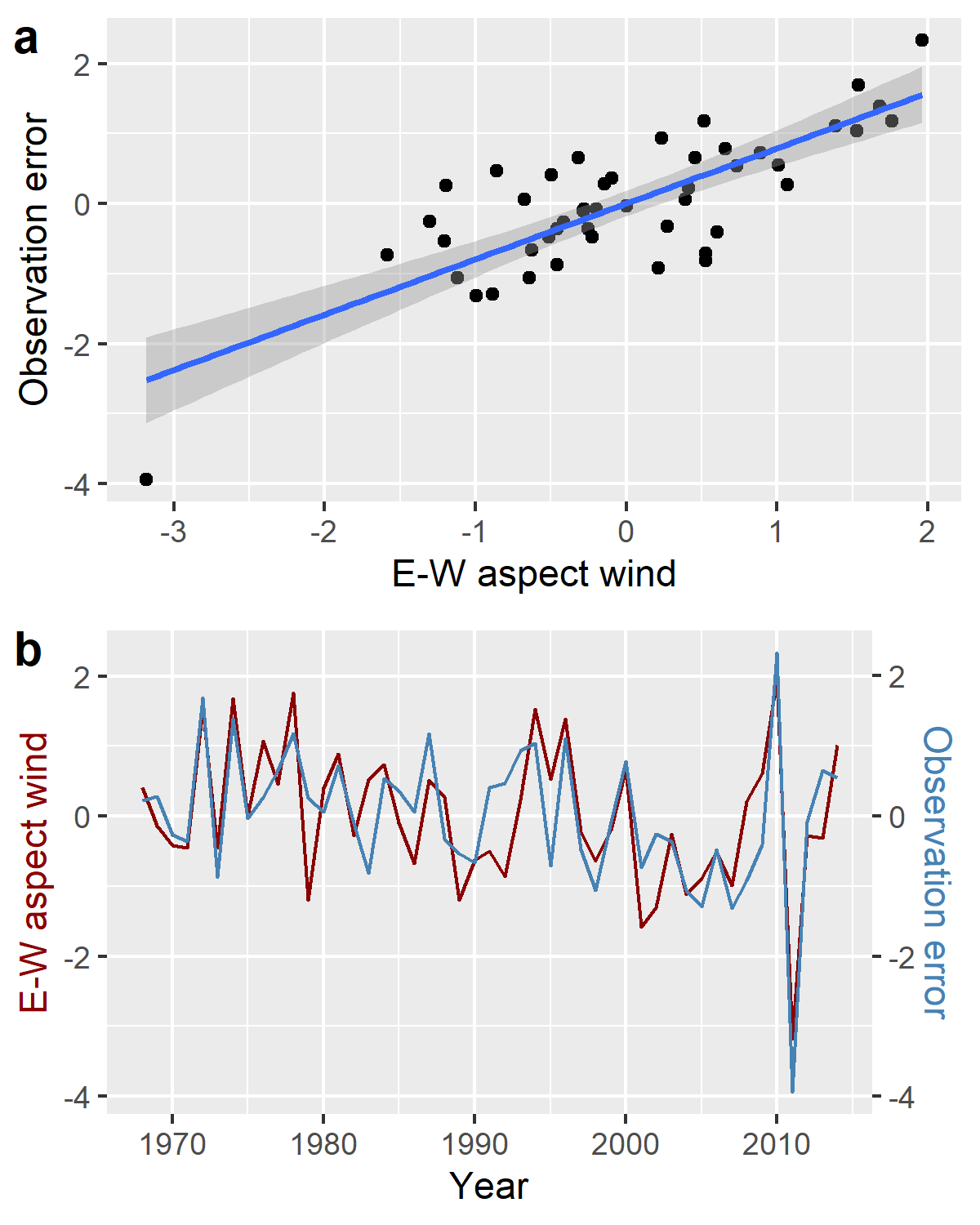
**

**Supplementary Fig. S5**. Observation errors in long-tailed duck spring migration numbers related to the strength of east-west aspect wind at the Söderskär bird observatory during 1968–2014. (**a**) Lower values along with the x-axis indicate more common and stronger western winds. (**b**) The annual variation is shown for the wind scores and observation error (values scaled to mean zero and unit variance).

Environmental disturbances are assumed to have normally distributed random errors around zero-vector that are defined as, in which the elements correspond to annual responses to unspecified environmental disturbances. A hyper-prior for is defined as 5. The same method for setting hyper-priors applies also to the normally distributed error terms of models below.

An observation model for long-tailed ducks, linked to Supplementary Equations (S4a) – (S4d), is specified with Gaussian errors and *a priori* assumed error effects (wind) as:

(S5)

Let represent annual observed counts also containing measurement errors, and taking the natural logarithm, , gives us the normally distributed relation, where is a parameter for east-west aspect winds (Supplementary Fig. S4), and ** ** is of the random observation-error process (with a prior set as for above, but varying between 0 and 10), which controls for unspecified annual measurement errors.

The observation process variance (*i.e.*, sampling error or observation error) of spring migration counts was affected by east-west aspect winds so that observed numbers declined with strengthening western winds during the annual census period. The observation process variation followed long-term trends and extreme annual deviations in east-west aspect winds. The parameter was highly confident and explained 51% of the total observation error variance. The structure of observation errors concerning east-west aspect winds based on Supplementary Equation (S5) is visualized in Supplementary Fig. S5.

### Juvenile proportions of long-tailed ducks

During 1982–2017 in total 3,559 wings of long-tailed ducks were collected by hunters in the Danish wing survey. The annual mean (*SE*) number of wings was 98.9 (8.7) wings. The mean (*SE*) reproduction, measured as the ratio of the number of juveniles to the number of adult females, was 0.864 (0.124) juveniles per female with the range: 0.031 – 2.892 15. The hunting season was shortened by one month from 2011 (from October 1 – February 29 to October 1 – January 31) which had no clear effect for the hunting bag in 2011-2017 16. We constructed a logit model to estimate environmental effects on the long-tailed duck juvenile proportions and extended the estimates for the period 1967–2017:

(S6)
 (S7)
 (S8)
 (S9)

in which is the output of a generalized linear model (logistic regression). Here, is the expected proportion of juveniles, and stands for the observed annual proportion. The multiplicative estimate for juvenile proportion is functioning in the main model in Supplementary Equation (S4). series are estimates for lemming population sizes in the previous year based on the longest series of lemmings (Kara Sea, ) from Supplementary Equation (S12). is an estimate for winter climate variability (NAOI Dec–Feb) three years before. terms quantify the effects of precipitation and temperature in North-western Siberia in May and June on juvenile proportions. For instance, precipitation was weighted as (for the weighting parameter, , cf. Equation S4a). Constants and are assumed to have a normal distribution. Random environmental disturbances are defined as with a hyper-prior for the standard deviation set as . Normally distributed residuals for the model come from the link described in Supplementary Equation (S7), where . Some of the parameters in the sub-model for juveniles, Supplementary Equation (S6), did not converge well with uninformative priors when integrated into the main model by Supplementary Equations (S4a) – (S4d). We solved this problem by setting weakly informative priors for the constants , and , *i.e.*, informative means and less vague variances assumed for prior distributions based on a preliminary run, which is often advisable with integrated population models 5.

### Fertilizer effect on dissolved nitrogen and phosphorus

The total amount of fertilizer (tons) used in Danish farmland was estimated during 1920–1998 10 and the total amount of nitrogen (tons) in fertilizer during 1990–2016 9. The two data sets were calibrated by estimating the mean ratio of the two data sets during the overlap period (1990–1998, the correlation between the two data sets was *r* = 0.98, *N* = 9), and multiplying the values by the mean ratio from Blicher-Mathiesen et al. 9.

Data on nitrogen leaching into a representative number of rivers were available from 1965–2002 17. There was a strong positive relationship between the level of leaching and the amount of fertilizer applied to farmland in different years (likelihood ratio χ2 = 82.32, df = 1, *r*2 = 0.61, *p* < 0.0001, estimate (*SE*) = 0.408 (0.035)). Leaching of nitrogen via rivers to the Danish marine waters was estimated for the period 1965–2015 by combining data of the annual leaching of the total amount of nitrogen (tons) in a representative number of rivers to coastal waters during 1965–2002 18 and data for 1991–2015 of the estimated loss of total nitrogen (kg N ha–1) to rivers from a representative number of farmland areas 17. The mean ratio between the two data sets was calculated for the overlapping period (1991–2002) and the data for the period 1991–2015 was multiplied by the mean ratio. The correlation between the two data sets for the overlapping period was: *r* = 0.64 (*N* = 12).

A log scale state-space model for fertilizer effect on dissolved nitrogen (DIN) and phosphorus (DIP) amounts in the southern Baltic Sea, returning state estimates for DIN and DIP for years 1970–2016, is written as:

(S10)

where parameters are intercepts, and controls for fertilizer-use 9,10 () effect on state-processes describing combined (latent) nutrient trajectories in the southern Baltic Sea. Non-linear effects are specified with a function for fertilizers, generating smoothing variables for DIN and DIP: . matrix comprise five non-linear thin-plate splines 19 (Supplementary Fig. S6) and vectors comprise parameters controlling for the shape of the two additive smoothers estimated respectively for DIN () and DIP (). Parameters , , and have uninformative normally distributed priors.

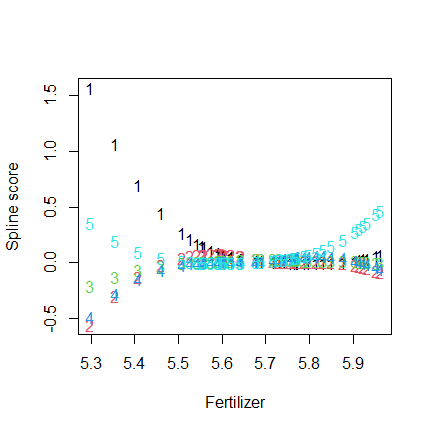


**Supplementary Fig. S6**. Thin-plate splines (1–5) based on annual amounts of fertilizers used in Danish farmland (log tons) were applied to estimate the state-space trajectories of dissolved nitrogen (DIN) and phosphorus (DIP) according to Supplementary Equation (S10).

Serial correlation is controlled for by , *i.e*. (weakly informative) normal distribution truncated 14 to return values greater than zero. Normally distributed is a random effect term, controlling for other processes assumed independent of fertilizer runoff affecting dissolved nutrient amounts in the southern Baltic Sea. A hyper-prior for the standard deviations of the disturbances were set as (Supplementary Equation (S10)). A possible lagged effect of fertilizers applied by Danish farmers on nutrient levels in the southern Baltic Sea was controlled for by parameter that is binomially distributed and can have values of 0, 1, 2 or 3 (years).

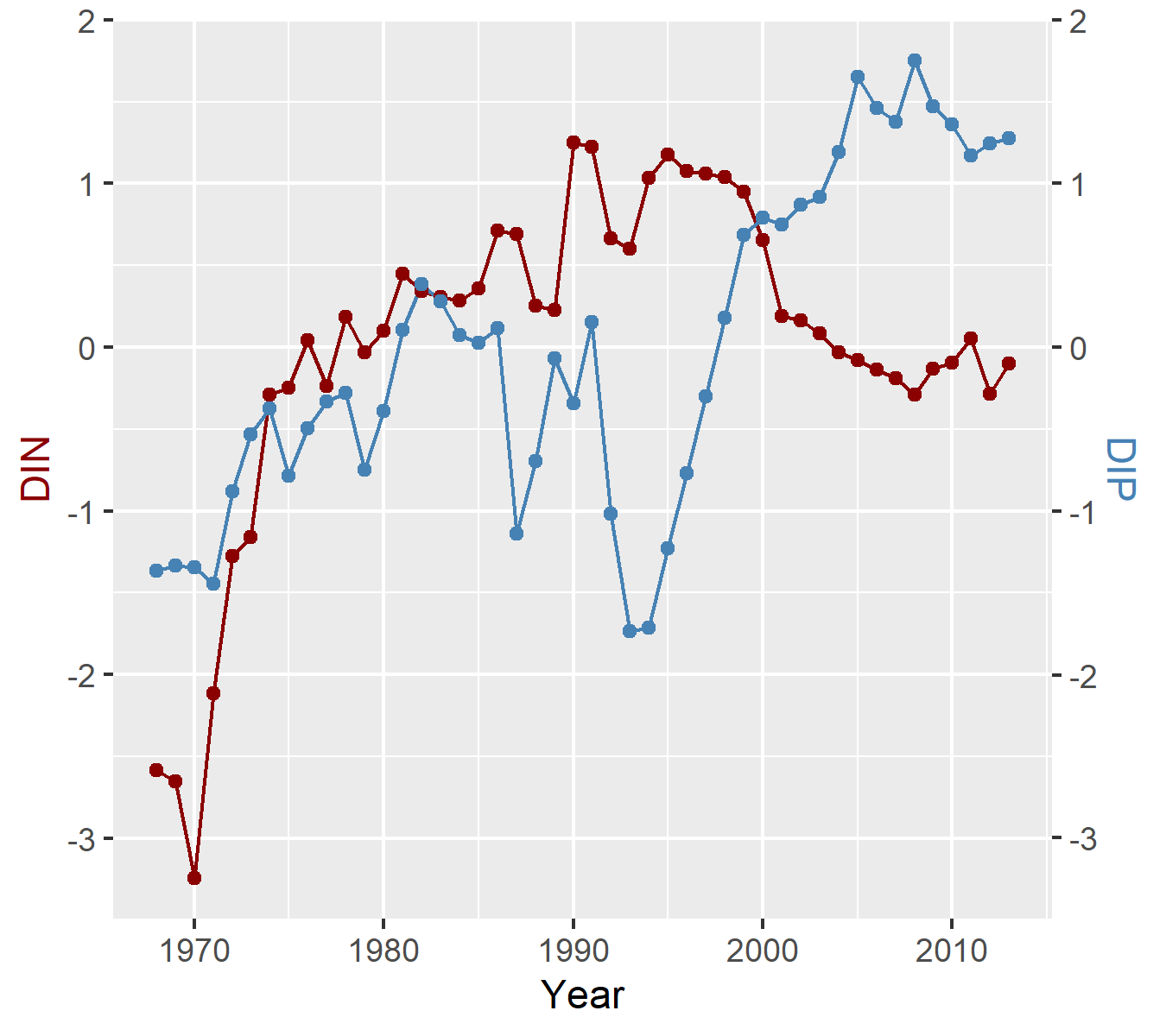


**Supplementary Fig. S7**. State trajectories of dissolved nitrogen (DIN) and phosphorus (DIP) in the southern Baltic Sea from Supplementary Equation (S10) were used as predictors for the long-tailed duck population size by Supplementary Equation (S4a). Each trajectory is scaled to mean one and unit variance.

Observed nutrient amounts were linked to the states via an observation model, Supplementary Equation (S11), with intercepts and error variances controlling for nutrient levels and variation around state trajectories, respectively. A hyper-prior for the standard deviation related to each nutrient series () and the Baltic Sea pools () was defined as . The intercepts and have uninformative normally distributed priors. The other intercepts are structural zeros, *i.e.*, . Hence, the observation model becomes as:

(S11)

The array , Supplementary Equations (S10) and (S11), comprising two state-processes, generalizing nutrient trajectories in the southern Baltic Sea (Supplementary Fig. S7), were transferred to the long-tailed duck spring counts model in Supplementary Equation (S4). The main model for the long-tailed duck with a parameterization for one-year lagged DIN and DIP effects, Supplementary Equation (S4a), describes population variations starting from 1968. Weakly informative priors for missing values in DIN and DIP in 1968–1969 were assumed and these were updated under the MCMC simulations under the main model (Supplementary Equation (S4a).

### Lemming abundances

In our analysis, the data by Kokorev & Kuksov (Kara Sea) 20,21 contain observations from the period 1965–2008 and the second ones 22 (survey no. 44, Meduza Bay) from the period 1994–2017. In these series, lemming abundances were indexed by ordinal (assumed logarithmic 20) numbers 1–5, classifying annual abundances into five categories according to the total number of lemmings caught per 100 trap-days (1: 0; 2: 1–3; 3: 4–10; 4: 11–30; 5: > 30) 21. To produce transformed series linearly corresponding to the original categorical values, we first assumed values and and regressed against to derive transformed series with the estimated function (Supplementary Fig. S8a). The second categorical lemming index series 22 (Meduza Bay) also range from 1 to 5 but include half-steps: . From these, we generated transformed series by substituting the second series to the above function: (Supplementary Fig. S8b). No such manipulations were needed for the third series 22 (survey no. 45, Mys Vostochnyi, from the period 1990–2008) that comprised lemming individuals caught per 100 trap nights. Only a transformation constant one was added to each year’s value allowing log-scale evaluation.


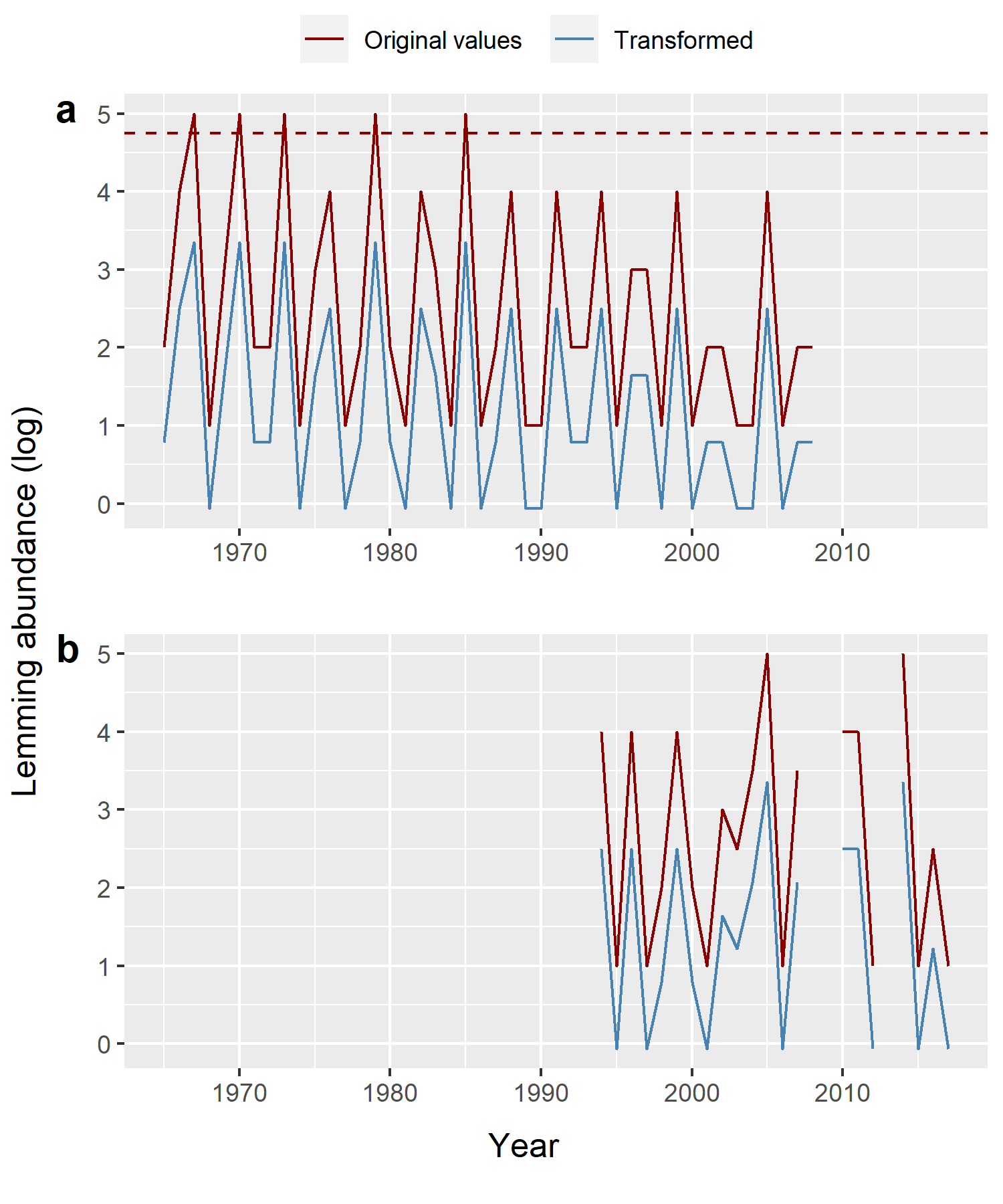


**Supplementary Fig. S8**. Original categorical values and transformed ones of two lemming populations (**a**, Kara Sea and **b**, Meduza Bay; Western Taimyr Peninsula). Abundances by “Original values” intersecting the dashed line indicating high population peaks acknowledged in the observation model according to the explanation given for Supplementary Equation (S13).

Logarithmic lemming dynamics based on the three separate population time-series (Kara Sea, Meduza Bay, and Mys Vostochnyi, respectively) of lemmings (catch-rate of individuals) in the Western Taimyr Peninsula (1966–2017) affecting juvenile proportions in Supplementary Equation (S6) was estimated based on the following negative binomial model:

(S12a)
 | (S12b)
 (S12c)
 (S12d)
 (S12e)
 (S13)

in which, in the right side of Supplementary Equation (S12a), if an observed count of population 1, , is zero or if the year is 2012, 2015 or 2017 (population 2, Supplementary Fig. S8b), where comes from the varying between 0 and 1. With the prior setting, the (independent) posterior beta-distribution mean is 0.01. We applied this to maximize the effect of lemming troughs on the population dynamics. During troughs, lemmings are so rare that it is likely that no individuals will be captured during a survey 21,23. The intercept and climate variables are normally distributed with weakly informative priors 5 based on a preliminary run (like for juvenile proportions above). Similarly, the density-dependent parameter is normally distributed with weakly informative priors, possible values varying between –1 and 1 sampled from truncated normal distributions 14. Weakly informative normally distributed priors were set also for additive scaling parameters for populations 2 and 3, and , as well as for and indicating additive climate effects on populations and . array comprises two Northwestern Taimyr climate variables indexed with (1 or 2) for each population . is weighted average precipitation (*P*) for two summer months: . is a weighting parameter varying between 0 and 1. Similarly, is weighted autumn precipitation ( for September and October in the previous year ). The weighting parameters are beta distributed with uninformative priors set as and . is a parameter for temporal effect depending on year ( referring to 1965). A term for random environmental disturbances has mean zero and variance with a hyper-prior set as . and are additive to . The upper limits for uniformly distributed standard deviations for observation error variances in Supplementary Equation (S13) were set as 1 (populations 1 and 2) and 0.01 (population 3). Due to the over-dispersed error structure of the population dynamics, we specified state population errors based on the negative binomial distribution 6,12 by Supplementary Equations (S12c) – (S12e).


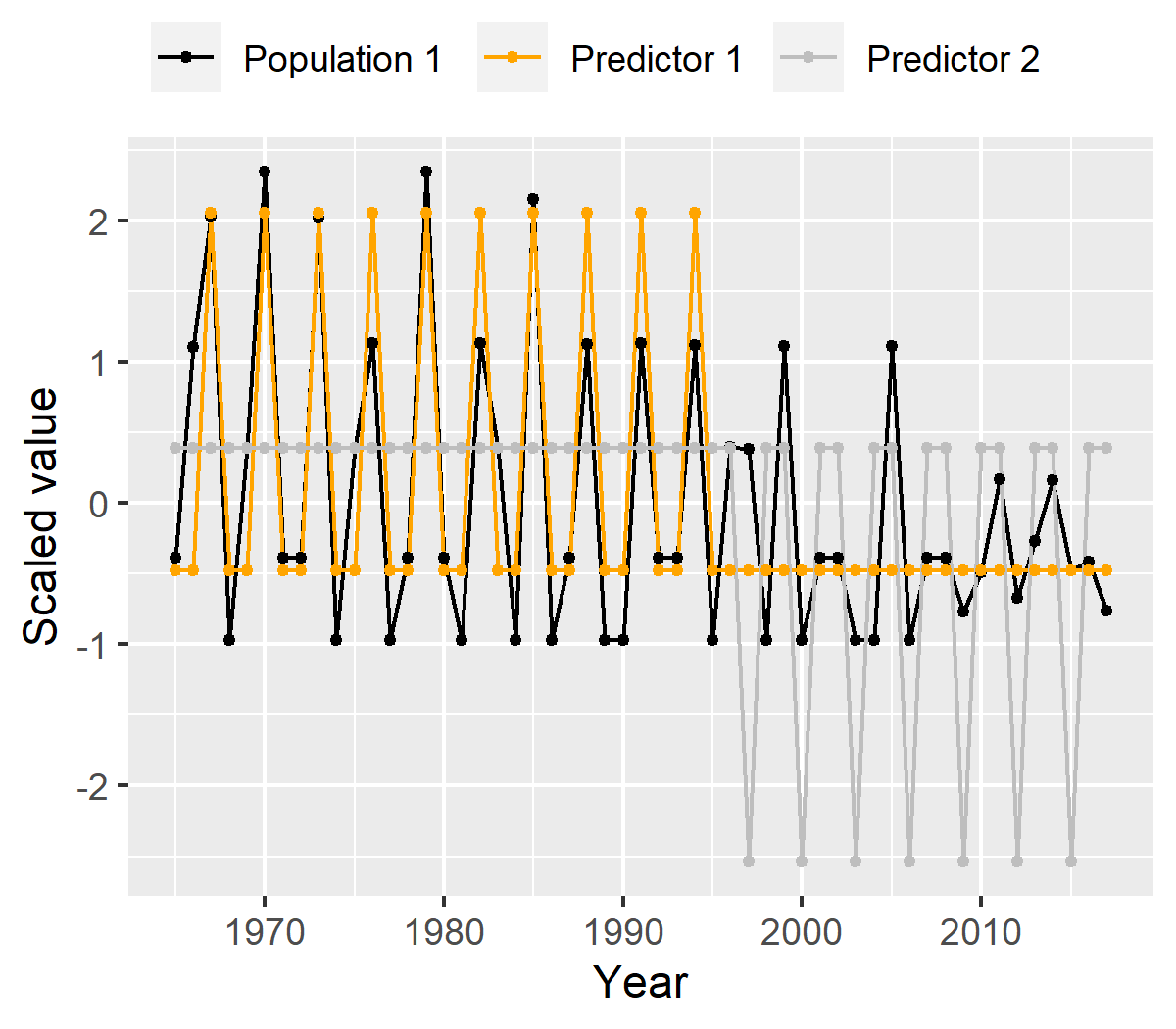


**Supplementary Fig. S9**. Estimated lemming population dynamics (Population 1, Kara Sea; Western Taimyr Peninsula) according to Supplementary Equations (S12) – (S13) with predictors based on multiplication with (Predictor 1 for 1965–1994) and (Predictor 2 for 1995–2017), allowing general positive or negative (peak or trough) effect on population size by three-year intervals. Each series is scaled to mean zero and unit variance.

Parameter vectors and quantify the consistency of three-year cyclic dynamics designed into dummy variable (0, 1) matrices and , in which the number one allows regular effects by three-year intervals as . Columns () from 1 to 10 in the first matrix indicate intervals from 1967 to 1994, and in the second one, seven columns indicate intervals from 1997 to 2017. We treated the three-year cyclic periodicity as *a priori* known characteristics of the lemming dynamics in the Western Taimyr Peninsula from the 1960s up to 1994 20. Thus, according to the previously known pattern, we built periodicity structure into the model with matrix × vector multiplication and producing two predictors: the first one for the period up to 1994 and the second one for the period starting from 1995. Supplementary Fig. S9 shows how the first predictor projects the peaks up to 1994. The second predictor indicates that the former regular dynamics break down and, instead of peaks, the second predictor co-varies rather with population troughs (Supplementary Fig. S9). To avoid overfitting, we constrained the predictors so that all coefficients in a parameter vector are equal, *e.g*., in **:** (Supplementary Table S1a).

In preliminary models, none of the climate variable combinations was sufficient to control for the sharp and regular population variations based on the categorical data structure (Kara Sea and Meduza Bay, Supplementary Fig. S8) and the parameters were unstable as indicated by insufficient convergence of simulation traces. We solved this problem by allowing observation error to vary more during peak years than otherwise. We did so by estimating an additive error parameter for observation error variances related to the peak years met in the longest series (Kara Sea, Supplementary Fig. S8a) as: , otherwise , with a prior according to . Here, the function I() generates truncated normal distributions, only returning values greater than zero. This treatment enabled controlling for an error (plausible underestimate) related to the highest catch-rate class (5: > 30).

### The Wadden Sea mussel populations

Blue mussels are the main food for long-tailed ducks. Winter conditions are assumed to affect mussel stocks and flesh content, and hence, reproduction in mussels. During cold winters flesh content is high due to reduced respiration and mussel stocks increase due to large spatfall, which are often initiated by the cold winter climate 24. In cold water, the survival of mussel larvae during settling is high due to lowered predation pressure, giving rise to large cohorts 1–3 years after a cold winter 24. Consequently, we suggested winter climate also affects juvenile proportions in long-tailed ducks by Supplementary Equation (S6). It is also known that the amount of fertilizer use increases nutrient levels and thus the growth of mussels 25. However, excessive amounts of nutrients may cause hypoxia and finally bottom death 26 and severe declines in mussel stocks with potential adverse effects to the body condition of wintering (female) long-tailed ducks, winter and spring migration survival and reproductive output. To estimate environmental effects on mussels, we built a hierarchical model based on two biomass surveys from the Wadden Sea during the period 1986–2017, assuming environmental variables as temperature and nutrients (exclusive events of hypoxia) have comparable effects on mussel population dynamics in the Wadden Sea and the southern Baltic Sea, excluding hypoxia due to nutrient overflow considering the Baltic Sea brackish water pools.

A state-space model for logarithmic biomasses on the Danish Wadden Sea for the period 1986–1997 (1st population, ) is written as:

(S14a)

where is intercept and is the density-dependent parameter, a prior restricted to vary uniformly between 0 and 1. is state biomass for the 1st population in year . State process errors due to environmental variance are defined as and a hyper-prior for the standard deviation as . is an error term for demographic variance 7,27, and its hyper-prior is defined as . A component is a matrix × vector function for five non-linear thin-plate splines 19 based on the fertilizer series as explained for Supplementary Equation (S10) (*cf.* Supplementary Fig. S6). A function defines the weighted effects of mean winter temperature (December (*t*–1) to February (*t*)), specific for the Wadden Sea coastline, on mussel biomasses two and three years later. varies between zero and one by beta distributions with uninformative priors ( and ). A time-lag in fertilizer effects on mussel growth is controlled for by parameter that is binomially distributed returning values 1, 2 or 3 (years) (*cf*. Supplementary Equation (S10).

Biomass estimates for Schleswig-Holstein () (2nd population) follows the parameterization given in Supplementary Equation (S5a), excepting an independent demographic error term and additive scaling parameter for an observation model. Hence, biomass estimates were linked to Supplementary Equation (S14a) as:

(S14b)

in which the parameter scales the 2nd population additively to the 1st population and is demographic error specified for the 2nd population with the same prior setting as for the 1st population above. The observed logarithmic mussel biomasses function in the observation model as: with hyper-priors for their standard deviations given with .

A large flesh content in mussels enables producing large spatfall (production of eggs), giving rise to large cohorts after two or three years 24. We tested the effect of fertilizers and temperatures on flesh contents, log flesh/shell ratio *i.e.*, (flesh proportion), with a model for the period 1998–2013:

(S15)

In the model, the logit proportion is predicted with parameters for winter and spring temperatures, and for fertilizer use maximally two years before functions as a proxy for nutrient surpluses 25. comes from a binomial distribution returning values 0, 1 or 2 (years) (with priors giving even-distributed frequencies as above). A hyper-prior for the standard deviation of random environmental disturbances was defined as . With the same definition for a hyper-prior, residual errors are normally distributed around zero vector (*cf*. Supplementary Equations (S7) – (S9)).

## Summary of hierarchical models

Understanding the system affecting the long-tailed duck dynamics required multiple hierarchical models with several time-series variables, with lagged effects adjusted based on species-specific responses to environmental variables. The main model for long-tailed duck spring counts involved sub-models for juvenile proportions and lemmings. Separate models were constructed for dissolved nutrients, mussel biomasses and flesh/shell ratios. The model for nutrients was built to demonstrate fertilizer effects on (latent) DIN and DIP trajectories in the southern Baltic Sea to be implemented into the main model for the long-tailed duck. Here, to avoid unnecessary complexity, these were treated as fixed variables (like climate variables) instead of assuming stochastic nutrient processes under the main model. The models with their relations and environmental variables with varying lagged effects are summarized in Supplementary Methods Table SM1.

## Model estimation

To evaluate the importance of different parameters affecting a certain process, we estimated the proportions of different variance components of the total process variance. For instance, the component due to density dependence in population dynamics is expressed as based on stationary population variance that converges as: provided that 28. Here, is environmental random error variance and , *e.g.,* in Supplementary Equation (S4), is the density-dependent parameter. Long-tailed duck and mussel population dynamics were clearly not at a stationary state because of rapid declining trends. For these, we ran preliminary analyses with fixed parameters controlling for density dependence and process errors (pre-estimated posterior means) and simulated stationary dynamics resulting in stationary random error variance to be used in the calculations of proportional variance due to density dependence based on the final models.

To sample from the joint posterior of the model parameters, we used Markov chain Monte Carlo (MCMC) simulations through Gibbs sampling 6,29 implemented with JAGS version 4.3.0 14. We used *R* version 4.0.2 30 and package ‘R2jags’ 31 for the preparation of datasets, running and summarizing the simulations. For the main model combining Supplementary Equations (S4a) – (S9) and (S12a) – (S13) for long-tailed duck spring counts, juveniles, and lemmings, sampling from the parameter posteriors was based on initialization of two simulation chains and a run of 400,000 iterations (discarding the first 200,000 samples of each chain as burn-in) and retaining every 400th update as the basis for posterior estimates. The same was applied to mussel biomasses, combining Supplementary Equations (S14a) and (S14b). For the flesh proportion model, Supplementary Equation (S15), 200,000 (100,000) iterations were run retaining every 100th iteration. For the nutrient model, Supplementary Equations (S10) – (S11), 80,000 (40,000) iterations were run. Convergences were fair based on inspection of Markov chains. values were observed converging at close to unity, which indicates good convergence 6. Satisfactory mixing of MCMC traces of all important parameters requires 6. Poor convergence of parameters and mixing of MCMC chains is a problem with integrated hierarchical models 5. In addition to the use of weakly informative priors, we used penalized thin-plate splines 19 for the DIN/DIP and mussel biomass models to improve the mixing properties of the MCMC chains. The program codes for the Bayesian analyses are shown below.

**Supplementary Methods Table SM1**.

| **Hierarchical model** | **Supplementary Equation** | **Response parameter** | **Predictor** | **Lagged effect, years** | **Explanation and hypothesis** |
| --- | --- | --- | --- | --- | --- |
| **Main model**: Long-tailed duck population dynamics | (S4a) | Long-tailed duck population size based on spring migration counts | Juvenile proportions from sub-model 1 | 1 | Wing samples of juveniles in autumn and winter date to the year preceding spring migration. Increases in the number of young per adult females should appear as increases in second calendar-year recruits to spring migrating population, bringing about increases in population size estimates as well |
| " | " | " | DIN + DIP (missing values filled with estimates from separate model 1) | 1 | The joint effect of these variables was estimated based on the weighting approach. Nutrient amounts supporting primary production in the preceding year of the spring migration are assumed to promote the winter survival and the condition of long-tailed ducks preparing for migration to the High Arctic. Adverse effects may arise from hypoxia via nutrient overload to the Baltic Sea |
| " | (S6) | **Sub-model 1**: juvenile proportions from Danish hunting bag | Lemmings from sub-model 2 | 1 | Lemming population crashes one year after peak years are connected to decreased survival of young in the breeding ground, which is reflected in juvenile proportions observed in the wintering ground in the next autumn |
| " | " | " | Winter NAOI | 3 | Cold winters stimulate mussel reproduction, and in the Southern Baltic, it takes ca. 3 years for an individual mussel in a new cohort to grow up at the optimal size preferred as food items by long-tailed ducks. Increases in mussel stocks of preferred size improve the female condition and, thus, surviving over winter and during spring migration to the breeding ground in the High Arctic, allowing maximizing productivity |
| " | " | " | Northwestern Siberian climate (precipitation and temperature in late spring and early summer) | 0 | Conditions during breeding affect the reproduction of long-tailed ducks with consequent effects on juvenile proportions observed in the wintering ground in the next autumn |
| " | (S12a) | **Sub-model 2**: lemming population size in the Western Taimyr Peninsula | Precipitation, summer (Western Taimyr) | 0 | Current climate conditions affect the survival and/or reproduction of lemmings |
| " | " | " | Precipitation, autumn (Western Taimyr) | 1 | Conditions in preceding autumn affect population states and reproductive potential, having consequences also to population size in the next year. Precipitation trends may reflect general climate change effects on lemming populations in the High Arctic |
| **Separate model 1**: Dissolved nitrate (DIN) and phosphate (DIP) in the Southern Baltic Sea | (S10a) | DIN and DIP | Fertilizers applied by Danish farmers | 1 | Based on the analysis, application of fertilizers realized as increased nutrient levels in the Southern Baltic Sea one year later |
| **Separate model 2**: Mussel biomasses on the Wadden Sea | (S14a), (S14b) | Mussel biomass | Temperatures along the Wadden Sea coastline (winter) | 2–3 | Joint effects for two and three-year lags were estimated. It has been hypothesized that cold winters support reproduction in mussels and consequent large mussel cohort up to 3 years later |
| “ | “ | “ | Fertilizer | 1–2 | One-to-two-year lag was found as the time during which fertilizer leaks from Danish farmland realize as increased nutrient levels and primary production in the Danish and Schleswig-Holstein parts of the Wadden Sea |
| **Separate model 3**: Mussel flesh/shell ratio on the Wadden Sea | (S15) | Flesh/shell ratio of mussel biomass | The temperature along the Wadden Sea coastline (winter and spring) | 0 | Mussel flesh contents were measured in autumn. The effects of conditions during the preceding winter and spring on flesh/shell ratio hypothesized |
| " | " | " | Fertilizer | 1–2 | Maximally, the two-year lagged effect of application of fertilizers by Danish farmers on nutrient leaks, primary production, and flesh contents in mussels on the Wadden Sea was assumed |

## References

1. Møller, A. P. Long-term trends in wind speed, insect abundance and ecology of an insectivorous bird. *Ecosphere* **4**, 1–11 (2013).

2. Wood, S. N. *Generalized Additive Models: An Introduction with R*. (Chapman and Hall/CRC, 2006). doi:10.1201/9781315370279.

3. Zuur, A. F., Ieno, E. N., Walker, N. J., Saveliev, A. A. & Smith, G. M. *Mixed Effects Models and Extensions in Ecology with R.* (Springer, 2009). doi:10.1007/978-0-387-87458-6.

4. Dennis, B., Ponciano, J. M., Lele, S. R., Taper, M. L. & Staples, D. F. Estimating Density Dependence, Process Noise, and Observation Error. *Ecol. Monogr.* **76**, 323–341 (2006).

5. Kery, M. & Schaub, M. *Bayesian Population Analysis Using WinBUGS: A Hierarchical Perspective*. (Elsevier, 2012).

6. Gelman, A., Carlin, J. B., Stern, H. S. & Rubin, D. B. *Bayesian Data Analysis*. (Chapman & Hall/CRC, 2004).

7. Mutshinda, C. M., O’Hara, R. B. & Woiwod, I. P. A multispecies perspective on ecological impacts of climatic forcing. *J. Anim. Ecol.* **80**, 101–107 (2011).

8. Christensen, T. K. Havlit (Clangula hyemalis). *Deltag i vingeundersøgelsen 2018/19* 1–3 (2018).

9. Blicher-Mathiesen, G. *et al.* *Landovervågningsoplande 2018*. (NOVANA. DCE. Videnskabelig rapport nr. 352, 2019).

10. Duus, K. & Zinglersen, E. *Geografistatistik 2000*. (Geografforlaget, 2000).

11. Savchuk, O. P. Large-scale nutrient dynamics in the Baltic Sea, 1970–2016. *Front. Mar. Sci.* **5**, 95 (2018).

12. Saha, K. & Paul, S. Bias-corrected maximum likelihood estimator of the negative binomial dispersion parameter. *Biometrics* **61**, 179–185 (2005).

13. Kerman, J. Neutral noninformative and informative conjugate beta and gamma prior distributions. *Electron. J. Stat.* **5**, 1450–1470 (2011).

14. Plummer, M. JAGS Version 4.3.0 user manual. (2017).

15. Christensen, T. K. Havlit (Clangula hyemalis): Long-tailed Duck. *Vingeundersøgelsen* **2018**, 1–3 (2021).

16. Madsen, A. B. *et al.* *Vildtbestande og jagttider i Danmark*. (DCE. Videnskabelig rapport nr. 434, 2021).

17. Jensen, P. N. *et al.* *Vandmiljø og Natur 2017*. (NOVANA. DCE. Videnskabelig rapport nr. 309, 2019).

18. Conley, D. J. *et al.* Long-term changes and impacts of hypoxia in Danish coastal waters. *Ecol. Appl.* **17**, 165–184 (2007).

19. Crainiceanu, C. M., Ruppert, D. & Wand, M. P. Bayesian analysis for penalized spline regression using WinBUGS. *J. Stat. Softw.* **14**, (2005).

20. Nolet, B. a *et al.* Faltering lemming cycles reduce productivity and population size of a migratory Arctic goose species. *J. Anim. Ecol.* **82**, 804–13 (2013).

21. Kokorev, Y. I. & Kuksov, V. A. Population dynamics of lemmings, *Lemmus sibirica* and *Dicrostonyx torquatus*, and Arctic Fox *Alopex lagopus* on the Taimyr peninsula, Siberia, 1960–2001. *Ornis Svecica* **12**, 139–145 (2002).

22. Ehrich, D. *et al.* Documenting lemming population change in the Arctic: Can we detect trends? *Ambio* 786–800 (2019) doi:10.1007/s13280-019-01198-7.

23. Kausrud, K. L. *et al.* Linking climate change to lemming cycles. *Nature* **456**, 93–97 (2008).

24. Beukema, J. J., Dekker, R. & Philippart, C. J. M. Long-term variability in bivalve recruitment, mortality, and growth and their contribution to fluctuations in food stocks of shellfish-eating birds. *Mar. Ecol. Prog. Ser.* **414**, 117–130 (2010).

25. Laursen, K. & Møller, A. P. Long-Term changes in nutrients and mussel stocks are related to numbers of breeding eiders *Somateria mollissima* at a large Baltic colony. *PLoS One* **9**, e95851 (2014).

26. Carstensen, J., Andersen, J. H., Gustafsson, B. G. & Conley, D. J. Deoxygenation of the baltic sea during the last century. *Proc. Natl. Acad. Sci. U. S. A.* **111**, 5628–5633 (2014).

27. Pöysä, H. *et al.* Environmental variability and population dynamics: do European and North American ducks play by the same rules? *Ecol. Evol.* **6**, 7004–7014 (2016).

28. Ponciano, J. M., Taper, M. L. & Dennis, B. Ecological change points: The strength of density dependence and the loss of history. *Theor. Popul. Biol.* **121**, 45–59 (2018).

29. Gilks, W. R., Richardson, S. & Spiegelhalter, D. J. *Markov Chain Monte Carlo in Practice*. (Chapman & Hall, 1996).

30. R Core Team. R: A Language and Environment for Statistical Computing. (2020).

31. Sturtz, S., Ligges, U. & Gelman, A. R2WinBUGS : A Package for Running WinBUGS from R. *J. Stat. Softw.* **12**, 1–16 (2005).

32. Spiegelhalter, D. J., Best, N. G., Carlin, B. P. & van der Linde, A. Bayesian measures of model complexity and fit. *J R Stat. Soc B* **64**, 583–639 (2002).

# Supplementary tables

**Supplementary Table S1**. Summary of posterior distributions of parameters from a hierarchical model relating sub-models on lemmings (**a**) and long-tailed duck juvenile proportions (**b**) to the main model on long-tailed duck spring counts (**c**). The table reports mean estimates, standard deviations, 2.5- and 97.5-percentiles, median (50%), estimate for convergence , and Bayesian probability, *i.e.* degree of belief for a parameter being larger or, respectively, smaller than zero (*p*-value). The proportion of variance (Pr.) attributed to different factors also shown. is the posterior mean of the deviance and is a point estimate of the deviance (–2 * log(likelihood)) obtained by substituting in the posterior means of : thus, = –2 * log(*p*(*y*|)); is “the effective number of parameters” and is given by 32. values generally converged close to the minimum value of unity, indicating good convergence 6. “Code” refers to program code in Supplementary Methods.

## **Supplementary Table S1a**:Lemmings

| Variable or explanation | Parameter,  equation (1) | Code  (Supplementary  Methods) | Mean | SD | 2.5% | 50% | 97.5% |  | *p*-value |
| --- | --- | --- | --- | --- | --- | --- | --- | --- | --- |
| Initial population 1964, population 1 |  | L0 | 2.498 | - | - | - | - | - | - |
| Intercept |  | alpha_L_sc[1] | 3.674 | 1.84 | 0.18 | 3.65 | 7.39 | 1.115 | 0.982 |
| Scaling, population 2 |  | alpha_L_sc[2] | -6.283 | 2.37 | -10.90 | -6.27 | -1.53 | 1.009 | 0.994 |
| Scaling, population 3 |  | alpha_L_sc[3] | -7.246 | 1.82 | -10.78 | -7.29 | -3.64 | 1.008 | 1.000 |
| Summer precipitation, pop. 1 |  | bC_L_scale[1,1] | 1.200 | 0.43 | 0.37 | 1.18 | 2.03 | 1.201 | 0.997 |
| Autumn precipitation, pop. 1 |  | bC_L_scale[2,1] | -2.158 | 0.50 | -3.21 | -2.14 | -1.23 | 1.003 | 1.000 |
| Summer precipitation, scaling, pop. 2 |  | bC_L_scale[1,2] | -0.143 | 0.52 | -1.15 | -0.14 | 0.91 | 1.032 | 0.608 |
| Autumn precipitation, scaling, pop. 2 |  | bC_L_scale[2,2] | 2.219 | 0.65 | 1.02 | 2.22 | 3.52 | 1.002 | 1.000 |
| Summer precipitation, scaling, pop. 3 |  | bC_L_scale[1,3] | -0.082 | 0.53 | -1.20 | -0.06 | 0.91 | 1.022 | 0.547 |
| Autumn precipitation, scaling, pop. 3 |  | bC_L_scale[2,3] | 2.585 | 0.59 | 1.50 | 2.57 | 3.77 | 1.003 | 1.000 |
| 3-year periodicity, 1965–1994, vector |  | beta_3yr[1] | 1.134 | 0.15 | 0.84 | 1.14 | 1.41 | 1.006 | 1.000 |
| 3-year periodicity, 1995–2017, vector |  | beta_3yr[2] | -0.463 | 0.19 | -0.84 | -0.47 | -0.09 | 1.000 | 0.992 |
| Temporal trend effect |  | beta_yr | -0.002 | 0.01 | -0.03 | 0.00 | 0.02 | 1.007 | 0.547 |
| Density dependence |  | chi_L | -0.044 | 0.04 | -0.12 | -0.04 | 0.03 | 1.001 | 0.889 |
| Pr. summer prec. of climate var. |  | prop_L_beta1 | 0.163 | 0.08 | 0.05 | 0.15 | 0.35 | 1.123 | - |
| Pr. autumn prec. of climate var. |  | prop_L_beta2 | 0.837 | 0.08 | 0.65 | 0.85 | 0.95 | 1.115 | - |
| Pr. 3-year periodicity, 1965–1994 | Pr. | prop_beta_3yr[1] | 0.135 | 0.03 | 0.07 | 0.13 | 0.21 | 1.001 | - |
| Pr. 3-year periodicity, 1995–2017 | Pr. | prop_beta_3yr[2] | 0.019 | 0.01 | 0.00 | 0.02 | 0.05 | 1.001 | - |
| Pr. climate, populations combined |  | prop_clim_L | 0.293 | 0.07 | 0.16 | 0.29 | 0.44 | 1.011 | - |
| Pr. density dependence | Pr. | prop_dd_L | 0.249 | 0.04 | 0.17 | 0.25 | 0.32 | 1.016 | - |
| Pr. random error var., state population | Pr. | prop_eps_L_st | 0.297 | 0.05 | 0.22 | 0.29 | 0.44 | 1.004 | - |
| Pr. temporal effect | Pr. | prop_yr | 0.006 | 0.01 | 0.00 | 0.00 | 0.03 | 1.017 | - |
| Dispersion parameter, state pop. 1 |  | r_nb_L[1] | 8.646 | 5.29 | 1.60 | 7.40 | 19.13 | 1.001 | - |
| Dispersion parameter, state pop. 2 |  | r_nb_L[2] | 9.848 | 5.80 | 0.44 | 9.96 | 19.39 | 1.002 | - |
| Dispersion parameter, state pop. 3 |  | r_nb_L[3] | 9.897 | 5.87 | 0.58 | 9.90 | 19.52 | 1.000 | - |
| Observation error, high peak 1, pop. 1 |  | rnd_effect_peak[1] | 0.245 | 0.22 | 0.01 | 0.17 | 0.86 | 1.005 | - |
| Observation error, high peak 2, pop. 1 |  | rnd_effect_peak[2] | 0.611 | 0.42 | 0.02 | 0.56 | 1.59 | 1.009 | - |
| Observation error, high peak 3, pop. 1 |  | rnd_effect_peak[3] | 0.240 | 0.20 | 0.01 | 0.19 | 0.73 | 1.001 | - |
| Observation error, high peak 4, pop. 1 |  | rnd_effect_peak[4] | 0.610 | 0.41 | 0.02 | 0.57 | 1.55 | 1.015 | - |
| Observation error, high peak 5, pop. 1 |  | rnd_effect_peak[5] | 0.382 | 0.32 | 0.01 | 0.32 | 1.15 | 1.000 | - |
| Observation error SD, population 1 |  | sigma_L_o[1] | 0.084 | 0.01 | 0.06 | 0.08 | 0.12 | 1.001 | - |
| Observation error SD, population 2 |  | sigma_L_o[2] | 0.813 | 0.12 | 0.55 | 0.83 | 0.99 | 1.013 | - |
| Observation error SD, population 3 |  | sigma_L_o[3] | 0.005 | 0.00 | 0.00 | 0.01 | 0.01 | 1.018 | - |
| Random error SD, state population 1 |  | sigma_L_rnd[1] | 0.460 | 0.04 | 0.36 | 0.47 | 0.50 | 1.003 | - |
| Random error SD, state population 2 |  | sigma_L_rnd[2] | 0.273 | 0.14 | 0.02 | 0.29 | 0.49 | 1.003 | - |
| Random error SD, state population 3 |  | sigma_L_rnd[3] | 0.279 | 0.14 | 0.02 | 0.30 | 0.49 | 1.010 | - |
| SD negative binomial process error, pop. 1 |  | sigma_nbL[1] | 0.734 | 0.11 | 0.55 | 0.72 | 0.99 | 1.012 | - |
| SD negative binomial process error, pop. 2 |  | sigma_nbL[2] | 0.593 | 0.21 | 0.39 | 0.53 | 1.26 | 1.002 | - |
| SD negative binomial process error, pop. 3 |  | sigma_nbL[3] | 0.633 | 0.17 | 0.45 | 0.59 | 1.13 | 1.001 | - |
| Weight, July precipitation |  | wL[1] | 0.566 | 0.16 | 0.26 | 0.57 | 0.90 | 1.005 | - |
| Weight, October precipitation |  | wL[2] | 0.480 | 0.11 | 0.26 | 0.48 | 0.71 | 1.010 | - |

## **Supplementary Table S1b**:Juvenile proportions

| Variable or explanation | Parameter,  equation (2) | Code  (Supplementary  Methods) | Mean | SD | 2.5% | 50% | 97.5% |  | *p*-value |
| --- | --- | --- | --- | --- | --- | --- | --- | --- | --- |
| Intercept |  | alpha_juv | 6.337 | 1.65 | 3.15 | 6.38 | 9.69 | 1.007 | 1.000 |
| May + June precipitation, NW Siberia |  | bCj[1] | -2.192 | 0.52 | -3.15 | -2.20 | -1.14 | 1.010 | 1.000 |
| May + June temperature, NW Siberia |  | bCj[2] | 0.117 | 0.12 | -0.14 | 0.13 | 0.32 | 1.000 | 0.858 |
| Lemming |  | beta_L | -0.213 | 0.13 | -0.46 | -0.21 | 0.04 | 1.001 | 0.953 |
| NAOI, three years before |  | beta_nao3 | -0.279 | 0.11 | -0.50 | -0.28 | -0.07 | 1.001 | 0.994 |
| Pr. Lemming | Pr. | prop.beta_L | 0.091 | 0.08 | 0.00 | 0.07 | 0.28 | 1.001 | - |
| Pr. NAOI | Pr. | prop.beta_nao3 | 0.127 | 0.08 | 0.01 | 0.11 | 0.31 | 1.001 | - |
| Pr. residual error var. | Pr. | prop.resid_juv | 0.252 | 0.19 | 0.00 | 0.24 | 0.63 | 1.019 | - |
| Pr. random error var. | Pr. | prop.rnd_juv | 0.267 | 0.19 | 0.00 | 0.27 | 0.61 | 1.000 | - |
| Pr. May + June precipitation | Pr. | prop_bCj[1] | 0.209 | 0.08 | 0.05 | 0.21 | 0.38 | 1.005 | - |
| Pr. May + June temperature | Pr. | prop_bCj[2] | 0.054 | 0.05 | 0.00 | 0.04 | 0.19 | 1.001 | - |
| SD random error |  | sigma_juv[1] | 0.474 | 0.23 | 0.03 | 0.52 | 0.85 | 1.001 | - |
| SD residual error |  | sigma_juv[2] | 0.452 | 0.23 | 0.03 | 0.49 | 0.84 | 1.019 | - |
| Weight, June precipitation |  | wj[1] | 0.227 | 0.15 | 0.01 | 0.21 | 0.54 | 1.000 | - |
| Weight June temperature |  | wj[2] | 0.659 | 0.26 | 0.07 | 0.71 | 0.99 | 1.002 | - |

## **Supplementary Table S1c**:Long-tailed duck spring counts

| Variable or explanation | Parameter,  equation (4) | Code  (Supplementary  Methods) | Mean | SD | 2.5% | 50% | 97.5% |  | *p*-value |
| --- | --- | --- | --- | --- | --- | --- | --- | --- | --- |
| Intercept |  | alpha_LtD | 10.070 | 1.12 | 7.61 | 10.26 | 11.61 | 1.003 | 1.000 |
| DIN + DIP effect |  | beta_din | 0.268 | 0.09 | 0.10 | 0.27 | 0.45 | 1.001 | 1.000 |
| Juvenile proportion |  | beta_juv | 0.246 | 0.17 | 0.01 | 0.21 | 0.66 | 1.004 | 1.000 |
| E-W aspect wind, observation error |  | beta_o_east | 0.203 | 0.06 | 0.08 | 0.20 | 0.32 | 1.002 | 0.999 |
| Density dependence |  | dd_LtD | 0.134 | 0.09 | 0.01 | 0.12 | 0.34 | 1.007 | - |
| Pr. DIN + DIP effect | Pr. | prop.beta_din | 0.239 | 0.12 | 0.04 | 0.23 | 0.48 | 1.000 | - |
| Pr. Juvenile proportion | Pr. | prop.beta_juv | 0.054 | 0.06 | 0.00 | 0.03 | 0.21 | 1.005 | - |
| Pr. Density dependence | Pr. | prop.dd_LtD | 0.216 | 0.07 | 0.12 | 0.20 | 0.39 | 1.003 | - |
| Pr. random environmental disturbance | Pr. , | prop.sigma_pLTD | 0.492 | 0.15 | 0.20 | 0.50 | 0.74 | 1.000 | - |
| Pr. E-W aspect wind (* | Pr. | prop_beta_east | 0.508 | 0.27 | 0.08 | 0.46 | 1.00 | 1.000 | - |
| Pr. observation error, unknown sources (* | Pr. | prop_sigma_oLTD | 0.492 | 0.27 | 0.00 | 0.54 | 0.92 | 1.009 | - |
| Dispersion parameter |  | r_nb_ltd | 28.817 | 12.14 | 8.71 | 28.63 | 48.70 | 1.001 | - |
| SD negative binomial state process error |  | sigma_nb_ltd | 0.204 | 0.06 | 0.13 | 0.19 | 0.36 | 1.001 | - |
| SD observation error, unknown sources |  | sigma_oLTD | 0.214 | 0.11 | 0.01 | 0.23 | 0.40 | 1.004 | - |
| SD random environmental disturbance | SD | sigma_pLTD | 0.192 | 0.11 | 0.01 | 0.19 | 0.40 | 1.011 | - |
| Weight, DIP |  | wD | 0.489 | 0.16 | 0.14 | 0.49 | 0.83 | 1.000 | - |

*) Proportions of total observation error

Deviance statistics. Supplementary Tables 1a, b and c combined: = -76.61; = -5689.96; = 5613.34

**Supplementary Table S2**. Summary of posterior distributions of parameters for DIN and DIP amounts in the Danish Straits (DS) and Baltic Proper (BP). The table reports mean estimates, standard deviations, 2.5- and 97.5-percentiles, median (50%), estimate for convergence , and Bayesian probability or degree of belief for a parameter being larger or, respectively, smaller than zero (*p*-value). The proportion of variance (Pr.) attributed to different factors also shown. is the posterior mean of the deviance and is a point estimate of the deviance (–2 * log(likelihood)) obtained by substituting in the posterior means of : thus, = –2 * log(*p*(*y*|)); is “the effective number of parameters” and is given by 32. values generally converged close to the minimum value of unity, indicating good convergence 6. “Code” refers to program code in Supplementary Methods.

## **Supplementary Table S2**:DIN and DIP

| Variable or explanation | Parameter,  equation (3) | Code  (Supplementary  Methods) | Mean | SD | 2.5% | 50% | 97.5% |  | *p*-value |
| --- | --- | --- | --- | --- | --- | --- | --- | --- | --- |
| Intercept, DIN |  | a0[1] | -21.683 | 21.62 | -65.85 | -21.71 | 20.94 | 1.003 | 0.851 |
| Intercept, DIP |  | a0[2] | -14.016 | 14.32 | -37.13 | -15.12 | 19.39 | 1.004 | 0.859 |
| Structural zero |  | alpha[1,1] | 0.000 | 0.00 | 0.00 | 0.00 | 0.00 | 1.000 | 0.000 |
| BP, DIN; intercept, observation process |  | alpha[2,1] | 3.435 | 0.04 | 3.36 | 3.43 | 3.52 | 1.005 | 1.000 |
| Structural zero |  | alpha[1,2] | 0.000 | 0.00 | 0.00 | 0.00 | 0.00 | 1.000 | 0.000 |
| BP, DIP; intercept, observation process |  | alpha[2,2] | 4.296 | 0.05 | 4.21 | 4.29 | 4.38 | 1.003 | 1.000 |
| Fertilizer effect, DIN |  | b_X[1] | 4.212 | 3.79 | -3.13 | 4.22 | 12.03 | 1.004 | 0.880 |
| Fertilizer effect, DIP |  | b_X[2] | 2.455 | 2.49 | -3.30 | 2.68 | 6.46 | 1.004 | 0.863 |
| Fertilizer-smoother  (Knot 1) DIN |  | b_Z[1,1] | 2.113 | 2.33 | -2.42 | 1.99 | 6.69 | 1.000 | 0.831 |
| (Knot 1) DIP |  | b_Z[2,1] | 2.351 | 1.69 | -2.02 | 2.53 | 4.84 | 1.010 | 0.926 |
| (Knot 2) DIN |  | b_Z[1,2] | 4.703 | 4.05 | -3.43 | 4.57 | 13.10 | 1.002 | 0.888 |
| (Knot 2) DIP |  | b_Z[2,2] | 3.199 | 2.47 | -2.67 | 3.42 | 7.18 | 1.002 | 0.904 |
| (Knot 3) DIN |  | b_Z[1,3] | 0.843 | 6.82 | -12.35 | 0.90 | 13.75 | 1.007 | 0.559 |
| (Knot 3) DIP |  | b_Z[2,3] | 4.896 | 4.52 | -5.22 | 5.04 | 12.79 | 1.011 | 0.886 |
| (Knot 4) DIN |  | b_Z[1,4] | -1.172 | 3.84 | -8.69 | -1.20 | 6.71 | 1.006 | 0.628 |
| (Knot 4) DIP |  | b_Z[2,4] | -0.423 | 2.45 | -4.86 | -0.63 | 4.68 | 1.002 | 0.596 |
| (Knot 5) DIN |  | b_Z[1,5] | -1.409 | 2.19 | -5.69 | -1.50 | 2.94 | 1.007 | 0.754 |
| (Knot 5) DIP |  | b_Z[2,5] | -0.978 | 1.39 | -3.38 | -1.13 | 2.11 | 1.004 | 0.783 |
| Autoregression, DIN |  | chi_din[1] | 0.173 | 0.15 | 0.01 | 0.14 | 0.62 | 1.048 | 1.000 |
| Autoregression, DIP |  | chi_din[2] | 0.871 | 0.13 | 0.59 | 0.89 | 1.09 | 1.018 | 1.000 |
| Lag (years), fertilizer and |  | lag | 0.948 | 0.23 | 0.00 | 1.00 | 1.00 | 1.089 | 0.945 |
| Binomial-distribution parameter for |  | lag_theta | 0.623 | 0.20 | 0.21 | 0.64 | 0.94 | 1.008 | - |
| Pr. autoregression, DIN | Pr. | prop.ar_din[1] | 0.042 | 0.07 | 0.00 | 0.01 | 0.29 | 1.036 | - |
| Pr. autoregression, DIP | Pr. | prop.ar_din[2] | 0.169 | 1.58 | -0.19 | 0.04 | 0.87 | 1.100 | - |
| Pr. fertilizer effect, DIN | Pr. | prop.m_X[1] | 0.524 | 0.18 | 0.02 | 0.57 | 0.75 | 1.002 | - |
| Pr. fertilizer effect, DIP | Pr. | prop.m_X[2] | 0.379 | 0.79 | 0.00 | 0.45 | 0.57 | 1.133 | - |
| Pr. fertilizer smoother, DIN |  | prop.m_X_spl[1] | 0.394 | 0.14 | 0.10 | 0.40 | 0.77 | 1.018 | - |
| Pr. fertilizer smoother, DIP |  | prop.m_X_spl[2] | 0.421 | 0.68 | 0.04 | 0.49 | 0.60 | 1.037 | - |
| Pr. state-process error variance, DIN | Pr. | prop.sigma_st_din[1] | 0.039 | 0.07 | 0.00 | 0.01 | 0.27 | 1.041 | - |
| Pr. state-process error variance, DIP | Pr. | prop.sigma_st_din[2] | 0.030 | 0.15 | 0.00 | 0.01 | 0.23 | 1.135 | - |
| Pr. observation error variance, DS, DIN | Pr. | prop_obs_error[1,1] | 0.298 | 0.07 | 0.19 | 0.29 | 0.44 | 1.002 | - |
| Pr. observation error variance, BP, DIN | Pr. | prop_obs_error[2,1] | 0.085 | 0.04 | 0.01 | 0.08 | 0.17 | 1.004 | - |
| Pr. observation error variance, DS, DIP | Pr. | prop_obs_error[1,2] | 0.607 | 0.07 | 0.46 | 0.61 | 0.74 | 1.011 | - |
| Pr. observation error variance, BP, DIP | Pr. | prop_obs_error[2,2] | 0.010 | 0.01 | 0.00 | 0.01 | 0.03 | 1.014 | - |
| SD observation error, DS, DIN |  | sigma_din[1,1] | 0.230 | 0.03 | 0.18 | 0.23 | 0.29 | 1.000 | - |
| SD observation error, BP, DIN |  | sigma_din[2,1] | 0.120 | 0.03 | 0.05 | 0.12 | 0.18 | 1.004 | - |
| SD observation error, DS, DIP |  | sigma_din[1,2] | 0.331 | 0.04 | 0.27 | 0.33 | 0.42 | 1.011 | - |
| SD observation error, BP, DIP |  | sigma_din[2,2] | 0.037 | 0.02 | 0.00 | 0.04 | 0.07 | 1.016 | - |
| SD state-process error, DIN | SD | sigma_st_din[1] | 0.083 | 0.04 | 0.00 | 0.09 | 0.16 | 1.134 | - |
| SD state-process error, DIP | SD | sigma_st_din[2] | 0.059 | 0.02 | 0.01 | 0.06 | 0.10 | 1.115 | - |

Deviance statistics (DIN and DIP): = -252.59; = -3985.47; = 3732.88

**Supplementary Table S3**. Summary of posterior distributions of parameters from hierarchical models for (**a**) mussel biomass in the Danish (DK) and Schleswig-Holstein (S-H) parts of the Wadden Sea and (**b**) flesh/shell ratio (DK). The table reports mean estimates, standard deviations, 2.5- and 97.5-percentiles, median (50%), estimate for convergence , and Bayesian probability or degree of belief for a parameter being larger or, respectively, smaller than zero (*p*-value). The proportion of variance (Pr.) attributed to different factors also shown. is the posterior mean of the deviance and is a point estimate of the deviance (–2 * log(likelihood)) obtained by substituting in the posterior means of : thus, = –2 * log(*p*(*y*|)); is “the effective number of parameters” and is given by 32. values generally converged close to the minimum value of unity, indicating good convergence 6. “Code” refers to program code in Supplementary Methods.

## **Supplementary Table S3a**:Mussel biomass

| Variable or explanation | Parameter,  equation (5) | Code  (Supplementary  Methods) | Mean | SD | 2.5% | 50% | 97.5% |  | *p*-value |
| --- | --- | --- | --- | --- | --- | --- | --- | --- | --- |
| Initial population 1 (1986) |  | a0 | 10.536 | 0.50 | 9.53 | 10.56 | 11.49 | 1.001 | 1.000 |
| Intercept |  | b0 | 75.224 | 195.02 | -285.12 | 75.13 | 426.91 | 1.000 | 0.640 |
| Winter temperature |  | b1_Tw | -0.105 | 0.07 | -0.24 | -0.11 | 0.04 | 1.000 | 0.918 |
| Fertilizer smoother , knot 1 |  | b_fZ[1] | -7.405 | 23.15 | -50.77 | -7.30 | 35.58 | 1.000 | 0.617 |
| , knot 2 |  | b_fZ[2] | -14.178 | 36.70 | -82.02 | -13.47 | 54.40 | 1.000 | 0.642 |
| , knot 3 |  | b_fZ[3] | -2.846 | 48.71 | -106.53 | -2.25 | 85.28 | 1.002 | 0.521 |
| , knot 4 |  | b_fZ[4] | 10.200 | 26.23 | -43.40 | 10.23 | 58.40 | 1.000 | 0.652 |
| , knot 5 |  | b_fZ[5] | 10.614 | 20.97 | -30.69 | 11.35 | 47.59 | 1.000 | 0.688 |
| Fertilizer |  | b_fer | -12.409 | 33.82 | -73.25 | -12.09 | 50.29 | 1.000 | 0.636 |
| Density dependence |  | chi | 0.650 | 0.19 | 0.21 | 0.67 | 0.97 | 1.078 | 1.000 |
| Lag, and |  | lag_m_fer | 1.757 | 0.83 | 0.00 | 2.00 | 3.00 | 1.006 | 0.937 |
| Pr. winter temperature (* | Pr. | prop1_T_winter | 0.016 | 0.04 | 0.00 | 0.00 | 0.12 | 1.003 | - |
| Pr. fertilizer (* | Pr. | prop2_fer | 0.849 | 0.25 | 0.07 | 0.97 | 1.00 | 1.056 | - |
| Pr. density dependence (* | Pr. | prop_m_dd | 0.097 | 0.16 | 0.00 | 0.02 | 0.61 | 1.000 | - |
| Pr. demographic variance (* | Pr. | prop_sigma_dem | 0.018 | 0.04 | 0.00 | 0.00 | 0.15 | 1.004 | - |
| Pr. environmental variance (* | Pr. | prop_sigma_st | 0.020 | 0.05 | 0.00 | 0.00 | 0.17 | 1.002 | - |
| Pr. fertilizer smoother of total var. (** | Pr. | prop_spl_of_tot | 0.462 | 0.09 | 0.17 | 0.50 | 0.55 | 1.028 | - |
| SD environmental variance |  | sigma.st | 0.217 | 0.13 | 0.01 | 0.21 | 0.52 | 1.005 | - |
| SD demographic variance, DK |  | sigma_dem[1] | 0.434 | 0.16 | 0.09 | 0.43 | 0.76 | 1.021 | - |
| SD demographic variance, S-H |  | sigma_dem[2] | 0.118 | 0.09 | 0.00 | 0.10 | 0.36 | 1.007 | - |
| SD observation error, DK |  | sigma_obs[1] | 0.282 | 0.17 | 0.02 | 0.28 | 0.63 | 1.010 | - |
| SD observation error, S-H |  | sigma_obs[2] | 0.123 | 0.09 | 0.01 | 0.10 | 0.35 | 1.004 | - |
| Weight, winter temperature, 2-year lag |  | weight | 0.604 | 0.26 | 0.07 | 0.63 | 0.98 | 1.017 | - |

*) Proportion of variance excluding fertilizer smoother effect. **) Proportional variance of of variance explained by all parameters

Deviance statistics (mussel biomass): = -46.73; = -1723.48; = 1676.75

## **Supplementary Table S3b**: Mussel flesh/shell ratio

| Variable or explanation | Parameter,  equation (6) | Code  (Supplementary  Methods) | Mean | SD | 2.5% | 50% | 97.5% |  | *p*-value |
| --- | --- | --- | --- | --- | --- | --- | --- | --- | --- |
| Intercept |  | alpha_f | -11.575 | 2.47 | -17.14 | -11.47 | -6.86 | 1.001 | 1.000 |
| Temperature, winter |  | f_beta[1] | 0.055 | 0.03 | 0.00 | 0.06 | 0.11 | 1.001 | 0.982 |
| Temperature, spring |  | f_beta[2] | 0.005 | 0.04 | -0.07 | 0.00 | 0.08 | 1.003 | 0.553 |
| Fertilizer |  | f_beta[3] | 1.633 | 0.44 | 0.78 | 1.61 | 2.61 | 1.001 | 0.999 |
| Lag, fertilizer |  | lag_fer | 1.482 | 0.66 | 0.00 | 2.00 | 2.00 | 1.001 | 0.909 |
| Pr. temperature, winter | Pr. | prop_f_beta[1] | 0.189 | 0.13 | 0.00 | 0.17 | 0.46 | 1.012 | - |
| Pr. temperature, spring | Pr. | prop_f_beta[2] | 0.030 | 0.04 | 0.00 | 0.01 | 0.15 | 1.002 | - |
| Pr. fertilizer | Pr. | prop_f_beta[3] | 0.423 | 0.16 | 0.10 | 0.44 | 0.69 | 1.010 | - |
| Pr. random disturbances | Pr. | prop_f_rnd | 0.177 | 0.15 | 0.00 | 0.15 | 0.53 | 1.002 | - |
| Pr. residual error | Pr. | prop_f_sigma | 0.181 | 0.15 | 0.00 | 0.16 | 0.55 | 1.007 | - |
| SD residual error |  | sigma_f | 0.085 | 0.05 | 0.00 | 0.09 | 0.19 | 1.006 | - |
| SD random disturbances | SD | sigma_f_rnd | 0.084 | 0.05 | 0.00 | 0.09 | 0.18 | 1.001 | - |

Deviance statistics (flesh/shell ratio): = -43.85; = -514.57; = 470.72

# Supplementary Methods: Program codes

Running the Bayesian models with the below program codes requires installing R ([https://cran.r-project.org](https://cran.r-project.org/)) and JAGS (<https://sourceforge.net/projects/mcmc-jags>). R workspace ‘BUGS_data.RData’ contains list-type datasets (Supplementary Data) applicable with the coding. The ‘R2jags’ package needs to be installed as: install.packages("R2jags"). Set working directory according to: setwd("C:/DEFAULT_DIR"), and load library(R2jags).

## BUGS code for the hierarchical model on the long-tailed duck spring counts, juvenile proportions, and lemmings

# Model on the long-tailed duck spring counts, juvenile proportions, and lemmings

sink("mod5.r")

cat("

model{

# Parameters and priors

# Long-tailed duck spring counts

prec_pLTD<-1/sigma2_pLTD

sigma2_pLTD<-pow(sigma_pLTD,2)

sigma_pLTD ~ dunif(0, 5)

prec_oLTD<-1/sigma2_oLTD

sigma2_oLTD<-pow(sigma_oLTD,2)

sigma_oLTD~dunif(0, 10)

alpha_LtD ~ dnorm(mean_alpha_LtD, 0.2)

beta_juv ~ dbeta(1,1)

dd_LtD ~ dnorm(0, 1)T(0,1)

beta_o_east ~ dnorm(0, 1.0E-6)

for(j in 1:n_beta_din){

beta_din[j] ~ dnorm(0, 1.0E-6)

}

for(j in 1:n_wD){

wD[j] ~ dbeta(1,1)

}

r_nb_ltd ~ dunif(0,50)

for(nutrient in 1:2){

sigma_pool[nutrient] ~ dunif(0,2)

isigma2_pool[nutrient] <- pow(sigma_pool[nutrient],-2)

}

for(i in 1:3){ # 1967-1969

for(nutrient in 1:2){

pool[i,nutrient] ~ dnorm(pool_priors[i,nutrient], isigma2_pool[nutrient])

}

}

# Long-tailed duck juvenile proportions

for(j in 1:2){

prec_juv[j]<-1/sigma2_juv[j]

sigma2_juv[j]<-pow(sigma_juv[j],2)

sigma_juv[j]~dunif(0,5)

}

alpha_juv ~ dnorm(mean_alpha_juv, 0.1)

for(j in 1:n_beta_L){

beta_L[j] ~ dnorm(mean_beta_L, 0.1)

}

for(j in 1:n_bCj){

bCj[j] ~ dnorm(mean_bCj[j], 0.1)

}

for(j in 1:n_bCj){

wj[j]~dbeta(1,1)

}

beta_nao3 ~ dnorm(mean_beta_nao3, 0.1)

# Lemmings

alpha_L_sc[1]~dnorm(mean_alpha_L[1], 0.1)

alpha_L_sc[2]~dnorm(mean_alpha_L[2], 0.1)

alpha_L_sc[3]~dnorm(mean_alpha_L[3], 0.1)

for(j in 1:max(chi_pop)){

chi_L[j]~dnorm(mean_chi_L,10)I(-1,1)

}

for(j in 1:n_wL_para){

wL[j]~dbeta(1,1)

}

## climate parameters j for population k=1

for(j in 1:n_clim_variable){

bC_L_scale[j,1]~dnorm(mean_betaC[j,1], 1.0E-6)

}

## j's for populations k=2 and k=3 (scaling)

bC_L_scale[1,2]~dnorm(mean_betaC[1,2], 0.1)

bC_L_scale[2,2]~dnorm(mean_betaC[2,2], 0.1)

bC_L_scale[1,3]~dnorm(mean_betaC[1,3], 0.1)

bC_L_scale[2,3]~dnorm(mean_betaC[2,3], 0.1)

prec_L_o[1] <- 1/sigma2_L_o[1]

sigma2_L_o[1]<-pow(sigma_L_o[1],2)

sigma_L_o[1]~dunif(0, 1)

prec_L_o[2] <- 1/sigma2_L_o[2]

sigma2_L_o[2]<-pow(sigma_L_o[2],2)

sigma_L_o[2]~dunif(0, 1)

prec_L_o[3] <- 1/sigma2_L_o[3]

sigma2_L_o[3]<-pow(sigma_L_o[3],2)

sigma_L_o[3]~dunif(0, 0.01)

prec_L_rnd[1] <- 1/pow(sigma_L_rnd[1],2)

sigma_L_rnd[1]~dunif(0, 0.5)

prec_L_rnd[2] <- 1/pow(sigma_L_rnd[2],2)

sigma_L_rnd[2]~dunif(0, 0.5)

prec_L_rnd[3] <- 1/pow(sigma_L_rnd[3],2)

sigma_L_rnd[3]~dunif(0, 0.5)

for(i in 1:n_peaks){

rnd_effect_peak[i] ~ dnorm(0,1)I(0,)

}

for(i in 1:length(indicator_peak_yrs)){

alpha_peak[indicator_peak_yrs[i]] <- rnd_effect_peak[i]

}

for(i in 1:length(indicator_not_peak_yrs)){

alpha_peak[indicator_not_peak_yrs[i]] <- 0

}

for(j in 1:max(yr_pop)){

beta_yr[j] ~ dnorm(mean_beta_yr, 0.1) #, prec_beta_yr

}

L0 <- L_ini # 1964

r_nb_L[1] ~ dunif(0,20)

r_nb_L[2] ~ dunif(0,20)

r_nb_L[3] ~ dunif(0,20)

# Likelihoods

# State process, long-tailed duck

## Climate variables

for(i in 1:N){ # [1,] refers to 1964 in Tmx_s[,] etc. arrays

Tltd5[i] <- (Tmn_s[i+3,5]+Tmx_s[i+3,5])/2

Tltd6[i] <- (Tmn_s[i+3,6]+Tmx_s[i+3,6])/2

Pltd5[i] <- log(Pre_s[i+3,5])

Pltd6[i] <- log(Pre_s[i+3,6])

}

## DIN & DIP variable

for(i in 1:(N-1)){ # pool[year_i,area_j,nutrient_k] # DS,BP

## pool[1,] refers to 1967 # could start from 1968!

d12[i] <- (1-wD[1])*pool[i+1,1] + wD[1]* -1*pool[i+1,2]

}

mu_ltd[1] <- 11.65635 + rnd_ltd[1] # observed (log) value of 1968

eps[1] <- mu_ltd[1]-log(state_ltd[1])

log(lamda_ltd[1]) <- mu_ltd[1]

p_ltd[1] <- r_nb_ltd/(r_nb_ltd + lamda_ltd[1])

state_ltd[1] ~ dnegbin(p_ltd[1],r_nb_ltd)

log_state_ltd[1] <- log(state_ltd[1])

st_1000fems[1] <- state_ltd[1]/1000

rnd_ltd[1] ~ dnorm(0, prec_pLTD)

for(i in 1:(N-1)){ # 1968:(2014-1)

mu_ltd[i+1] <- alpha_LtD

+ dd_LtD*log_state_ltd[i]

+ beta_din[1]*d12[i]

+ beta_juv*log(r[i+1])

+ rnd_ltd[i+1]

log(lamda_ltd[i+1]) <- mu_ltd[i+1]

p_ltd[i+1] <- r_nb_ltd/(r_nb_ltd + lamda_ltd[i+1])

state_ltd[i+1] ~ dnegbin(p_ltd[i+1],r_nb_ltd)

eps[i+1] <- mu_ltd[i+1]-log(state_ltd[i+1])

log_state_ltd[i+1] <- log(state_ltd[i+1])

st_1000fems[i+1] <- state_ltd[i+1]/1000

rnd_ltd[i+1] ~ dnorm(0, prec_pLTD)

}

# Observation process, long-tailed duck

for(i in 1:N){

n_mu_ltd[i] <- log_state_ltd[i] + beta_o_east*east[i]

n[i] ~ dnorm(n_mu_ltd[i],prec_oLTD)

epso[i] <- n[i]-log_state_ltd[i]

}

# Variance partition, long-tailed duck

sigma_nb_ltd <- sd(eps[])

var.dd_LtD <- s_var*(1-pow(dd_LtD,2)) # cf. stationary var.: Dennis et al. 2006

var.beta_juv <- pow(beta_juv,2)*pow(sd(log(r[2:N])),2)

var.beta_din <- pow(beta_din[1],2)*pow(sd(d12[]),2)

tot_var_pLtD <- var.dd_LtD + pow(sigma_pLTD,2) + var.beta_juv

+ var.beta_din + pow(sigma_nb_ltd,2)

prop.dd_LtD <- var.dd_LtD/tot_var_pLtD

prop.beta_juv <- var.beta_juv/tot_var_pLtD

prop.beta_din <- var.beta_din/tot_var_pLtD

prop.sigma_pLTD <- (pow(sigma_pLTD,2) + pow(sigma_nb_ltd,2))/tot_var_pLtD

prop_beta_east <- pow(beta_o_east,2)/(pow(beta_o_east,2) + sigma2_oLTD)

prop_sigma_oLTD <- sigma2_oLTD/(pow(beta_o_east,2) + sigma2_oLTD)

prop_obs_error_LtD <- (pow(beta_o_east,2) + sigma2_oLTD)/(pow(beta_o_east,2) + sigma2_oLTD + tot_var_pLtD)

# Process, juvenile proportions

## Climate variables

for(i in 1:N_juv){ # [1,] refers to 1964 in Tmx_s[,] etc. arrays

Tj5[i] <- (Tmn_s[i+3,5]+Tmx_s[i+3,5])/2

Tj6[i] <- (Tmn_s[i+3,6]+Tmx_s[i+3,6])/2

Pj5[i] <- log(Pre_s[i+3,5])

Pj6[i] <- log(Pre_s[i+3,6])

Climj[i,1] <- (1-wj[1])*Pj5[i] + wj[1]*Pj6[i]

Climj[i,2] <- (1-wj[2])*Tj5[i] + wj[2]*Tj6[i]

}

for(i in 1:N_juv){ # 1967:2017

logitp_mu[i] <- alpha_juv

+ beta_nao3*nao[i] # nao[1] refers to 1964

+ beta_L[1]*log_L_st[i+1,1] # [1] refers to 1965

+ bCj[1]*Climj[i,1]

+ bCj[2]*Climj[i,2]

+ rnd_juv[i]

rnd_juv[i] ~ dnorm(0,prec_juv[1])

logitp_obs[i] ~ dnorm(logitp_mu[i],prec_juv[2])

p[i] <- exp(logitp_mu[i])/(1 + exp(logitp_mu[i]))

mu_ratio[i] <- p[i]/(1-p[i]) # juv fem / ad fem ratio

r[i] <- mu_ratio[i]+1 # growth rate (multiplicative)

eps_juv[i] <- logitp_mu[i] - logitp_obs[i]

}

# Variance partition, juveniles

var.beta_nao3 <- pow(beta_nao3,2)*pow(sd(nao[1:N_juv]),2)

var.beta_L <- pow(beta_L[1],2)*pow(sd(log_L_st[1:N_juv+1,1]),2)

for(j in 1:n_bCj){

var.bCj[j] <- pow(bCj[j],2)*pow(sd(Climj[1:N_juv,j]),2)

}

tot_var_juv <- var.beta_nao3 + var.beta_L + sum(var.bCj[]) + sum(sigma2_juv[])

prop.beta_nao3 <- var.beta_nao3/tot_var_juv

prop.beta_L <- var.beta_L/tot_var_juv

prop.rnd_juv <- sigma2_juv[1]/tot_var_juv

prop.resid_juv <- sigma2_juv[2]/tot_var_juv

for(j in 1:n_bCj){

prop_bCj[j] <- var.bCj[j]/tot_var_juv

}

# State process, lemmings

for(period in 1:2){# for periods

beta_3yr[period] ~ dnorm(0, 0.01)

}

for(i in 1:N_L){

for(j in 1:10){ # lemming peaks 1:10

D1j[i,j] <- beta_3yr[1]*Z_1[i,j] + beta_3yr[1]*pow(Z_1[i,j],2) # likelihood for the period 1965:1994

}

for(j in 11:n_b){ # peaks 11:n_b

D2j[i,j-10] <- beta_3yr[2]*Z_1[i,j] + beta_3yr[2]*pow(Z_1[i,j],2) # likelihood for the period 1995:2017

}

D1[i] <- sum(D1j[i,])

D2[i] <- sum(D2j[i,])

}

## Climate variables

for(i in 1:N_L){ # [1,,] refers to 1964 in Pre[,,] etc. arrays

for(j in 1:n_pop){

P6[i,j] <- log(Pre[i+1,6,j])

P7[i,j] <- log(Pre[i+1,7,j])

P9[i,j] <- log(Pre[i,9,j])

P10[i,j] <- log(Pre[i,10,j])

Clim[i,1,j] <- (1-wL[1])*P6[i,j] + wL[1]*P7[i,j]

Clim[i,2,j] <- (1-wL[2])*P9[i,j] + wL[2]*P10[i,j]

}

Climm[i,1] <- mean(Clim[i,1,])

Climm[i,2] <- mean(Clim[i,2,])

}

for(i in 1:(N_L-1)){

## 1st population, 1965 + 1

tau0[i,1] ~ dbeta(1,100)

tau[i,1] <- ifelse(indicator0[i] > 0, log(tau0[i,1]), log_L_st[i,1])

L_mu[i+1,1] <- alpha_L_sc[1] + D1[i+1] + D2[i+1]

+ chi_L[chi_pop[1]]*tau[i,1]

+ beta_yr[yr_pop[1]]*yr[i+1]

+ bC_L_scale[1,1]*Clim[i+1,1,1]

+ bC_L_scale[2,1]*Clim[i+1,2,1]

+ rnd_L[i+1,1]

log(lamda[i+1,1]) <- L_mu[i+1,1]

p_L[i+1,1] <- r_nb_L[1]/(r_nb_L[1] + lamda[i+1,1])

L_st[i+1,1] ~ dnegbin(p_L[i+1,1],r_nb_L[1])

log_L_st[i+1,1] <- log(L_st[i+1,1]+1)

rnd_L[i+1,1] ~ dnorm(0,prec_L_rnd[1]) # random effect for popul. 1

eps_LPearson[i+1,1] <- (L_mu[i+1,1]-log_L_st[i+1,1])/sqrt(var_L_mu[1]) # Pearson residual

eps_L[i+1,1] <- L_mu[i+1,1]-log_L_st[i+1,1]

# Observation process, lemmings

L_st_mu[i+1,1] <- log_L_st[i+1,1] - alpha_peak[i+1]

lemmings[i+1,1] ~ dnorm(L_st_mu[i+1,1], prec_L_o[prec_pop[1]])

eps_L_o[i+1,1] <- lemmings[i+1,1]-L_st_mu[i+1,1]

## 2nd and 3rd populations

for(k in 2:3){

L_mu[i+1,k] <- alpha_L_sc[k] + L_mu[i+1,1]

+ bC_L_scale[1,k]*Clim[i+1,1,k] # scaling

+ bC_L_scale[2,k]*Clim[i+1,2,k]

+ rnd_L[i+1,k]

log(lamda[i+1,k]) <- L_mu[i+1,k]

p_L[i+1,k] <- r_nb_L[k]/(r_nb_L[k] + lamda[i+1,k])

L_st[i+1,k] ~ dnegbin(p_L[i+1,k],r_nb_L[k])

log_L_st[i+1,k] <- log(L_st[i+1,k]+1)

rnd_L[i+1,k] ~ dnorm(0,prec_L_rnd[k]) # random effect for popul. 2:3

eps_L[i+1,k] <- L_mu[i+1,k]-log_L_st[i+1,k]

lemmings[i+1,k] ~ dnorm(L_mu[i+1,k],prec_L_o[prec_pop[k]]) # observation process

}

L_st_hat[i+1] <- (log_L_st[i+1,1]+L_mu[i+1,2]+L_mu[i+1,3])/3

} # end i

## [1,1]

## 1st population, 1965

L_mu[1,1] <- alpha_L_sc[1] + D1[1] + D2[1]

+ chi_L[chi_pop[1]]*L0

+ bC_L_scale[1,1]*Clim[1,1,1]

+ bC_L_scale[2,1]*Clim[1,2,1]

+ rnd_L[1,1]

log(lamda[1,1]) <- L_mu[1,1]

p_L[1,1] <- r_nb_L[1]/(r_nb_L[1] + lamda[1,1])

L_st[1,1] ~ dnegbin(p_L[1,1],r_nb_L[1])

log_L_st[1,1] <- log(L_st[1,1]+1)

rnd_L[1,1] ~ dnorm(0,prec_L_rnd[1]) # random effect for popul. 1

eps_LPearson[1,1] <- (L_mu[1,1]-log_L_st[1,1])/sqrt(var_L_mu[1]) # Pearson residual

eps_L[1,1] <- L_mu[1,1]-log_L_st[1,1]

## Observation process

L_st_mu[1,1] <- log_L_st[1,1] - alpha_peak[1]

lemmings[1,1] ~ dnorm(L_st_mu[1,1], prec_L_o[prec_pop[1]])

eps_L_o[1,1] <- lemmings[1,1]-L_st_mu[1,1]

## 2nd and 3rd population, 1965

for(k in 2:3){

L_mu[1,k] <- alpha_L_sc[k] + L_mu[1,1]

+ bC_L_scale[1,k]*Clim[1,1,k] # scaling

+ bC_L_scale[2,k]*Clim[1,2,k]

+ rnd_L[1,k]

log(lamda[1,k]) <- L_mu[1,k]

p_L[1,k] <- r_nb_L[k]/(r_nb_L[k] + lamda[1,k])

L_st[1,k] ~ dnegbin(p_L[1,k],r_nb_L[k])

log_L_st[1,k] <- log(L_st[1,k]+1)

rnd_L[1,k] ~ dnorm(0,prec_L_rnd[k]) # random effect for popul. 2

eps_L[1,k] <- L_mu[1,k]-log_L_st[1,k]

lemmings[1,k] ~ dnorm(L_mu[1,k],prec_L_o[prec_pop[k]]) # observation process

}

for(k in 1:3){

var_L_mu[k] <- pow(sd(L_mu[,k]),2)

}

L_st_hat[1] <- (log_L_st[1,1]+L_mu[1,2]+L_mu[1,3])/3

# Variance partition, lemmings

var_yr[1] <- pow(beta_yr[yr_pop[1]],2)*pow(sd(yr[]),2)

var_yr[2] <- 0

var_yr[3] <- 0

var_3yr[1] <- pow(sd(D1[]),2)

var_3yr[2] <- pow(sd(D2[]),2)

var_3yr_period[1] <- var_3yr[1] + var_3yr[2]

var_3yr_period[2] <- 0

var_3yr_period[3] <- 0

var_dd_L[1] <- (1-pow(chi_L[chi_pop[1]],2))*pow(sd(log_L_st[,1]),2)

for(k in 2:3){

var_dd_L[k] <- 0

}

sigma_nbL[1] <- sd(eps_L[,1])

sigma_nbL[2] <- sd(eps_L[,2])

sigma_nbL[3] <- sd(eps_L[,3])

for(k in 1:3){

var_eps_L_st[k] <- pow(sd(rnd_L[,k]),2) + pow(sigma_nbL[k],2)

var_clim_L[k] <- pow(bC_L_scale[1,k],2)*pow(sd(Clim[1:53,1,k]),2)

+ pow(bC_L_scale[2,k],2)*pow(sd(Clim[1:53,2,k]),2)

tot_var_L[k] <- var_dd_L[k] + var_eps_L_st[k] + var_clim_L[k] + var_yr[k] + var_3yr_period[k]

var_L_beta1[k] <- pow(bC_L_scale[1,k],2)*pow(sd(Clim[1:53,1,k]),2)

var_L_beta2[k] <- pow(bC_L_scale[2,k],2)*pow(sd(Clim[1:53,2,k]),2)

}

prop_yr <- sum(var_yr[])/sum(tot_var_L[])

prop_clim_L <- sum(var_clim_L[])/sum(tot_var_L[])

prop_dd_L <- sum(var_dd_L[])/sum(tot_var_L[])

prop_eps_L_st <- sum(var_eps_L_st[])/sum(tot_var_L[])

prop_beta_3yr[1] <- var_3yr[1]/sum(tot_var_L[])

prop_beta_3yr[2] <- var_3yr[2]/sum(tot_var_L[])

prop_L_beta1 <- sum(var_L_beta1[])/sum(var_clim_L[])

prop_L_beta2 <- sum(var_L_beta2[])/sum(var_clim_L[])

} # End model

",fill=TRUE)

sink()

save.para=c(

"mu_ratio","logitp_mu","logitp_obs","alpha_juv","sigma_juv","beta_nao3","beta_L","bCj","wj",

"prop.beta_nao3","prop.beta_L","prop.resid_juv","prop.rnd_juv","prop_bCj","eps_juv","Climj",

"alpha_LtD","beta_juv","beta_din","dd_LtD","beta_o_east","sigma_oLTD","sigma_pLTD",

"prop.dd_LtD","prop.beta_juv","prop.beta_din","prop.sigma_pLTD","prop_beta_east","prop_sigma_oLTD",

"state_ltd","epso","eps","wD","d12","prop_obs_error_LtD","log_state_ltd",

"r_nb_ltd","rnd_ltd","sigma_nb_ltd","pool","sigma_pool",

"alpha_L_sc","chi_L","bC_L_scale","wL","L0","sigma_L_o","sigma_L_rnd","prop_clim_L","prop_dd_L",

"prop_yr","beta_yr","prop_L_beta1","prop_L_beta2","rnd_effect_peak","tau0","L_st","L_mu",

"Clim","eps_LPearson","eps_L","eps_L_o","L_st_hat","r_nb_L","prop_eps_L_st","rnd_L","log_L_st",

"beta_3yr","prop_beta_3yr","D1","D2","sigma_nbL"

)

NITER = 400000

n_thin = max(1, floor((NITER - NITER/2) / 500))

set.seed(1744432881)

#### BUGS

bugs5<-jags(data=data_ltd, inits=inits_ltd, parameters.to.save=save.para, n.iter=NITER,

model.file="mod5.r", n.chains=length(inits_ltd), n.thin=n_thin)

## BUGS code for the log model on DIN and DIP amounts in the Danish Straits and Baltic Proper

# Model on DIN and DIP amounts in the Danish Straits and Baltic Proper

sink("modd.r")

cat("

model{

# Parameters and priors

## Intercepts

a0[1] ~ dnorm(0, 1.0E-6) # DIN (state)

a0[2] ~ dnorm(0, 1.0E-6) # DIP (state)

alpha[1,1] <- 0

alpha[1,2] <- 0

alpha[2,1] ~ dnorm(0, 1.0E-6) # BP DIN (observation process)

alpha[2,2] ~ dnorm(0, 1.0E-6) # BP DIP (observation process)

for(j in 1:2){ # area (obs. errors)

for(k in 1:2){ # nutrients (obs. errors)

prec_din[j,k] <- 1/sigma2_din[j,k]

sigma2_din[j,k]<-pow(sigma_din[j,k],2)

sigma_din[j,k]~dunif(0, 100)

}

}

prec_st_din[1] <- 1/pow(sigma_st_din[1],2)

sigma_st_din[1]~dunif(0, 10)

prec_st_din[2] <- 1/pow(sigma_st_din[2],2)

sigma_st_din[2]~dunif(0, 10)

chi_din[1] ~ dnorm(0,1)I(0,)

chi_din[2] ~ dnorm(0,1)I(0,)

lag_theta ~ dbeta(1,1)

rlag ~ dbin(lag_theta, 3)

lag <- 3-rlag

## Priors for splines

for(s in 1:n_knots) {b_Z[1,s] ~ dnorm(0, 1.0E-6)}

for(s in 1:n_knots) {b_Z[2,s] ~ dnorm(0, 1.0E-6)}

b_X[1] ~ dnorm(0, 1.0E-6)

b_X[2] ~ dnorm(0, 1.0E-6)

# Likelihood

## Thin-plate splines (Crainiceanu et al. 2005)

for(i in 1:len){

m_X[i,1] <- b_X[1]*X[i,1] # for DIN

m_X[i,2] <- b_X[2]*X[i,1] # for DIP

}

for (i in 1:len){

for(s in 1:n_knots){ # splines

Z1[i,s,1] <- b_Z[1,s]*Z[i,s] # for splines, DIN

Z1[i,s,2] <- b_Z[2,s]*Z[i,s] # for splines, DIP

}

}

for(i in 1:len){

m_X_spl[i,1] <- sum(Z1[i,,1])

m_X_spl[i,2] <- sum(Z1[i,,2])

}

## End splines

# State process

for(nut in 1:2){

mu_din[1,nut] <- din_1[nut]

st_din[1,nut] ~ dnorm(mu_din[1,nut],prec_st_din[nut])

eps_din[1,nut] <- mu_din[1,nut]-st_din[1,nut]

}

## din_1[1,] 1969 (1-yr lagged)

for(i in 1:(N_din-1)){ # 1970:

for(nut in 1:2){

mu_din[i+1,nut] <- a0[nut] + m_X[i+rlag,nut] + m_X_spl[i+rlag,nut]

+ chi_din[nut]*st_din[i,nut]

st_din[i+1,nut] ~ dnorm(mu_din[i+1,nut],prec_st_din[nut])

eps_din[i+1,nut] <- mu_din[i+1,nut]-st_din[i+1,nut]

}

}

# Observation process

for(i in 1:N_din){

for(area in 1:2){

for(nut in 1:2){

st12[i,area,nut] <- st_din[i,nut] + alpha[area,nut]

din[i,area,nut] ~ dnorm(st12[i,area,nut],prec_din[area,nut])

}

}

}

# Variance partition

for(nut in 1:2){

var.ar_din[nut] <- pow(sigma_st_din[nut],2)/(1-pow(chi_din[nut],2))

var.m_X[nut] <- pow(sd(m_X[1:(N_din-1)+2,nut]),2)

var.m_X_spl[nut] <- pow(sd(m_X_spl[1:(N_din-1)+2,nut]),2)

tot_var_din[nut] <- var.ar_din[nut] + pow(sigma_st_din[nut],2)

+ var.m_X_spl[nut] + var.m_X[nut]

prop.ar_din[nut] <- var.ar_din[nut]/tot_var_din[nut]

prop.m_X_spl[nut] <- var.m_X_spl[nut]/tot_var_din[nut]

prop.m_X[nut] <- var.m_X[nut]/tot_var_din[nut]

prop.sigma_st_din[nut] <- pow(sigma_st_din[nut],2)/tot_var_din[nut]

}

######## Variance partition, observation error ########

tot_obs_error <- pow(sigma_din[1,1],2) + pow(sigma_din[2,1],2)

+ pow(sigma_din[1,2],2) + pow(sigma_din[2,2],2)

for(area in 1:2){

for(nut in 1:2){

prop_obs_error[area,nut] <- pow(sigma_din[area,nut],2)/tot_obs_error

}

}

} # End model

",fill=TRUE)

sink()

save.para=c(

"alpha","a0","chi_din","sigma_din","prop.ar_din","st12","st_din","eps_din","din",

"prop.sigma_st_din","sigma_st_din","lag_theta","lag","prop.m_X_spl","m_X_spl","b_X",

"b_Z","prop.m_X","prop_obs_error"

)

NITER = 80000

set.seed(-608056801)

#### BUGS

bugs_din<-jags(data=data_din, inits=inits_din, parameters.to.save=save.para, n.iter=NITER,

model.file="modd.r", n.chains=length(inits_din), n.thin=100)

## BUGS code for the hierarchical model on the blue mussel biomasses

# Model on blue mussel biomasses

sink("modm.r")

cat("

model

{

# Parameters and priors

scaling ~ dnorm(0, 1.0E-6) # scaling for observation, population 2

a0[1] ~ dnorm(10.596635, 1) # initial population 1: log(40000) [10.596635], series 1: 1986

b0 ~ dnorm(0, 1.0E-6) # intercept, state

chi~dunif(0, 1)

prec_st<-1/sigma2_st

sigma2_st<-pow(sigma.st,2)

sigma.st~dunif(0, 5)

for(j in 1:max(obs_j)){

prec_obs[j]<-1/sigma2_obs[j]

sigma2_obs[j]<-pow(sigma_obs[j],2)

sigma_obs[j]~dunif(0, 10)

prec_dem[j]<-1/sigma2_dem[j]

sigma2_dem[j]<-pow(sigma_dem[j],2)

sigma_dem[j]~dunif(0, 10)

}

b1_Tw ~ dnorm(0, 1.0E-6) # temperature, winter

b_fer ~ dnorm(0, 1.0E-6) # fertilizer

weight~dbeta(1,1)

lag_theta ~ dbeta(1,1)

rm_fer ~ dbin(lag_theta, 3)

lag_m_fer <- 3-rm_fer

for(s in 1:n_knots){ # splines

b_fZ[s] ~ dnorm(0, 1.0E-6)

}

for(i in 1:33){

Twi[i] <- (sum(T_mx_Wi[i,]) + sum(T_mn_Wi[i,]))/6 # [1] refers to 1984

}

for(i in 1:32){

wTwi[i] <- (1-weight[1])*Twi[i] + weight[1]*Twi[i+1]

}

## Thin-plate splines (Crainiceanu et al. 2005)

for (i in 1:len){

for(s in 1:n_knots){ # splines

Z1[i,s] <- b_fZ[s]*Z[i,s]

}

}

for(i in 1:len){

m_F_spl[i] <- sum(Z1[i,])

}

## End splines

for(i in 1:(len-3)){

F[i] <- fer[i+rm_fer] # fer[1] refers to 1987-3

F_spl[i] <- m_F_spl[i+rm_fer]

}

# State process

mus_mu[1] <- a0[1] # 1986

st1[1] ~ dnorm(mus_mu[1],prec_st)

st_dem[1,1] ~ dnorm(st1[1],prec_dem[1])

st_dem[1,2] ~ dnorm(st1[1],prec_dem[2])

st2[1,1] <- st_dem[1,1]

st2[1,2] <- st_dem[1,2] + scaling

eps_1[1] <- mus_mu[1] - st2[1,1]

# Observation process

mus2[1,1] ~ dnorm(st2[1,1],prec_obs[1])

mus2[1,2] ~ dnorm(st2[1,2],prec_obs[2])

for(i in 1:(N-1)){ # process 1987-2017, [1,] refers to 1986

mus_mu[i+1] <- b0 + chi*st2[i,1]

+ b1_Tw*wTwi[i]

+ b_fer* F[i]

+ F_spl[i]

st1[i+1] ~ dnorm(mus_mu[i+1],prec_st) # environmental variance

# Demographic stochasticity

st_dem[i+1,1] ~ dnorm(st1[i+1],prec_dem[1])

st_dem[i+1,2] ~ dnorm(st1[i+1],prec_dem[2])

st2[i+1,1] <- st_dem[i+1,1]

st2[i+1,2] <- st_dem[i+1,2] + scaling

eps_1[i+1] <- mus_mu[i+1] - st2[i+1,1]

# Observation model

mus2[i+1,1] ~ dnorm(st2[i+1,1],prec_obs[1])

mus2[i+1,2] ~ dnorm(st2[i+1,2],prec_obs[2])

}

# Variance partition

var_Twi <- pow(b1_Tw,2)*pow(sd(wTwi[1:(N-1)]),2)

var_fer <- pow(b_fer,2)*pow(sd(F[]),2)

var_fer_spl <- pow(sd(F_spl[]),2)

var_dd <- s_var*(1-pow(chi,2))

var_dem <- sigma_dem[1] * sigma_dem[2]

tot_var <- var_dd + sigma2_st + var_Twi + var_fer + var_dem + var_fer_spl

tot_minus_spl <- tot_var-var_fer_spl

prop_m_dd <- var_dd/tot_minus_spl

prop_sigma_st <- sigma2_st/tot_minus_spl

prop1_T_winter <- var_Twi/tot_minus_spl

prop2_fer <- var_fer/tot_minus_spl

prop_sigma_dem <- var_dem/tot_minus_spl

prop_spl_of_tot <- var_fer_spl/tot_var

} # end model

",fill=TRUE)

sink()

save.para=c(

"chi","weight","a0","b0","eps_1","st1","st2","mus2","sigma_obs","prop_m_dd","prop1_T_winter",

"prop2_fer","wTwi","b1_Tw","b_fer","b_fZ","sigma.st","prop_sigma_st","prop_sigma_dem",

"lag_m_fer","prop_spl_of_tot","sigma_dem","F_spl","F"

)

set.seed(1823176837)

#### BUGS

NITER = 400000

n_thin = max(1, floor((NITER - NITER/2) / 500))

bugsm<-jags(data=data_mus, inits=inits_mus, parameters.to.save=save.para, n.iter=NITER, n.thin=n_thin,

model.file="modm.r", n.chains=length(inits_mus))

## BUGS code for the hierarchical model on the blue mussel flesh/shell ratios

# Model on blue mussel flesh/shell ratios

sink("mod_fs.r")

cat("

model

{

# Parameters and priors

for(j in 1:n_f_beta){

f_beta[j] ~ dnorm(0, 1.0E-6)

}

alpha_f ~ dnorm(0, 1.0E-6)

prec_f<-1/sigma2_f

sigma2_f<-sigma_f*sigma_f

sigma_f~dunif(0, 10)

prec_f_rnd<-1/sigma2_f_rnd

sigma2_f_rnd<-pow(sigma_f_rnd,2)

sigma_f_rnd~dunif(0, 10)

lag_theta ~ dbeta(1,1)

r_fer ~ dbin(lag_theta, 2)

lag_fer <- 2-r_fer

# Likelihood

for(i in 1:N_f){

Twi_fs[i] <- (sum(fsT_mx_Wi[i,]) + sum(fsT_mn_Wi[i,]))/6

Tsp_fs[i] <- (sum(fsT_mx_Sp[i,]) + sum(fsT_mn_Sp[i,]))/6

}

for(i in 1:N_f){

wF_fs[i] <- fer_fs[i+r_fer] # fer_fs[1] refers to 1998-2

}

for(i in 1:N_f){ # logit_f_mu[1] refers to 1998

logit_f_mu[i] <- alpha_f

+ f_beta[1]* Twi_fs[i]

+ f_beta[2]* Tsp_fs[i]

+ f_beta[3]* wF_fs[i]

+ eps_rnd_f[i]

logit_f_obs[i] ~ dnorm(logit_f_mu[i],prec_f)

eps_rnd_f[i] ~ dnorm(0,prec_f_rnd)

pf[i] <- exp(logit_f_mu[i])/(1+exp(logit_f_mu[i]))

log_pf[i] <- log(pf[i])

Rf[i] <- pf[i]+1

ratio_f[i] <- pf[i]/(1-pf[i])

log_ratio_f[i] <- log(ratio_f[i])

eps_f[i] <- logit_f_obs[i] - logit_f_mu[i]

}

# Variance partition

var_f_beta[1] <- pow(f_beta[1],2)*pow(sd(Twi_fs[1:N_f]),2)

var_f_beta[2] <- pow(f_beta[2],2)*pow(sd(Tsp_fs[1:N_f]),2)

var_f_beta[3] <- pow(f_beta[3],2)*pow(sd(wF_fs[1:N_f]),2)

tot_var <- var_f_beta[1]+var_f_beta[2]+var_f_beta[3]+sigma2_f+sigma2_f_rnd

prop_f_beta[1] <- var_f_beta[1]/tot_var

prop_f_beta[2] <- var_f_beta[2]/tot_var

prop_f_beta[3] <- var_f_beta[3]/tot_var

prop_f_sigma <- sigma2_f /tot_var

prop_f_rnd <- sigma2_f_rnd /tot_var

} # end model

",fill=TRUE)

sink()

save.para=c(

"alpha_f","f_beta","ratio_f","log_ratio_f","sigma_f",

"prop_f_beta","prop_f_sigma","eps_f","wF_fs","Twi_fs",

"sigma_f_rnd","prop_f_rnd","logit_f_obs","lag_fer"

)

NITER = 200000

n_thin = max(1, floor((NITER - NITER/2) / 1000))

set.seed(-205983225)

#### BUGS

bugsfs<-jags(data=data_fs, inits=inits_fs, parameters.to.save=save.para,

n.iter=NITER, n.thin=n_thin, model.file="mod_fs.r", n.chains=length(inits_fs))
